# Aromatic ring flips reveal reshaping of protein dynamics in crystals and complexes

**DOI:** 10.1038/s41557-026-02155-0

**Published:** 2026-06-10

**Authors:** Lea M. Becker, Haohao Fu, Ben P. Tatman, Matthias Dreydoppel, Anna Kapitonova, Daniel M. Balazs, Ulrich Weininger, Sylvain Engilberge, Christophe Chipot, Paul Schanda

**Affiliations:** 1 Institute of Science and Technology Austria, Klosterneuburg, Austria; 2 Tianjin Key Laboratory of Biosensing and Molecular Recognition, Research Center for Analytical Science, Frontiers Science Center for New Organic Matter, College of Chemistry, Nankai University, Tianjin, China; 3 Institute of Physics, Biophysics, Martin-Luther-University Halle-Wittenberg, Halle (Saale), Germany; 4 Univ. Grenoble Alpes, CNRS, CEA, Institut de Biologie Structurale, Grenoble, France; 5 Laboratoire International Associé CNRS and University of Illinois at Urbana-Champaign, LPCT, UMR 7019 Université de Lorraine CNRS, Vandoeuvre-les-Nancy, France; 6 Department of Physics, University of Illinois at Urbana-Champaign, Urbana, IL USA; 7 Department of Biochemistry and Molecular Biology, The University of Chicago, Chicago, IL USA

**Keywords:** Solid-state NMR, Solution-state NMR, X-ray crystallography, Molecular modelling

## Abstract

Protein conformational energy landscapes are shaped not only by intramolecular interactions but also by their environment. In protein crystals and protein–protein complexes, intermolecular contacts alter this energy landscape, but the exact nature of this alteration is difficult to decipher. Understanding how the crystal lattice affects protein dynamics is crucial for crystallography-based studies of motion, yet its influence on collective motions remains unclear. Aromatic ring flips in the hydrophobic core represent sensitive probes of such dynamics. Here, we compare the kinetics of aromatic ring flips in the protein GB1 in crystals, in complex with its binding partner IgG, and in solution, combining advanced isotope labelling with quantitative NMR methods. We show that rings in the core flip nearly a thousand times less frequently in crystals than in solution. Enhanced-sampling molecular dynamics simulations, based on a crystal structure of a GB1 variant reported in this work, reproduce these elevated barriers and reveal how the crystal restrains motions.

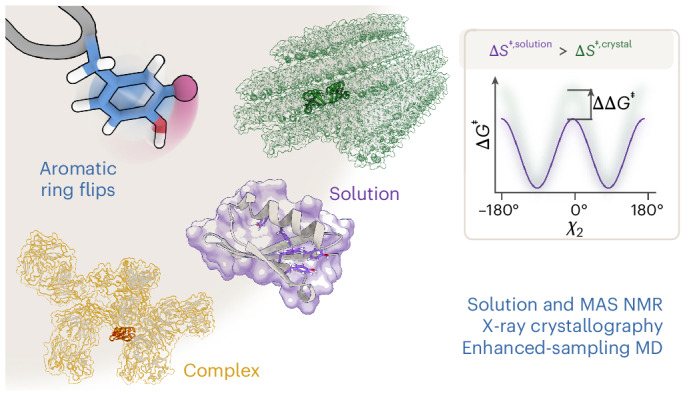

## Abstract (web summary)

The extent to which crystal packing constrains protein dynamics is hard to decipher. Now a combination of NMR, crystallography and molecular dynamics simulations show that intermolecular contacts can slow ring flips down in crystals or accelerated them in complexes. A thermodynamic and structural analysis provides insights into the origins of the elevated free-energy barrier in crystals.

## Main

Protein crystallography has been the central pillar of structural biology for over half a century, with two hundred thousand structures determined up to now^1^. It has been recognized early on that these seemingly static images are misleading; as G. Weber expressed in 1975, proteins in solution are ‘kicking and screaming stochastic molecules’^2^. Meanwhile, proteins in crystal lattices still exhibit substantial motion, which can be indirectly observed via residue-wise B-factor variability, blurring of the electron density or co-existing conformations (so-called alternate locations)^3–5^. A long-standing question is to what extent crystal packing constrains protein dynamics relative to solution^6,7^. Early studies of enzymatic activity^8–14^ revealed a close match between crystal and solution in some cases^15^, and strongly attenuated activity in others^8^. Comparisons of different crystal forms established that packing can alter both structure and dynamics^16–18^. Today, serial crystallography at X-ray free-electron lasers and synchrotrons enables the capture of protein kinetics and mechanisms in crystals at room temperature with up to femtosecond time resolution^19–30^. These experiments build upon the assumption that the crystal lattice does not influence protein dynamics and underline the relevance of this question once again. Crystallographic methods can naturally not assess the dynamics of non-crystalline samples, and therefore cannot quantify constraints of the lattice on protein motions relative to solution.

Molecular dynamics (MD) simulations of entire crystal unit cells show that proteins retain substantial internal flexibility^31–37^. However, MD currently accesses only motions up to the μs regime, leaving slower conformational changes, often central to function, difficult to capture.

Nuclear magnetic resonance (NMR) spectroscopy can probe protein dynamics in various environments. Comparisons of solution-state NMR with magic-angle spinning (MAS) NMR of protein crystals yield direct insights into the modulation of motions by the crystal lattice. Spin-relaxation experiments showed that fast (sub-μs) local motions are largely preserved in crystals, except in loops engaged in packing contacts^18,38,39^. Slower motions on the μs–ms timescale seem to be more strongly perturbed: the *β*-turn in ubiquitin is slowed by more than an order of magnitude in crystals, and the populations are space group dependent^40,41^.

**Fig. 1:**
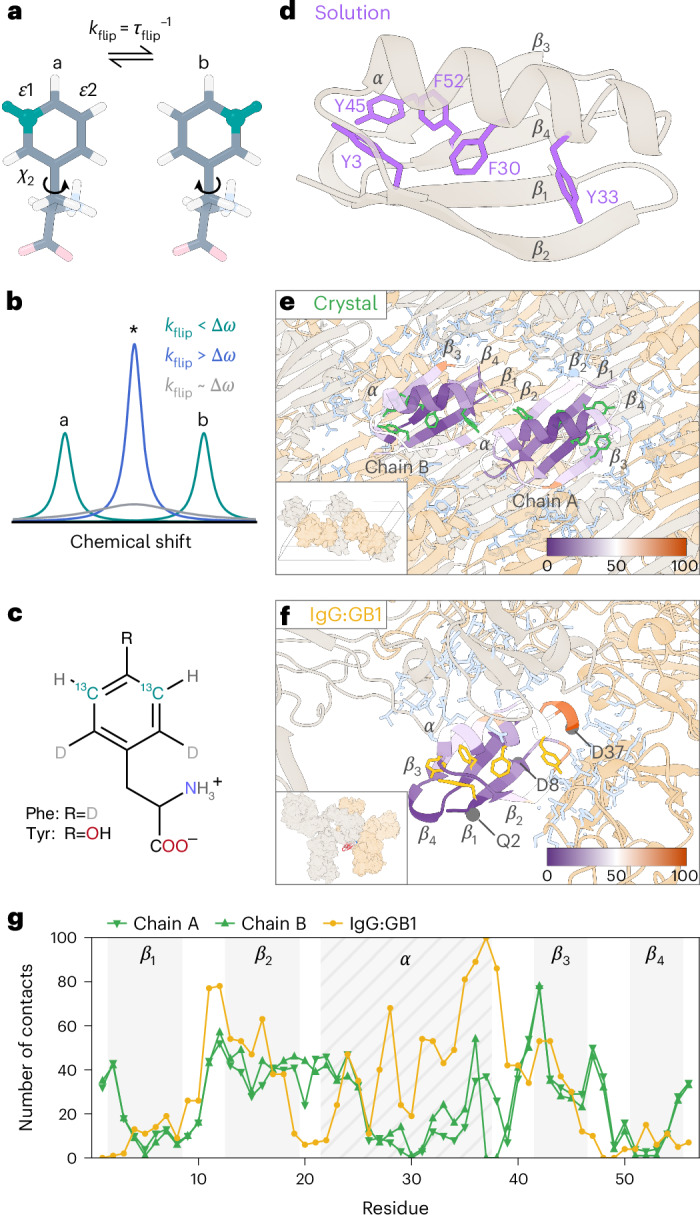
Specific isotope labelling of aromatic residues in different forms of GB1. **a**, Schematic representation of a ring flip. The *χ*_2_ angle around the C^*β*^–C^*γ*^ bond rotates 180° with a flip rate *k*_flip_. (CH)^*ϵ*1^ and (CH)^*ϵ*2^ exchange their position in the ring, creating states a and b. **b**, Schematic timescale-dependent NMR signatures resulting from flips of (CH)^*ϵ*^-labelled Phe or Tyr residues. Signals of sites a and b are resolved if *k*_flip_ is slower than the chemical shift difference Δ*ω* (cyan). If *k*_flip_ > Δ*ω*, the signals are averaged (blue). At intermediate timescales (*k*_flip_ ~ Δ*ω*), the signal is broadened (grey). **c**, *α*-Ketoacid precursor for 3,5-^13^CH labelling of the aromatic ring of Phe and Tyr^47^. **d**, Structure of GB1_QDD_ in cartoon representation with Phe and Tyr as purple sticks. Secondary structure elements are indicated in grey. **e**, Surrounding of GB1_QDD_ (asymmetric unit with chains A and B) in the crystal lattice. The backbone colour represents the graph in **g**. Atoms within 10 Å of C^*α*^ atoms are shown in light blue sticks. The insert shows one unit cell with A and B molecules in different colours. **f**, Surrounding of GB1_QDD_ in IgG:GB1 (backbone colour as explained above). C^*α*^ atoms of Q2, D8 and D37 are shown as grey spheres. The insert shows an overview of the complex (modelled as described in ref. ^53^). **g**, Number of atoms within 10 Å of C^*α*^ atoms in chains A and B of the crystal and the IgG:GB1 complex. Secondary structure elements are indicated as in the structure representations in **d**–**f**.

Aromatic rings of phenylalanines and tyrosines undergo flips around the C^*β*^–C^*γ*^ axis (the *χ*_2_ angle; Fig. 1a)^42–44^ and are particularly sensitive probes of collective dynamics. Buried rings are thought to require large, cooperative motions of the protein to flip, called ‘breathing motions’^45,46^. Because the two interchanging states are structurally indistinguishable, crystallography cannot detect these flips. By contrast, they are easily observable by NMR, since the ^1^H and ^13^C spins on each side of the aromatic ring (for example, *ϵ*_1_ and *ϵ*_2_) differ in chemical shift. A ring flip consequently leads to an interchange of (CH)^*ϵ*1^ and (CH)^*ϵ*2^ as well as (CH)^*δ*1^ and (CH)^*δ*2^, resulting in timescale-dependent spectral signatures (Fig. 1b). The introduction of *α*-ketoacid precursors (Fig. 1c) for site-specific labelling of the (CH)^*ϵ*^ atoms has greatly enhanced the precision of such studies^47^.

Here we study aromatic ring flips to assess how protein–protein interactions modify dynamics. We focus on the immunoglobulin-binding domain of protein G (GB1), studied in three contexts: in solution (Fig. 1d), the crystalline state (Fig. 1e), and in complex with its natural binding partner, the immunoglobulin G (IgG; Fig. 1f). Y3, F30, Y45 and F52 form a hydrophobic aromatic cluster, while Y33 is exposed to the solvent (Fig. 1d). The commonly studied T2Q variant (GB1_T2Q_) has been extensively characterized structurally and by solution and MAS NMR. GB1_QDD_, a stabilized triple mutant (T2Q, N8D and N37D)^48^, is structurally uncharacterized, but temperature- and pressure-dependent solution NMR results provide a detailed baseline for comparison^49,50^.

Our integrated approach, combining solution and MAS NMR, crystallography and multi-μs MD simulations, shows that crystal packing slows ring flips of buried residues by approximately three orders of magnitude. A thermodynamic and structural analysis of this observation provides insights into the origins of the elevated free-energy barrier in crystals. Remarkably, in the IgG complex, the same ring flips are much faster than in crystals, highlighting how the precise nature of intermolecular contacts reshapes the underlying free-energy landscape.

## Results

### The packing of GB1_QDD_ in crystals and the IgG:GB1_QDD_ complex

Precise atomic-resolution structures are important for the mechanistic interpretation of protein dynamics. To our knowledge, crystallization of GB1_QDD_ has not been previously reported. The GB1_T2Q_ variant yields crystals in the *P*3_2_21 space group, suitable for solid-state NMR studies^51^. Crystallization of GB1_QDD_ reproducibly failed under these conditions, which we attribute to a disruption of packing interactions by the additional surface-exposed N8D and N37D mutations. Instead, we obtained GB1_QDD_ crystals in the *C*121 space group using a dialysis-based method^52^. These crystals were suitable for MAS NMR and single-crystal X-ray diffraction (XRD), depending on the duration of crystallization (Supplementary Figs. 1 and 2; see Methods for details). Single-crystal XRD data were collected at a resolution of 1.08 Å at 100 K, and 1.69 Å at 295 K (Supplementary Table 1), revealing an asymmetric unit with two molecules (Fig. 1e).

A structural comparison between the crystal and complex revealed differences in intermolecular packing. We determined the number of non-hydrogen atoms within 10 Å of each C^*α*^ atom for chains A and B in the crystal (Fig. 1e) and in a model of the IgG:GB1 complex^53–55^ (Fig. 1f). The two crystallographic chains exhibit very similar packing densities (Fig. 1g, green), but the distribution of contacts differs from that in the complex (yellow). These differences in the local packing could potentially modulate protein dynamics.

### MAS NMR of GB1_QDD_ crystals

Equipped with this structural knowledge, we prepared crystals with different isotope-labelling schemes. For studies of aromatic residues, we used metabolic precursors to obtain isolated (^13^C^1^H)^*ϵ*^ spin pairs in phenylalanines and tyrosines in addition to uniform ^2^H and ^15^N labelling (Fig. 1c; see Methods for details)^47^. The protein used for backbone assignment was labelled uniformly with ^2^H, ^13^C and ^15^N (exchangeable hydrogens were ^1^H).

We sought to verify that the crystals used for NMR and single-crystal XRD crystallized with the same packing. Powder XRD measurements on the same wet microcrystalline samples as used for NMR, in comparison with the single-crystal XRD structure, confirmed that the crystals represent the same phase (Supplementary Fig. 3).

**Fig. 2:**
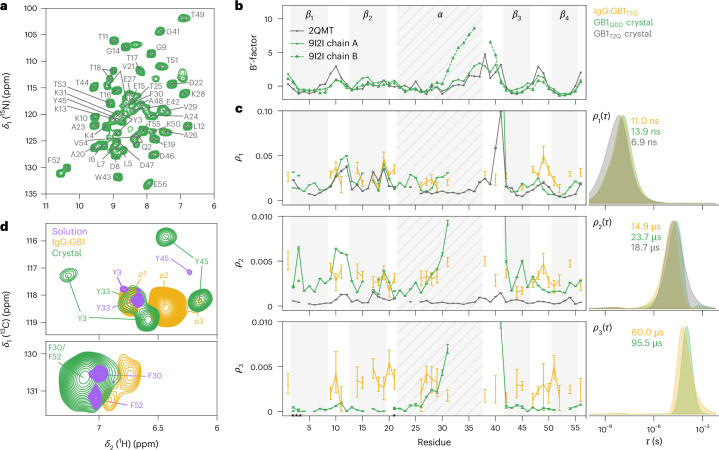
MAS NMR of different forms of GB1. **a**, Dipolar-based ^1^H–^15^N NMR spectra of crystalline GB1_QDD_ with assignment. **b**, Normalized B-factors of the GB1_T2Q_ (grey, PDB 2QMT) and the GB1_QDD_ (green, PDB 9I2I) crystal structures (normalization with BANΔIT online tool^82^; see Supplementary Fig. 2 for raw B-factors). **c**, Detectors analysis of backbone-amide ^15^N spin-relaxation data for the IgG:GB1_T2Q_ complex (yellow, 300 K)^57,59,60^, GB1_QDD_ microcrystals (green, 304 K) and GB1_T2Q_ microcrystals (grey, 300 K)^57,58^. The panels on the left show residue-wise responses to the corresponding detector sensitivities *ρ*_1−3_(*τ*) on the right. *ρ*_1−3_(*τ*) differ slightly for each sample state, as the recorded relaxation rates were different. The error bars of the responses represent the 5–95% interpercentile range for Monte Carlo simulations with 200 iterations. **d**, Tyrosine (upper panel) and phenylalanine (lower panel) region of ^1^H–^13^C solution-state or dipolar-based MAS NMR spectra of (^13^C^1^H)^*ϵ*^-labelled GB1 in solution (purple), a complex with IgG (yellow) and a microcrystal (green).

As GB1_QDD_ crystals have not been studied previously by MAS NMR, we performed experiments to assign the H^N^, N^H^, C′, C^*α*^ and C^*β*^ resonances (see Methods for details). The quality of the data enabled automatic assignments for most of the protein (Fig. 2a, Supplementary Table 3, and Supplementary Figs. 5 and 6, BMRB access code 53330). We observed two distinct sets of peaks for four residues (Q2, Y3, T18 and V21), which we ascribe to the two non-equivalent molecules. A Na^+^ ion bound in the vicinity of one of the two chains might explain the differences.

Interestingly, signals of residues Q32–D40 were unobservable in the spectra, likely owing to dynamics-induced line broadening (discussed below). Residues D37 and G38 in chain B of the crystal structure could not be modelled owing to blurring of the electron density, and crystallographic B-factors are generally high in this region (Fig. 2b). In previous MAS NMR studies of GB1_T2Q_ crystals and IgG-bound GB1_T2Q_, residues Q32–D40 were observable, and B-factors in the GB1_T2Q_ crystal are uniform. These findings suggest crystal-packing-dependent differences in the dynamics of this protein region.

To quantitatively characterize the GB1_QDD_ crystal dynamics, we performed ^15^N spin-relaxation experiments (longitudinal *R*_1_ relaxation and rotating-frame *R*_1*ρ*_ relaxation at six different spin-lock frequencies) and analysed them using the detectors approach^56–60^ (Fig. 2c, green, and Supplementary Figs. 11 and 12). For comparison, we show previously published data for the GB1_T2Q_ crystal (grey)^57,58^ and the IgG:GB1_T2Q_ complex^57,59,60^ (yellow). The detectors report on the amount of motion within certain time windows, so-called detector sensitivities *ρ*_*i*_(*τ*) (Fig. 2c, right; see Methods for details). The position of these windows reflects the timescales to which the measured relaxation rate constants are sensitive. The responses *ρ*_*i*_ (Fig. 2c, left) report the motional amplitude of each residue in the respective sensitivity *ρ*_*i*_(*τ*).

The most striking feature is the elevated μs–ms motion for residues 28–31 and 41 (seen as a sharp increase of *ρ*_2_ and *ρ*_3_) supporting the conclusion that residues Q32–D40 are broadened owing to enhanced microsecond motions^61^. In GB1_T2Q_, this motion is not observed in crystals (Fig. 2c, grey) or residual-dipolar coupling analyses in solution^62^. While fast motions (*ρ*_1_) are similar in the three samples, presumably owing to their local nature, there is an overall increase of *ρ*_1_ and *ρ*_2_ in GB1_QDD_ and IgG:GB1, compared with GB1_T2Q_. For IgG:GB1, these dynamics have been ascribed to the overall rocking motion of the protein^63^.

### NMR signatures of aromatics in three different states of GB1_QDD_

We obtained high-resolution dipolar-based ^1^H–^13^C two-dimensional (2D) MAS NMR spectra of crystalline GB1_QDD_ and the IgG:GB1_QDD_ complex and compared them with solution-state NMR spectra of GB1_QDD_ (Fig. 2d). Experiments connecting (^13^C^1^H)^*ϵ*^ sites to surrounding amide signals (radio frequency-driven recoupling^64^) allowed us to assign all tyrosine signals in the crystal (Supplementary Fig. 7); only one broad, unresolved peak is observed for F30 and F52 (Fig. 2d, lower panel).

The low sensitivity of the IgG:GB1_QDD_ sample precluded residue-specific assignments of aromatic signals. We nonetheless confirmed that the Tyr peaks arise from individual residues via ^13^C–^13^C exchange spectroscopy (EXSY; Supplementary Fig. 10). The absence of cross-peaks indicates that each signal originates from an individual residue, rather than from slow exchange. We, therefore, refer to the three Y^*ϵ*^ peaks of IgG:GB1 as p1, p2 and p3 in the following.

### Differences in aromatic spectra show altered ring-flip dynamics

The number of signals observed for a (^13^C^1^H)^*ϵ*^ site provides a semi-quantitative view of the ring-flip rate constant *k*_flip_ (Fig. 1b). For GB1_QDD_ in solution, each aromatic residue gives rise to one averaged signal because *k*_flip_ > Δ*ω*, where Δ*ω* is the chemical shift difference between (CH)^*ϵ*1^ and (CH)^*ϵ*2^ (Fig. 2d, purple). *k*_flip_ is a few tens of thousands per second for the buried rings, determined previously by relaxation-dispersion experiments at 298 K (Y3, (13,700 ± 1,600) s^−1^; Y45, (42,100 ± 21,700) s^−1^; F30, (29,600 ± 1,500) s^−1^; F52, (34,400 ± 4,500) s^−1^)^49^. The solvent-exposed Y33 flips too rapidly for quantification by solution NMR relaxation-dispersion experiments, implying that *k*_flip_ ≳ 100,000 s^−1^.

Similar to the situation in solution, a single set of peaks is observed in the IgG:GB1 complex (Fig. 2d, yellow).

The spectrum of the crystalline sample exhibits a very different signature (Fig. 2d, green). Y3 and Y45 each show two peaks split equidistantly around their position in solution, suggesting slower flips in the crystal than in solution, with *k*_flip_ < Δ*ω*. The solvent-exposed Y33 exhibits a single signal indicating fast flips. In contrast to solution, the phenylalanine region shows one broad signal pointing to μs motion of F30 and/or F52 in crystals.

### Crystal packing slows flips of buried tyrosine rings

**Fig. 3:**
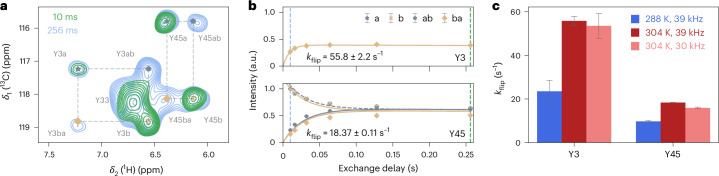
Determination of ring-flip timescales of Y3 and Y45 in crystalline GB1_QDD_. **a**, ^1^H–^13^C EXSY MAS NMR spectrum of crystalline GB1 with 0 ms (green) and 256 ms (light blue) exchange delay at 39 kHz MAS. **b**, Normalized signal intensities depending on the exchange delay and fitted EXSY build-up and decay curves for Y3 and Y45. For Y3, only the build-up of ‘ba’ was fitted (see text). For Y45, we performed a combined analysis of all four peaks with *p*_a_ = *p*_b_ = 0.5 (ref. ^66^). The errors of the extracted intensities are one standard deviation of the spectral noise level. **c**, Temperature and MAS dependence of *k*_flip_ for Y3 and Y45. Error bars of *k*_flip_ were determined by Monte Carlo error analysis as one standard deviation of 300 iterations (see Methods for details).

To quantify *k*_flip_ of Y3^crystal^ and Y45^crystal^, we performed ^1^H–^13^C EXSY measurements^65^ (Fig. 3). This type of experiment probes the kinetics of conformational exchange via the respective build-up and decay of cross and diagonal peaks during an increasing exchange delay, *τ*_ex_. Even though the symmetric labelling results in equally populated states with *p*_a_ = *p*_b_ = 0.5, the intensities of Y3a and Y3b and Y3ab and Y3ba differ (Fig. 3a and Supplementary Fig. 13). Reasons for this could be partial peak overlap with Y33, or differences in ^1^H relaxation and cross-polarization efficiency between the two sites. We therefore only fitted Y3ba for this residue (Fig. 3b). For Y45, we performed a combined fit of all four signals^66^. The extracted flip rates for Y3 and Y45 are (55.8 ± 2.2) s^−1^ and (18.37 ± 0.11) s^−1^ at 304 K, about 3 orders of magnitude slower than in solution.

To ensure that the cross-peaks are not a result of magnetization transfer (spin diffusion), we performed experiments at different MAS frequencies and temperatures (Fig. 3c and Supplementary Fig. 13). Magnetization transfer in MAS NMR—which would occur even in static systems^67^—depends on the MAS frequency, whereas ring-flip dynamics are expected to depend on temperature. The observed build-up rate constants are found to only depend on temperature, confirming that the observed process is indeed ring-flip dynamics (see Methods for details).

### Probing fast-flipping rings by MAS NMR methods

**Fig. 4:**
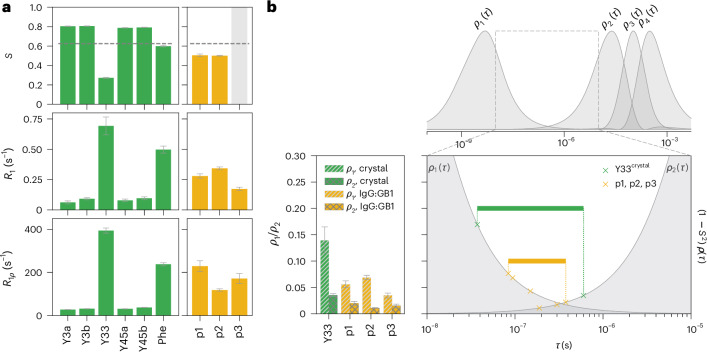
Determination of ring-flip timescales of fast-flipping tyrosines in GB1. **a**, Dipolar order parameters *S* (top), ^13^C^*ϵ*^ *R*_1_ (middle) and *R*_1*ρ*_ (bottom, spin-lock field strength *ν*_SL_ of 15 kHz) relaxation rate constants of Tyr and Phe residues in crystalline GB1 (left) and in the IgG:GB1 complex (right). The dashed line (upper panel) indicates the value of the expected order parameter for a ring flip *S* = 0.625 (ref. ^69^). The order parameter of p3 could not be determined owing to low sensitivity (grey box). Errors of *S*, *R*_1_ and *R*_1*ρ*_ were determined by Monte Carlo error analysis as one standard deviation of 500 iterations based on one standard deviation of the spectral noise level (see Methods for details). **b**, Estimation of ring-flip rates for tyrosines Y3^crystal^, p1, p2 and p3 (see also Supplementary Note 1 and Supplementary Fig. 20). The upper panel shows the detector sensitivities *ρ*_1−4_(*τ*). The lower left panel shows the responses *ρ*_1_ and *ρ*_2_ for Y3^crystal^, p1, p2 and p3. The lower right panel shows a close-up of the area indicated by a dashed box with sensitivities scaled by (1 − *S*^2^) (*S* = 0.625). Crosses mark the intersection between the responses and the scaled sensitivities. Horizontal bars indicate the range of timescales for the ring flips. Error bars of the responses represent the 5–95% interpercentile range for Monte Carlo simulations with 200 iterations.

EXSY is not applicable to fast-flipping rings that show only a single averaged signal for the two (^13^C^1^H)^*ϵ*^ sites. To gain information on the dynamics of these rings, we measured dipolar order parameters *S* and ^13^C spin relaxation (Fig. 4). Order parameters range from 1 for totally rigid to 0 for flexible disordered sites and report on amplitudes of motion on timescales up to hundreds of microseconds^68^. The theoretical value for a ring flip, modelled by simulations, is *S* = 0.625 (ref. ^69^); reductions below this value point to additional dynamics, such as librational, backbone or additional side-chain motion (that is, around the *χ*_1_ angle). Rotational-echo double-resonance (REDOR) experiments (Fig. 4a and Supplementary Figs. 14 and 15) resulted in *S* ≈ 0.8 for Y3 and Y45 in the crystal. The fact that these values exceed the theoretical value confirms that the flips of Y3 and Y45 are slower than the timescale sensed by REDOR, that is, about hundreds of microseconds, in agreement with the EXSY data. By contrast, the order parameters of Y33 and the phenylalanines are lower than the expected value (approximately 0.3 and 0.5, respectively), showing that there must be motion in addition to ring flips, occurring faster than hundreds of microseconds (see Supplementary Note 1 and Methods for details).

Interestingly, the electron density map of the room-temperature crystal structure provides support for such additional motion of the ring axis: it reveals alternate conformations of Y33 with the aromatic ring rotated outwards around *χ*_1_ (Supplementary Fig. 16). Crystallography alone cannot differentiate between conformations statically locked by the crystal lattice and dynamic interconversion. NMR order parameters and the appearance of only one signal confirm fast motion (sub-100 μs) around both *χ*_1_ and *χ*_2_ angles of Y33 in the crystal. Only this combined analysis can explain the observations from both methods and yield a comprehensive picture.

To quantify the ring-flip timescales more precisely, we used ^13^C spin-relaxation experiments (*R*_1_ and *R*_1*ρ*_ at six different spin-lock frequencies). The markedly different relaxation behaviour of Y33, compared with the slowly flipping Y3 and Y45, indicates faster dynamics (Fig. 4a). We used the detectors approach^56,57^ to determine the amplitude of motion within each of the four detector sensitivities *ρ*_1−4_ (Fig. 4b, upper panel, and Supplementary Figs. 17, 18 and 20). The theoretically expected response for a ring flip allowed us to define the upper and lower bounds of the ring-flip timescale for Y33^crystal^, which are 37 ns and 600 ns, respectively (Fig. 4b, lower panels, and Supplementary Fig. 8; see Supplementary Note 1 and Methods for details).

The broad Phe signal (F30 and/or F52) has an order parameter on the order of the expected value, which points to ring flips shorter than hundreds of microseconds. The high *R*_1_ and *R*_1*ρ*_ relaxation rate constants correspond to a ns–μs timescale, consistent with the large line width. Extracting site-specific information for F30 and F52 is not possible owing to signal overlap; therefore, we focus on the tyrosines in the following discussion.

### Ring flips in IgG:GB1_QDD_ are faster than in crystals

The ^1^H–^13^C 2D MAS NMR spectra suggest different ring-flip dynamics in IgG:GB1_QDD_ than in the crystal. The observation of one peak per tyrosine points to fast ring flips (Fig. 2d) supported by low dipolar order parameters and elevated ^13^C *R*_1_ and *R*_1*ρ*_ relaxation rate constants (Fig. 4a and Supplementary Fig. 19). The detectors analysis of these relaxation data (see above) resulted in a timescale estimate of the ring flips between tens and hundreds of nanoseconds (Fig. 4b and Supplementary Fig. 20) and is similar for all three Tyr peaks (p1, p2 and p3). Despite the lack of residue-specific assignments, we can conclude that the tyrosines in IgG:GB1_QDD_ flip about five orders of magnitude faster than in the crystal.

### Ring flips in crystals are less entropically favoured

**Fig. 5:**
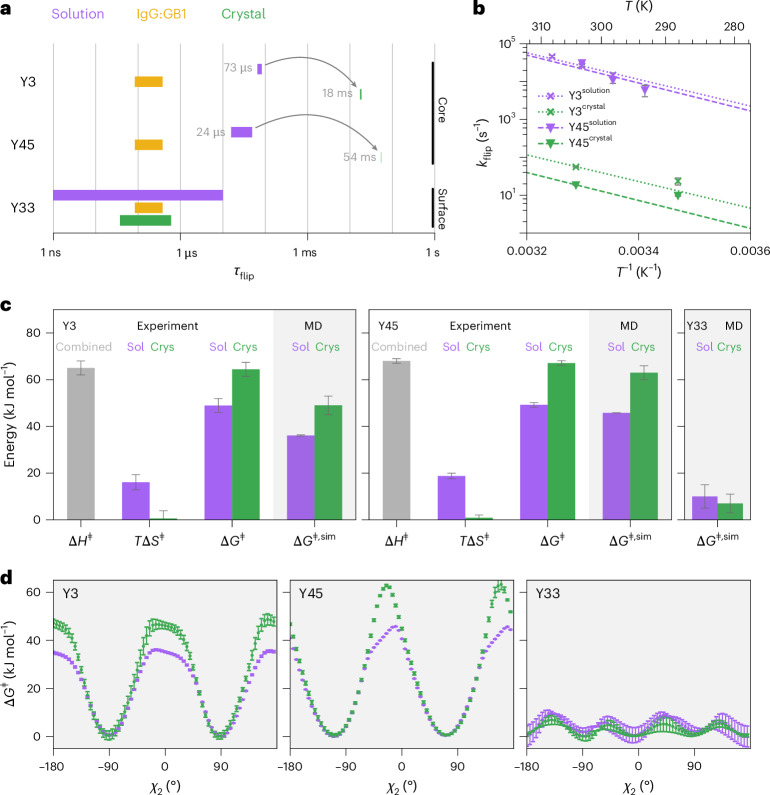
Thermodynamic parameters of tyrosine ring flips from experiment and simulations. **a**, Overview of the range of correlation time constants (including uncertainties) at 298 K (solution) or 304 K (crystal and complex). Values for Y33^crystal^ (green) and the three tyrosines in the IgG complex (yellow) were obtained from the detectors analysis of ^13^C spin relaxation; owing to lack of site-specific assignments, the maximum range of time constants that includes p1, p2 and p3 is shown here. Values for Y3^crystal^ and Y45^crystal^ were obtained from EXSY. Data for Y3^solution^ and Y45^solution^ come from ^13^C *R*_1*ρ*_ experiments^49^; the upper limit for Y33^solution^ flips is obtained from theoretical considerations regarding the limits of solution NMR relaxation dispersion measurements. **b**, Temperature dependence of *k*_flip_ of Y3 (crosses) and Y45 (rectangles) in solution^49^ (purple) and crystals (green), and the per-residue joint fit (see text). Errors of *k*_flip_ were determined by Monte Carlo error analysis as one standard deviation of 300 iterations based on one standard deviation of the spectral noise level. **c**, Fit results Δ*H*^‡^ and *T*Δ*S*^‡^ from **b** and corresponding activation free energies Δ*G*^‡^ at *T* = 298 K for Y3 and Y45 in solution (purple) and the crystal (green). Errors were determined by Monte Carlo error analysis as one standard deviation of 300 iterations based on the errors of *k*_flip_. $\Delta {G}^{\ddagger ,{\rm{sim}}}$ (grey background) are free energies from enhanced-sampling MD simulations (see **d**). Δ*H*^‡,solution^ = Δ*H*^‡,crystal^ (grey) owing to the fitting constraint. **d**, Δ*G*^‡^ profiles for ring flips of Y3, Y33 and Y45 obtained from enhanced-sampling MD simulations of GB1 in solution (purple) and the crystal (green). The asymmetry in some profiles is likely due to asymmetric environments of the rings or force field parameters. The error bars are determined by the variance of the free-energy differences measured by different walkers (see Methods for details).

The ring-flip correlation times *τ*_flip_ of the tyrosines in all three states of GB1_QDD_ are summarized in Fig. 5a. We sought to characterize properties of the transition state of the ring flips by studying the temperature dependence of *k*_flip_ of Y3 and Y45 (Fig. 5b). While the absolute rate constants in solution and crystals are very different, the slopes of as a function of 1/*T* in the crystal are similar, albeit slightly flatter in crystals than in solution, suggesting that Δ*H*^‡,solution^ ≥ Δ*H*^‡,crystal^ (Supplementary Note 2 and Supplementary Fig. 9). As the temperature range was limited for the MAS NMR measurements, we performed a joint Eyring fit of the rate constants from solution and crystal with the restriction that Δ*H*^‡,solution^ = Δ*H*^‡,crystal^ for each residue (Fig. 5b). Figure 5c shows the fitted parameters Δ*H*^‡^ and *T*Δ*S*^‡^ and the resulting Gibbs free energy of activation Δ*G*^‡^ at *T* = 298 K. We obtain positive values for Δ*H*^‡^ and Δ*S*^‡^ for both residues in solution and the crystal with a large difference in activation entropy, Δ*S*^‡,solution^ > Δ*S*^‡,crystal^. This observation confirms that the flips of Y3 and Y45 are slowed down because there is a reduced entropic gain for the ring flip in the crystal compared with solution. Comparing the activation free energies between solution and crystal results in for Y3 and (17.9 ± 1.5) kJ mol^−1^ for Y45, a value that is useful to benchmark simulations shown below.

Temperature-dependent *k*_flip_ of Y33^crystal^ determined with ^13^C *R*_1*ρ*_ spin relaxation and the detectors analysis did not show temperature dependence within experimental error. This is presumably due to the limited temperature range available for MAS measurements and the uncertainty of the detectors analysis. We therefore refrained from further analysing the temperature-dependent data for Y33.

### MD simulations coincide with experimental ring-flip kinetics

To gain mechanistic insights into the ring flips, we performed enhanced-sampling MD simulations^70^. MD simulations provide atomic-level information on protein dynamics in solution and crystals^31–36^ and are highly complementary to experimental methods. With increasing computational power, simulations of large crystal lattices, comprising multiple copies of the unit cell, and extending over the μs timescale, have become feasible^71^. However, the question at hand remains challenging, given that the ring flips occur on timescales spanning from tens of microseconds in solution to tens of milliseconds in the crystal. Such rare events are not amenable to current all-atom equilibrium MD simulations carried out on non-specialized computer architectures^72^.

Enhanced-sampling MD simulations^70^ allowed us to accelerate the exploration of the conformational space along the *χ*_2_ angle, utilizing the well-tempered metadynamics-extended adaptive biasing force (WTM-eABF) algorithm^73^ in its multiple-walker variant^74^, as implemented in NAMD^75^ and Colvars^76^ (see Methods for details). As shown in Fig. 5d, in both aqueous solution and crystal environments, our μs-timescale simulations show that the free-energy barriers against the ring flips of the buried Y3 and Y45 are substantially higher than that of the more exposed Y33. This result is in quantitative agreement with our experimental observations (Fig. 5c). Importantly, the absolute values of Δ*G*^‡^ for Y3 and Y45 agree well with those determined experimentally. The values for $\Delta \Delta {G}^{\ddagger ,{\rm{sim}}}$ are (13 ± 5) kJ mol^−1^ for Y3 and (17 ± 4) kJ mol^−1^ for Y45, virtually the same as the ones obtained experimentally ($\Delta \Delta {G}^{\ddagger ,\exp }=(15\pm 5)\,{{\rm{kJ\; mol}}}^{-1}$ for Y3 and (17.9 ± 1.5) kJ mol^−1^ for Y45). This good quantitative agreement of the free-energy barrier heights between experiments and MD simulations provides a solid foundation for a mechanistic understanding of the ring dynamics.

### Ring dynamics involve a complex network of interactions

In search of mechanisms underlying the ring flips, we investigated potential temporal correlations between ring flips of Y45 (that is, *χ*_2_) and fluctuations in other structural parameters, such as distances and angles.

In the simulation of the GB1 monomer in solution, *χ*_2_ alternates between two well-defined states (approximately 50° and −130°, respectively). The conformation of the ring axis, observed via the *χ*_1_ angle, predominantly remains unperturbed and exhibits only rare, short-lived excursions of roughly 50° (Supplementary Fig. 21). There is no apparent temporal correlation between fluctuations in *χ*_1_ and *χ*_2_.

**Fig. 6:**
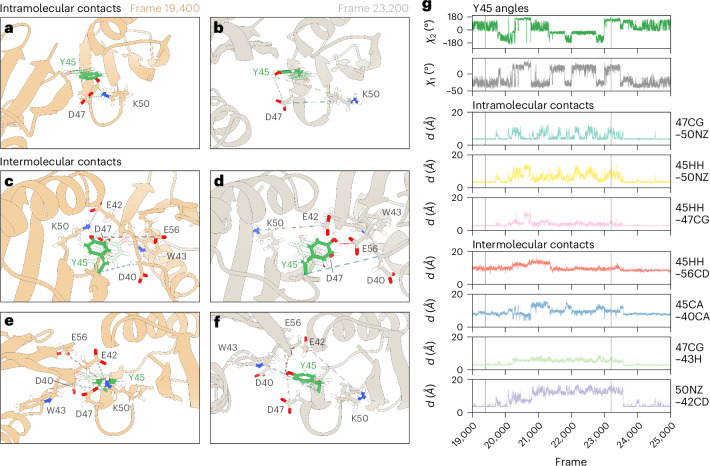
Structural insights into the interaction network of Y45 in crystals from MD simulations. **a**–**f**, Close-up of Y45 and residues interacting with it intramolecularly (**a**,**b**) or in a neighbouring molecule (**c**–**f**). Interactions are shown for two different frames of the simulations, representing a state in which Y45 is oriented towards the core of GB1 (frame 19,400; brown; **a**,**c**,**e**) or outward rotated (frame 23,200; grey; **b**,**d**,**f**). Pale and dark shades of brown and grey indicate chains A and B of the crystal, respectively. **g**, Time traces of the dihedral angles of the side chain of Y45, and relevant intra- and intermolecular distances. Vertical lines indicate the frames shown in **a**–**f**. The full time traces and data of the simulation of the monomer (in solution) are shown in Supplementary Figs. 21 and 22.

In the crystal lattice, *χ*_2_ not only fluctuates between two symmetry-equivalent positions separated by 180° but also populates intermediate states. Furthermore, excursions of the ring axis (from *χ*_1_ ≈ −25° to 20°) are longer-lived and often coincide with transitions in *χ*_2_ (Fig. 6g). In the two major *χ*_1_ states, the ring is rotated more or less ‘outwards’ (Fig. 6a,c,e and Fig. 6b,d,f, respectively). These states are associated with considerable rearrangements in the intramolecular interactions involving Y_45_^HH^, D_47_^CG^ and K_50_^NZ^. However, as these interactions are intramolecular, they may also occur in solution and thus cannot explain the observed deceleration of ring flips in the crystal.

We identified an additional set of intermolecular contacts, including residues D40, E42, W43 and E56 from a neighbouring protein subunit in the lattice (Fig. 6c–g). Fluctuations in the distances between these residues and the hydroxyl group of Y45—or its nearby interaction partners—tend to coincide with fluctuations in *χ*_1_ and/or *χ*_2_ of Y45. Since these contacts are necessarily absent in solution, we propose that they contribute to the increased free-energy barrier for ring flips observed in the crystal environment.

Overall, MD simulations indicate that a complex network of interactions modulates ring dynamics. We could not find indicators for a coupling between the ring flip and an expansion or contraction of the hydrophobic core (global ‘breathing’ motions; Supplementary Figs. 21–23). Instead, the ring dynamics (both flips and ring-axis reorientation) depend on a subtle interplay of intra- and intermolecular contacts.

## Discussion

Proteins function by sampling a spectrum of conformations. Consequently, they operate within a delicate balance of interactions, and their three-dimensional (3D) structures are typically only marginally stable. Even GB1_QDD_, considered a highly stable protein, has a free energy of unfolding (Δ*G*^0^ ≈ 20 kJ mol^−1^)^48^ that corresponds to the energy of just a few hydrogen bonds. It is, therefore, not surprising that additional interactions with the environment can profoundly reshape the protein’s free-energy landscape. In this study, we used aromatic ring flips as sensitive reporters of rare, cooperative motions to investigate how interactions between GB1 and either neighbouring molecules in the crystal or a large binding partner (the IgG antibody) influence its dynamics compared with the protein in solution.

We observed a spectrum of behaviours for the tyrosine ring flips in GB1. Y33, located on the protein surface, appears largely unaffected by its environment. It undergoes rapid ring flips on the nanosecond timescale in all three examined states. This finding aligns with a recent study of phenylalanine flips in three different crystal lattices of ubiquitin^77^.

In sharp contrast, the ring flips of Y3 and Y45 in GB1_QDD_ are slowed by approximately three orders of magnitude in the crystal. Our thermodynamic analysis reveals that this is due to a change of the entropic barrier between solution and crystal with 0 < Δ*S*^‡,crystal^ < Δ*S*^‡,solution^ (Fig. 5c). This entropic gain is counteracted by an enthalpic penalty, possibly due to a loosened transition state caused by breaking interactions, leading to the observed free-energy barriers Δ*G*^‡^. Δ*G*^‡,crystal^ is higher than in solution because Δ*S*^‡,crystal^ for going from the ring-flip-incompetent state to the ring-flip-competent states is smaller. Contacts with neighbouring molecules might reduce the capacity of GB1 to sample conformations that enable ring flips in the crystal (Fig. 1e). The network of contacts revealed in our MD simulations (Fig. 6c–f) may constitute such a framework where fewer microstates are accessible within a reduced configuration-space volume owing to defined interactions.

Surprisingly, in the IgG complex, the ring flips of Y3 and Y45 are accelerated compared with solution. We hypothesize that interactions between GB1_QDD_ and IgG stabilize conformations that favour ring flipping. This may be facilitated by the fact that GB1–IgG contacts occur primarily on the side of the protein opposite to Y3 and Y45 (Fig. 1f). The low-amplitude overall rocking motion of GB1 within the complex^63^, where contacts between GB1 and IgG are transiently formed and broken, might also contribute to the increased ring-flip rate constants. (Note that we imply this forming and breaking of transient contacts, but they have not actually been shown experimentally.)

The phenylalanine residues in the aromatic cluster (F30 and F52) show markedly different (much faster) ring-flip dynamics in the crystal compared with Y3 and Y45 (Fig. 4a). This observation directly shows that in crystals, the mechanisms enabling F30 and F52 flips must differ from those governing tyrosine rotations, and that, therefore, ring flips in crystalline GB1_QDD_ cannot be described by a global ‘breathing motion’.

In solution, by contrast, all aromatics in the core (Y3, Y45, F30 and F52) flip with a similar rate constant in the range of a few tens of thousands per second. One could assume that aromatic ring flips in solution are linked to collective ‘breathing motions’, while this motion is quenched in crystals. However, high-pressure NMR measurements imply that alternative pathways also exist in solution, allowing rings to flip via more local motions and rearrangements without an increase in the total protein volume at the transition state (Δ*V*^‡^)^50^. We argue that what is implied by the term ‘breathing motion’ is likely a complex rearrangement of local interactions, not necessarily involving an overall transient expansion.

Recent data support that ring-flip dynamics may not be governed by a single global mode: Lu et al. used fluorine-labelled tyrosines in conjunction with solution-state ^19^F NMR to study ring flips in GB1_T2Q_ in solution, crowded environments and inside cells^78^. As fluorocarbons are more hydrophobic than hydrocarbons, fluorinated tyrosine rings are expected to engage in stronger local interactions, resulting in higher energy barriers for ring flipping. Consistent with this, ring flips of fluorinated Y45 are slower than those of the non-fluorinated counterpart. Flips of fluorinated Y3 are much faster than those of fluorinated Y45, showing that Y3 and Y45 do not flip synchronously in F-Tyr-labelled GB1_T2Q_. While this behaviour may be due to the unique properties of fluorinated tyrosines, it underscores that timescales of aromatic ring flips are governed by multiple factors that likely involve a combination of local and collective motions.

Our data highlight the complexity of molecular mechanisms, such as protein–protein interactions, that modulate internal dynamics: intermolecular contacts can slow ring flips down (as seen for Y3 and Y45 in crystals), but they may also help stabilize states that facilitate ring flips, as evidenced by the accelerated flips in the IgG:GB1 complex. It is tempting to extrapolate our findings on GB1 to other crystalline systems. Since crystalline environments are inherently more densely packed than the protein’s solution environment, ring flips in many crystals are likely slower than in solution. However, the specifics may depend heavily on context, as demonstrated by our IgG:GB1 results. If intermolecular contacts favour conformations that facilitate ring flips, the crystalline environment might even accelerate them. Even the specific crystal lattice might manipulate ring-flip dynamics, just like overall rocking motion, which also depends on packing density^35^. Our findings may also have relevance for dynamics in the crowded cellular environment; in-cell NMR studies have shown that quinary interactions can promote partially unfolded states^79^, including the case of GB1^80,81^.

In conclusion, our study demonstrates that intermolecular contacts can profoundly influence the timescale of internal protein dynamics. While the precise impact of the crystal lattice or complex certainly depends on the specific protein, our findings underscore that the protein free-energy landscape is complex and highly dependent on the surroundings.

## Methods

### Sample preparation

#### Protein production and purification

GB1 was produced either (1) without isotope labelling for structure determination, (2) with uniform ^2^H, ^13^C and ^15^N labelling for backbone assignment, or (3) with uniform ^2^H and ^15^N and site-specific ^1^H–^13^C labelling at the (CH)^*ϵ*^-positions of Phe and Tyr residues for studying aromatic ring dynamics. The M9 minimal medium (M9) for isotopic labelling contained 5.25 g l^−1^ Na_2_HPO_4_, 3 g l^−1^ KH_2_PO_4_, 0.5 g l^−1^ NaCl, 1 g l^−1^ NH_4_Cl, 2 g l^−1^ D-glucose, 1 mM MgSO_4_, 0.1 mM CaCl_2_, 0.1 mM MnCl_2_, 0.05 mM ZnSO_4_, 0.1 mM FeCl_3_, 1 mg pyridoxine, 1 mg biotine, 1 mg D-pantothenic acid hemicalcium, 1 mg folic acid, 1 mg choline chloride, 1 mg niacinamide, 0.1 mg riboflavin and 5 mg thiamine hydrochloride.

Transformation of the plasmid containing the GB1 gene with the mutations T2Q, N8D and N37D^48^ into *Escherichia coli* BL21(DE3) was achieved by a standard heat shock protocol and plated on LB agar containing 100 μg ml^−1^ ampicillin (Amp). In the following, all shaking steps are performed at 37 °C and 200 rpm, all media formulations contain 100 μg ml^−1^ Amp, and cultures are inoculated such that the optical density at 600 nm (OD_600_) is 0.2 unless stated otherwise.

For (1), a single colony was used to inoculate an overnight culture of LB medium. The next day, fresh LB medium was inoculated and shaken until it reached an OD_600_ of 0.6 at which point expression was induced with 1 mM isopropyl-β-D-thiogalactopyranoside (IPTG). After shaking for 4 h, cells were collected by centrifugation for 15 min at 4 °C and 5,500 rcf. The pellet was either frozen at −20 °C or used for protein purification immediately.

For (2), the initial preculture in LB was followed by 3 precultures in M9 with 0%, 50% and 100% D_2_O over the course of 2 days to allow bacteria to adapt to the deuterated medium. The main culture in M9 medium, which was prepared with 100% D_2_O, underwent the same procedure as described for (1). Isotope labelling in the final preculture and the main culture was achieved using ^15^NH_4_Cl and D-^13^C_6_-^2^H_7_-glucose.

For (3), the same protocol as for (2) was followed up until the main culture except for using D-^2^H_7_-glucose. The main culture was shaken until it reached an OD_600_ of 0.6 at which point 100 mg l^−1^ sodium [3,3-^2^H_2_]([3,5-^13^C_2_; 2,4,6-^2^H_3_] phenyl) pyruvate and 50 mg l^−1^ sodium [3,3-^2^H_2_]([3,5-^13^C_2_; 2,6-^2^H_2_] 4-hydroxyphenyl) pyruvate^47^ were added. After shaking for 1 h, expression was induced with 1 mM IPTG. The culture was shaken at 20 °C and 200 rpm overnight and collected the next morning as described above.

The cell pellet was resuspended in 10 ml purification buffer (10 mM Tris, 1 mM ethylenediaminetetraacetic acid, pH 7.5) and lysis was achieved by a heat shock at 80 °C for 10 min. After 5 min on ice, the suspension was centrifuged for 40 min at 4 °C and 47,850 rcf. The supernatant was loaded onto a RESOURCE Q column (Cytiva) and eluted in purification buffer with a gradient of 0–1 M NaCl in 10 column volumes. The fractions containing GB1 were concentrated with an Amicon Ultra Centrifugal filter with a 3 kDa molecular weight cut-off and size exclusion chromatography was performed on a HiLoad 16/600 Superdex 75 pg column (Cytiva) in crystallization buffer (50 mM sodium phosphate, pH 6.5).

#### Crystallization

Crystallization was achieved by dialysis^83^ in a self-made dialysis button consisting of the upper part of a microtube and a dialysis membrane with a 3.5 kDa molecular weight cut-off (SnakeSkin) (Supplementary Fig. 1a). The protein in crystallization buffer was concentrated to 30 mg ml^−1^ and 120 μl was loaded into the cap of the microtube. The dialysis button was closed with the dialysis membrane and placed into 10 ml of reservoir solution (50% 2-methyl-2,4-pentanediol (MPD), 25% isopropanol) (Supplementary Fig. 1b). Dialysis was performed under constant agitation at 4 °C. Microcrystals started to grow after 1–2 days (Supplementary Fig. 1c).

For NMR samples, the microcrystals were filled into a 1.3 mm (approximately 3 mg) or 1.9 mm (approximately 12 mg) (Supplementary Table 2) MAS rotor (Bruker) by centrifugation (10 min at 4,000 rcf) after 7 days.

To obtain crystals for single-crystal XRD, dialysis was performed for a total duration of 2 weeks, after which large rod-shaped crystals appeared.

For the success of crystallization, avoiding Cl^−^ ions in the crystallization buffer is crucial^51^. We also found that using an ultracentrifuge to transfer the crystals into the NMR rotor often leads to polymorphic samples. Although we did not investigate this systematically, we suspect that the vacuum in this kind of centrifuge leads to increased isopropanol evaporation, and we achieved more consistent results by using a tabletop centrifuge.

#### IgG:GB1 complex

The sample of the IgG:GB1 complex was prepared as described previously^53^. In short, lyophilized IgG from human serum (Sigma-Aldrich I4506) was dissolved in 50 mM sodium phosphate (pH 6.5) to a concentration of 0.15 mM. The antibody was mixed with 0.3 mM GB1 in the same buffer in a volume ratio of 1:1. The complex precipitates upon mixing and was filled into an MAS NMR rotor by centrifugation (overnight at 68,000 rcf).

### Structure determination

For cryogenic structure determination, crystals were collected at 4 °C, mounted on a MiTeGen cryoloop and cooled to 100 K in liquid nitrogen without any additional cryoprotectant. Data collection was performed on the BM07-FIP2 beamline at the European Synchrotron Radiation Facility (ESRF) at 100 K. Two datasets, each comprising 1,800 images, were recorded with 0.2° rotation and 0.1 s exposure per image. The images were collected at an energy of 12.657 keV from 2 different positions on the same crystal (Supplementary Fig. 2a).

For room-temperature structure determination, the crystal was mounted on a MiTeGen crystallization loop directly on the diffractometer at beamline ID30B of the ESRF (Supplementary Fig. 2b). Data were collected at 295 K using an HC-Lab humidity control device (Arinax), with the relative humidity maintained at 99%, which was experimentally determined to be optimal for preventing crystal dehydration. A single dataset comprising 1,800 images was recorded with an oscillation range of 0.2° and an exposure time of 0.01 s per image. Data were collected at an X-ray energy of 13 keV.

All diffraction frames were processed using autoPROC^84^, data were integrated in XDS^85^, and the integrated intensities were scaled and merged in AIMLESS and POINTLESS, as implemented in CCP4^86^. AlphaFold2^87^ was used to predict an initial model, which was used for molecular replacement with PHASER^88^. The model was optimized through iterative rounds of model building in COOT^89^ and refinement in PHENIX.REFINE^90^. At all stages of the refinement, translation–libration–screw-rotation was applied. Data collection and refinement statistics are shown in Supplementary Table 1. The cryogenic and room-temperature structures and associated structure factor amplitudes were deposited in the Protein Data Bank (PDB) after validation with MolProbity^91^ under the PDB codes 9I2I and 9T8Z, respectively.

### Powder diffraction on microcrystals

Samples for powder XRD were prepared as for the NMR experiments and placed inside borosilicate capillaries with a 1 mm outer diameter, a 0.01 mm wall thickness and an 80 mm length (Hilgenberg). The samples were measured on a XEUSS 3.0 HR (Xenocs SAS) in <0.1 mbar vacuum using radiation from a microfocus Cu source collimated with a 3D multilayer mirror and shaped by scatterless slits, and an Eiger2 1M pixel array detector. Several sample-detector distances and collimation settings were tested for optimal data quality. A total of 36 frames of 120 s exposure each from multiple places in a capillary were cumulated to achieve good orientation statistics. The frames were cumulated and reduced in the XSACT suite^92^. No background or buffer subtraction was done since all other scatterers (capillary, buffer) showed featureless patterns.

Analysis of the data was done using GSASII^93^. We refined the unit cell parameters and the sample displacement following the approach of Le Bail for the peak intensities^94^. An angle-independent Gaussian and a domain size-related Lorentzian term were used to fit the peak shapes and a polynomial background.

All peaks could be indexed with the *C*121 phase also observed in single-crystal diffraction; no other phase was detectable. Refinement of the parameters resulted in *a* = 77.9 Å, *b* = 35.3 Å, *c* = 51.4 Å and *β* = 122.3°, very close to the values obtained by single-crystal XRD (Supplementary Table 1).

### NMR spectroscopy

MAS NMR experiments were recorded on a Bruker Avance Neo spectrometer operating at 16.44 T (700 MHz ^1^H Larmor frequency) with triple-channel (^1^H, ^13^C and ^15^N) probe heads for 1.3 mm or 1.9 mm MAS rotors. All measurements were performed with ^1^H detection and cross-polarization-based transfers. The following sequences used in this work are implemented in the ssNMRlib library^95^: 2D hNH, 3D hCONH, 3D hCOcaNH, 3D hCANH, 3D hCAcoNH, 3D hcaCBcaNH, 3D hcaCBcacoNH, 3D HhNH radio frequency-driven recoupling, pseudo-3D hNH *R*_1_ and pseudo-3D hNH *R*_1*ρ*_. Experiments used to study the aromatic ^1^H–^13^C signals of specifically (CH)^*ϵ*^ labelled Phe and Tyr residues are shown in Supplementary Fig. 4 (not currently implemented in NMRlib). They all contain a ^13^C EBURP2 pulse^96^ to selectively excite the aromatic C^*ϵ*^ signal and suppress the natural-abundance carbon background that results particularly from cross-polarization from amide hydrogens (which spectrally overlap with those of aromatic hydrogens) and C^*α*^ or C′ carbons. In addition, the water suppression element was moved in front of the ^13^C chemical-shift evolution period *t*_1_ to avoid cross-peaks between C^*ϵ*1^ and C^*ϵ*2^ that may arise from exchange dynamics or magnetization transfer (proton-driven spin diffusion). In all cases, the water-suppression element consisted of a composite-pulse decoupling sequence with constant amplitude and a pseudo-random variation of the pulse durations and phases. The determination of the sample temperature was performed on an external sample (1.3 mm or 1.9 mm rotor) containing lyophilized protein rehydrated with a solution of 25 mM TmDOTP and 10 mM 2,2-dimethyl-2-silapentane-5-sulfonate sodium salt (DSS) in 80% D_2_O for each measurement condition^97^. The experimental conditions are summarized in Supplementary Table 2.

#### Assignment experiments

Six 3D spectra (hCONH, hCOcaNH, hCANH, hCAcoNH, hcaCBcaNH and hcaCBcacoNH) were recorded to assign the resonances of H^N^, N^H^, C′, C^*α*^ and C^*β*^ atoms of GB1 in the crystal.

A 3D HhNH experiment with ^1^H–^1^H radio frequency-driven recoupling was used to assign aromatic (H^*ϵ*^) protons of (CH)^*ϵ*^-labelled Phe and Tyr by probing their proximity to amide (H^N^) protons.

A 2D HcH EXSY experiment was recorded to probe potential exchange between ^13^C^*ϵ*^ atoms in tyrosines in the IgG:GB1 complex (Supplementary Fig. 4b). The exchange delay *τ* of 256 ms was applied while magnetization was stored on ^13^C nuclei after the indirect ^1^H evolution period.

#### Experiments to probe dynamics

Experiments to probe amide ^15^N and aromatic ^13^C longitudinal *R*_1_ and rotating frame relaxation *R*_1*ρ*_ were performed as pseudo-3D spectra. The pseudo dimension was used to vary the relaxation delay *τ* or the duration of the spinlock pulse for *R*_1_ and *R*_1*ρ*_, respectively (Supplementary Fig. 4c,d). The delays and pulse lengths used can be found in Supplementary Figs. 11 and 17–19.

EXSY experiments were performed to study the timescale of tyrosine ring flips for which separate signals for H^*ϵ*1^–C^*ϵ*1^ (site a) and H^*ϵ*2^–C^*ϵ*2^ (site b) could be observed (Supplementary Fig. 4e). If a ring flips during the longitudinal C^*z*^ mixing time *τ*, sites a and b exchange, resulting in the cross-peaks H^*ϵ*2^–C^*ϵ*1^ (ab) and H^*ϵ*1^–C^*ϵ*2^ (ba) in the final spectrum. The experiment was implemented as a pseudo-4D experiment with two frequency dimensions (^1^H and ^13^C), and two dimensions related to the EXSY: along one dimension, a variable exchange delay *τ* allowed monitoring the build-up (owing to the exchange process) and subsequent decay (owing to *R*_1_ relaxation, that is, incoherent processes, and possibly spin diffusion, that is, coherent transfer of magnetization to other nuclei) of cross-peak intensities. In the fourth dimension, the position of the exchange delay *τ* was alternated, to be either before or after the ^13^C chemical-shift evolution period *t*_1_. In the former case, only diagonal peaks are observed, and their intensity decays over *τ*. In the latter case, the build-up of exchange cross-peaks is observed, and the intensities of diagonal and cross-peaks are subject to the same decay as in the reference experiment. When subtracting the two spectra, diagonal peaks are eliminated, facilitating the quantification of cross-peaks. As part of the exchange delay, an additional water suppression element with a constant duration was implemented, making the minimum possible *τ* 10 ms. We verified whether the observed cross-peaks may result from spin diffusion rather than from ring flips, by repeating the experiments at different MAS frequencies *ν*_r_ (30 kHz and 39 kHz) while keeping the sample temperature constant. Spin diffusion, but not ring-flip dynamics, is expected to be MAS frequency dependent.

REDOR experiments were performed with a shift Δ of the first inversion pulse in each rotor period *τ*_r_ to scale down the dipolar coupling and allow better sampling^98,99^ (Supplementary Fig. 4f). The dephasing curve was sampled at time points 2*τ*_r_ + (*n* − 1)2*τ*_r_ with *n* = 1−15. The incrementation of rotor periods was implemented as a third dimension. The REDOR block was followed by a short *z*-filter to suppress unwanted coherences that might have built up during the recoupling period. The reference experiment in which the inversion pulses on the ^1^H channel were replaced with equally long delays was sampled at *n* = 1, 7, 13, and the missing reference intensities were obtained from a linear fit of these points. This approach allows spending more time on collecting the more informative data points of the recoupling experiment because the decay in such highly deuterated samples is slow and sensitivity high, such that the fit of the data is very robust.

### NMR data analysis

All spectra were processed with Bruker Topspin 4.1.4 and then converted to UCSF format with the bruk2ucsf programme provided in Sparky^100^. The analysis of the experiments to investigate dynamics was done with scripts using the Nmrglue package for Python^101^. All spectra were indirectly referenced to DSS via MPD^69^.

#### Backbone assignment

Non-uniform sampling reconstruction and processing of backbone assignment spectra was performed in Topspin before conversion to UCSF format. Peaks were picked in CcpNmr Analysis Version 3^102^ and exported for automated resonance assignment by FLYA (CYANA v3.98.15)^103^. Chemical-shift tolerances were set to 0.1 ppm for ^1^H and 0.4 ppm for ^13^C and ^15^N resonances. Minor manual corrections to the automated assignment were made in CcpNmr. Representative sections of the 3D spectra are shown in Supplementary Fig. 5. The completeness of the assignment is visualized in Supplementary Fig. 6, and all assigned chemical shifts can be found in Supplementary Table 3. The full assignment is published under the BMRB access code 53330.

#### Determination of ring-flip rates from EXSY experiments

The pseudo-4D spectrum was processed as individual 2D planes resulting in a reference and an exchange spectrum for each exchange time. Peak intensities were obtained by fitting a 2D Gaussian lineshape with functions provided in the Nmrglue package. Intensities of the diagonal and cross-peaks were extracted in the reference and difference spectrum respectively. For residue Y45, the four signals (A, B, AB and BA) were fitted as described in Farrow et al. (1994)^66^ to obtain the rate of the ring flip *k*_flip_. The longitudinal relaxation rates *R*_1,A_ and *R*_1,B_ were set to the determined values from the relaxation experiment, and the populations were set to *p*_A_ = *p*_B_ = 0.5. Owing to substantial signal overlap of Y3 and Y33, we extracted *k*_flip_ of residue Y3 only from the build-up of the cross-peak BA intensities *I* according to $I(t)=I(0)(1-\exp [-2{k}_{{\rm{flip}}}t])\exp [-{R}_{1,{\rm{B}}}t]$. The fitted curves are shown in Supplementary Fig. 13. Errors were determined by Monte Carlo analysis. Five hundred noisy datasets were generated using the spectral noise level defined as the standard deviation of intensities across a spectral region without any signal. The error of each fit parameter was estimated as the standard deviation of that given parameter across the fits of these synthetic datasets.

#### Determination of relaxation rate constants

Peak intensities were extracted from a series of 2D planes with different relaxation delays (*R*_1_) or spinlock pulse length (*R*_1*ρ*_) as described above. Relaxation rate constants were obtained by an exponential fit of the intensities. Error analysis was performed as described above. All fits are shown in Supplementary Figs. 17–19.

#### Detectors

Relaxation rates were analysed using the detectors approach as implemented in the package Detectorist^67^. Sensitivities were calculated for each of the relaxation rates measured. For the site-selective ^13^C–^1^H Tyrs, the sensitivity was calculated assuming a ^13^C–^1^H dipolar coupling at a distance of 1.09 Å, and a ^13^C CSA tensor with principal components 180 ppm, 143 ppm and 27 ppm (ref. ^104^). For the backbone ^15^N sites, the sensitivity was calculated assuming the directly bonded ^1^H at a distance of 1.02 Å, and principal components of the ^15^N CSA tensor of 229.2 ppm, 78.6 ppm and 52.3 ppm (ref. ^105^). Each sensitivity was digitized using 200 time points, logarithmically spaced between 100 fs rad^−1^ and 1 ms rad^−1^. It should be noted that owing to the form of the spectral density, the correlation times involved are naturally in units of seconds per radian. For comparison with results from EXSY, these have been explicitly converted into the base unit of seconds.

Detector optimization was performed using the singular value decomposition method^106^. Specifically, a matrix, ***M***, was produced whereby each of the *N* rows represents the sensitivity of a corresponding relaxation rate. The singular value decomposition of this matrix was taken:

1 $${\boldsymbol{M}}={\boldsymbol{U}}{\boldsymbol{\Sigma }}{{\boldsymbol{V}}}^{{\prime} }.$$

The singular values (diagonal entries in **Σ**) were sorted, the matrices truncated to include *k* singular values (*k* was varied from 2 to 7 for model selection). Linear programming was performed to optimize linear combinations of the orthogonal singular vectors in the truncated ${{\boldsymbol{V}}}_{k}^{{\prime} }$ to identify well-formed detectors. The coefficients of ${{\boldsymbol{V}}}_{k}^{{\prime} }$ giving rise to detectors then form the rows of the transformation matrix ***Q***, which can be used to determine the matrix ***r***, containing ‘detector vectors’:

2 $${\boldsymbol{r}}={{\boldsymbol{U}}}_{k}{{\boldsymbol{\Sigma }}}_{k}{{\boldsymbol{Q}}}^{-1},$$

for which each of the *N* columns, ***r***_*i*_, gives a linear combination of ≤*k* detectors giving the corresponding relaxation rate. The detector responses may then be calculated using a non-negative least squares to solve for the detector responses, ***ρ***:

3 $${\boldsymbol{\rho }}={\left[\begin{array}{c}{{\boldsymbol{r}}}_{1}/{e}_{1}\\ \vdots \\ {{\boldsymbol{r}}}_{N}/{e}_{N}\end{array}\right]}^{-1}\frac{{\boldsymbol{R}}}{{\boldsymbol{e}}},$$

where ***R*** and ***e*** are vectors containing the experimental relaxation rates and uncertainties, respectively. The detector sensitivities are obtainable directly from the product ${\boldsymbol{Q}}{{\boldsymbol{V}}}_{k}^{{\prime} }$. The number of singular values to be included in the analysis was identified by performing the analysis for *k* = 2–7, back-calculating the relaxation rates, then choosing the number of singular values that gave the lowest median reduced chisq value.

#### Determination of dipolar order parameters from REDOR experiments

Peak intensities were extracted as described above. The reference curve was linearly interpolated to obtain intensities *S*_0_ at the same time points as in the dephasing experiment *S*_rec_. The REDOR curve was calculated as Δ*S*/*S*_0_ = (*S*_0_ − *S*_rec_)/*S*_0_. The observed dipolar coupling strength ${\delta }_{{\rm{D}}}^{{\rm{obs}}}$ and tensor asymmetry *η* were determined by a 2D grid search against simulated curves. The simulated curves with varying *δ*_D_ (0–50,000 Hz with a step size of 50 Hz) and *η* (0–1 with a step size of 0.05) were generated using the GAMMA simulation package for numerical spin dynamics simulations^107^. Chi-square minimization between the simulated and experimental curves was used to find the best-fit values for *δ* and *η*. Error determination was performed as described above. Dipolar order parameters *S* were calculated with $S=({\delta }_{{\rm{D}}}^{{\rm{obs}}}/{\delta }_{{\rm{D}}}^{{\rm{rigid}}})$ with ${\delta }_{{\rm{D}}}^{\mathrm{rigid}}$ = 46,656 Hz, corresponding to a ^1^H–^13^C distance of 1.09 Å. The fits are shown in Supplementary Figs. 14 and 15.

### Computational details

#### Computational assays

Two distinct computational assays were constructed for GB1 in its crystal form and in an aqueous solution, using the visualization package VMD 1.9.4^108^, and the CHARMM-GUI utility^109^. The crystal assay consisted of 8 proteins and 2,456 water molecules. The aqueous solution consisted of a single protein immersed in a bath of 6,384 water molecules. To ensure electric neutrality of each assay and mimic physiological conditions, NaCl was added at a concentration of 150 mM. After suitable thermalization, the dimensions of computational assays were approximately *a* = 80.2 Å, *b* = 36.4 Å and *c* = 53.2 Å, with *α* = 90.0°, *β* = 120.7° and *γ* = 90.0°, for the crystal form, and *a* = 58.7 Å, *b* = 58.7 Å and *c* = 58.7 Å, for the aqueous solution, corresponding to 16,704 and 26,432 atoms, respectively.

#### MD simulations

All MD simulations were carried out using NAMD 3.0^75^, with the AMBER ff19SB force field for proteins and the OPC water model^110^. Periodic boundary conditions were applied for all computational systems. The temperature was maintained at 298 K by means of a stochastic velocity rescaling thermostat^111^ with a time parameter of 1 ps^−1^ for coupling, and pressure at 1 bar, using the Langevin piston algorithm^112^. All covalent bonds between heavy and hydrogen atoms were constrained to their equilibrium length with the Rattle algorithm^113^. Water molecules were constrained to their equilibrium geometry with the Settle algorithm^114^. Long-range electrostatic forces were computed with the particle-mesh Ewald algorithm^115^ and a grid spacing of 1.2 Å. Short-range van der Waals and electrostatic interactions were smoothly truncated with a 10 Å spherical cut-off. Hydrogen mass repartitioning was applied to the protein and surrounding lipids to allow longer integration time steps to be utilized^116^. The Verlet-I/r-RESPA multiple time-stepping algorithm was used to integrate the equations of motion with an effective time step of 4 fs for short-range interactions and 8 fs for long-range interactions^117^.

#### Free-energy calculations

As a preamble to the free-energy calculations, the two computational assays underwent a suitable energy minimization, followed by 400 ns of thermalization bereft of geometrical restraints. The potentials of mean force (PMFs), Δ*G*(*χ*_2_), underlying the isomerization of the side chain of residues Y3, Y33 and Y45 about the *χ*_2_ torsional angle, in the crystal form of the protein and in the aqueous solution, were determined with the well-tempered metadynamics extended adaptive biasing force (WTM-eABF) algorithm^73,118^. In a nutshell, it relies upon the integration of the average force acting on *χ*_2_, obtained from unconstrained MD simulations. In the course of the simulation, a biasing force is estimated such that, once applied to the system, it yields a Hamiltonian devoid of an average force exerted along *χ*_2_. As a result, all values of *χ*_2_ are sampled with an equal probability, which, in turn, greatly improves the accuracy of the calculated free energies. The transition pathway spanned 360°, that is, −180° ≤ *χ*_2_ ≤ + 180°, and was discretized in bins 5° wide, where samples of the local force acting along *χ*_2_ were accrued. To mitigate the risk of deleterious non-equilibrium effects, no time-dependent bias was applied until a threshold of 10,000 samples was reached^119^. In consideration of the substantial isomerization timescales measured experimentally, likely to be related to slowly relaxing degrees of freedom coupled to *χ*_2_, likely to hamper convergence of the free-energy calculation, a multiple-walker strategy was used to improve ergodic sampling^74^, with up to four walkers. The statistical error associated to the PMFs was estimated from the variance of the free-energy differences measured by each walker. Analysis of the trajectories was performed using VMD 1.9.4^75^.

## Online content

Any methods, additional references, Nature Portfolio reporting summaries, source data, extended data, supplementary information, acknowledgements, peer review information; details of author contributions and competing interests; and statements of data and code availability are available at 10.1038/s41557-026-02155-0.

## Supplementary information

Supplementary Information Supplementary Notes 1 and 2, Figs. 1–23 and Tables 1–3.

Peer Review File

## Data Availability

The cryo and room-temperature crystal structures of GB1_QDD_ are deposited at the PDB under the access codes 9I2I and 9T8Z, respectively. The solid-state NMR backbone assignment of GB1_QDD_ is deposited at the BMRB under the access code 53330. NMR spectra, analysis scripts and raw data are publicly available at the ISTA research explorer (10.15479/AT-ISTA-20641)^120^. Files to reproduce the enhanced-sampling MD simulations are publicly available at the ISTA research explorer (10.15479/AT-ISTA-21145)^121^.
