# Supplementary material for: Aromatic ring flips reveal reshaping of protein dynamics in crystals and complexes
Source: Nat Chem. 2026 Jun 10;18(7):1221–30. doi: 10.1038/s41557-026-02155-0 (PMC13323056; doi:10.1038/s41557-026-02155-0)
Supplement: Supplementary file 1 — Supplementary Notes 1 and 2, Figs. 1–23 and Tables 1–3. [file 41557_2026_2155_MOESM1_ESM.pdf]

nature chemistry

Supplementary information

https://doi.org/10.1038/s41557-026-02155-0

Aromatic ring flips reveal reshaping of protein dynamics in crystals and complexes

In the format provided by the authors and unedited

Contents

Supplementary note 1: Details about the analysis of dipolar order parameters and relaxation rates **1**

Supplementary note 2: Considerations about dependency of rate constants on $\Delta H^{\ddagger}$ and $\Delta S^{\ddagger}$ **2**

Supplementary figures and tables **3**

Supplementary note 1: Details about the analysis of dipolar order parameters and relaxation rates

Dipolar order parameters

The $^{1}H-^{13}C$ dipolar order parameter S (defined as $S = (\delta_D^{obs}/\delta_D^{rigid})$, where δ_D^{obs} is the measured dipolar-coupling tensor anisotropy and δ_D^{rigid} is the dipolar-coupling tensor anisotropy in the absence of motion), ranges from 1 for totally rigid sites to 0 for flexible disordered sites. We determined δ_D^{obs} using Rotational-Echo DOuble-Resonance (REDOR) experiments (see methods section for details). The order parameter reports on amplitudes of motion on timescales up to hundreds of µs (*1*). Faster motions do not result in an averaging of the dipolar coupling that is observable with this method.

Gauto et al. modeled the motional averaging due to a ring flip with simulations, and determined a theoretical value of $S = 0.625$ (*2*); reductions below this value point to additional motions, such as the libration of the ring within each rotamer state, motion of the backbone, or the ring axis (i.e., the χ_1 angle).

As discussed in the main text, the dipolar order parameters of Y3 and Y45 in the crystal are approximately 0.8. This is due to the slow ring flips that do not lead to a reduction of the δ_D^{obs} and therefore the dipolar order parameter. For Y33 and the phenylalanine, we determined order parameters lower than the expected value for a ring flip, which indicates that these rings flip faster than roughly one hundred µs and that there must be additional motion of the side chain.

Relaxation rate analysis with detectors and estimation of ring flip timescales

In the main text, we talk about the determination of upper and lower bounds of the ring-flip rate constants for Y33^{crystal}, p1, p2, and p3. Here, we provide a more detailed explanation of this analysis.

The detectors approach (*3, 4*) uses relaxation rate constants, in our case ^{13}C R_1 (sensitive to motions on the hundreds of picoseconds to tens of nanoseconds time scale) and $R_{1\rho}$ (hundreds of ns to hundreds of µs), to determine the amount of motion in certain time windows, the so-called detector sensitivities $\rho_i(\tau)$. The expected response of a dynamic process that occurs on a timescale corresponding to the maximum of a detector sensitivity is $(1 - S^2)$.

The responses that we obtain are clearly lower than the expected value for a ring flip, which is $(1-S^2) = 0.609$ (Figure 4B, lower left panel). Due to the nature of our experiments, there is a blind spot between $\rho_1(\tau)$ and $\rho_2(\tau)$ (Figure 4B, blue rectangle); motion in this window does not lead to sizeable relaxation in any of the experiments. We know from the order parameters that Y33^{crystal}, p1, p2, and p3 flip faster than hundreds of µs and assume that rings do not flip faster than hundreds of nanoseconds. We therefore conclude that these tyrosines flip on timescales that are within the blind spot.

To further limit the range, we scaled $\rho_1(\tau)$ and $\rho_2(\tau)$ by $(1 - S^2) = 0.609$. We determined the correlation times that correspond to the intersection of the scaled sensitivities and the responses ρ_1 and ρ_2 for

Y33^{crystal}, p1, p2, and p3 (Figure 4B, lower right panel, S8). The so-determined correlation times result in the upper and lower bounds of the ring-flip rate constants for Y33^{crystal} and the tyrosines in the IgG:GB1 complex.

Supplementary note 2: Considerations about dependency of rate constants on $\Delta H^\ddagger$ and $\Delta S^\ddagger$

In the main text we argued that the flatter dependency of the rate constants as a function of the inverse temperature, $\ln(k_{\text{flip}}) = f(1/T)$, in the crystal compared to solution (Fig. 5B) suggests that $\Delta H^{\ddagger,\text{solution}} \geq \Delta H^{\ddagger,\text{crystal}}$.
Here, we show that this conclusion follows simply from the Eyring equation, which is given by:

$$k = \frac{k_B T}{h} \exp(\frac{\Delta S^\ddagger}{R}) \exp(\frac{-\Delta H^\ddagger}{RT}) \tag{1}$$

which can be rearranged as follows:

$$\begin{aligned}
\ln k &= \ln\left(\frac{k_B T}{h}\right) + \frac{\Delta S^\ddagger}{R} - \frac{\Delta H^\ddagger}{RT} \\
&= \ln k_B + \ln T - \ln h + \frac{\Delta S^\ddagger}{R} - \frac{\Delta H^\ddagger}{RT} \\
&= \left(\ln k_B - \ln h + \frac{\Delta S^\ddagger}{R}\right) + \ln T - \frac{\Delta H^\ddagger}{RT} \\
&= \left(\ln k_B - \ln h + \frac{\Delta S^\ddagger}{R}\right) + \ln T - \frac{\Delta H^\ddagger}{R} \cdot \frac{1}{T}
\end{aligned} \tag{2}$$

With $c = \left(\ln k_B - \ln h + \frac{\Delta S^\ddagger}{R}\right)$, we obtain an Arrhenius-like representation:

$$\ln k = (c + \ln T) - \frac{\Delta H^\ddagger}{R} \cdot \frac{1}{T} \tag{3}$$

Consequently, the slope of the rate constant (plotted on a logarithmic scale) as a function of $1/T$ is approximately proportional to $\Delta H^\ddagger$. (The temperature dependence of the first term is small over a relevant temperature range and with the values of $\Delta H^\ddagger$ and $\Delta S^\ddagger$ assumed here.) An identical slope would mean that $\Delta H^{\ddagger,\text{solution}} = \Delta H^{\ddagger,\text{crystal}}$. The large vertical offset of k_{flip} must then be due to differences in the activation entropy $\Delta S^{\ddagger,\text{solution}} > \Delta S^{\ddagger,\text{crystal}}$.
For our analysis, we performed a residue-wise fit of rate constants measured in solution and the crystal with $\Delta H^{\ddagger,\text{solution}} = \Delta H^{\ddagger,\text{crystal}}$. The joint fit with a restraint is useful to avoid over-fitting, as the temperature range was limited for the MAS NMR measurements (by the available cooling power against the MAS-induced heating and sample stability). We also tested a fit imposing $\Delta H^{\ddagger,\text{solution}} > \Delta H^{\ddagger,\text{crystal}}$, which results in a very similar overall conclusion, namely that $\Delta S^{\ddagger,\text{solution}} > \Delta S^{\ddagger,\text{crystal}}$.
Figure S9 shows some exemplary cases for different combinations of $\Delta H^\ddagger$ and $\Delta S^\ddagger$.

Supplementary figures and tables

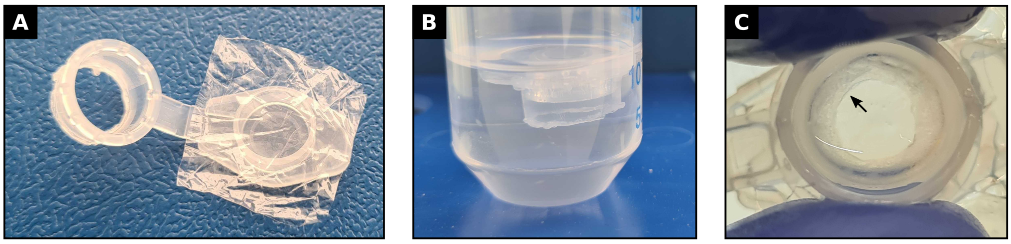

Figure S1: **A** Dialysis button made from the upper part of a microtube and dialysis membrane. **B** Dialysis button in reservoir solution. **C** Dialysis button with microcrystals forming a ring on the dialysis membrane.

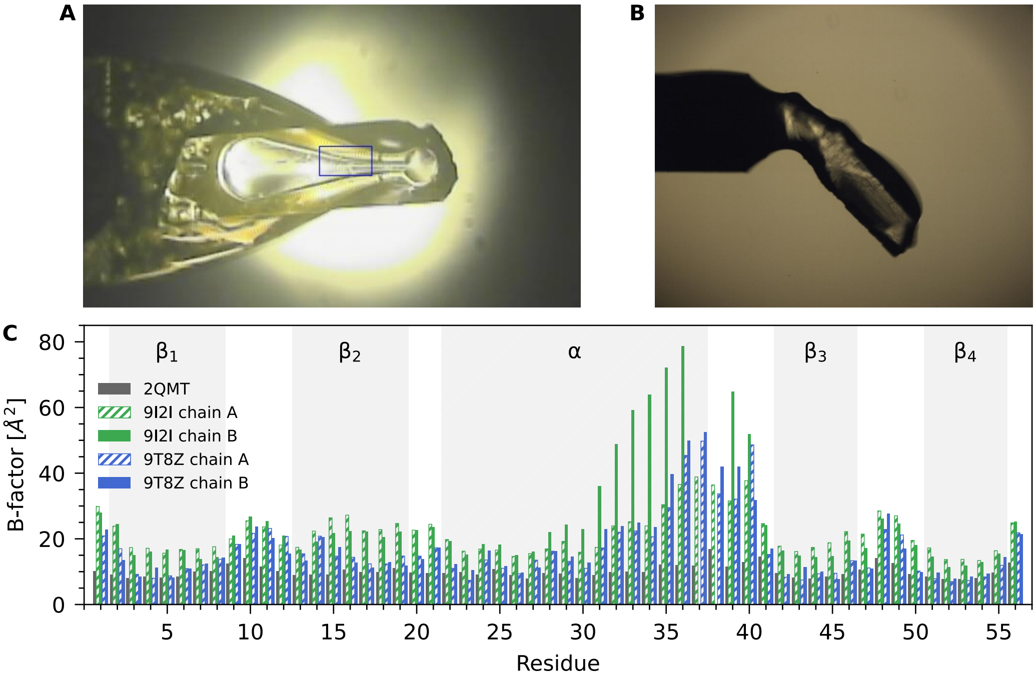

Figure S2: (A) Crystal of GB1 (800x350x200 µm^3) mounted on a crystallization loop under cryogenic condition at 100 K. (B) Crystal of GB1 (300x150x150 µm^3) mounted on a crystallization loop under 99% relative humidity at 295 K. (C) B-factors of GB1$_{T2Q}$ (gray, PDB 2QMT), GB1$_{QDD}$ at 100 K (green, PDB 9I2I), and GB1$_{QDD}$ at 295 K (blue, PDB 9T8Z) crystal structures. Normalized B-factors for GB1$_{T2Q}$ and GB1$_{QDD}$ at 100 K are shown in Figure 2B.

Table S1: X-ray data collection and refinement statistics.

	GB1 - 100 K	GB1 - 295 K
**Data collection**		
Beamline	BM07-FIP2 (ESRF)	ID30B (ESRF)
Space group	$C121$	$C121$
Cell dimensions		
a, b, c (Å)	77.48, 35.18, 49.94	78.70, 35.41, 51.82
β (°)	122.51	122.60
Resolution (Å)	42.11 – 1.08 (1.21 – 1.08) *	31.23 – 1.69 (1.73 – 1.69) *
R_{pim}	0.012 (0.259)	0.055 (0.214)
$I/\sigma I$	22.6 (2.0)	9.1 (4.7)
Completeness ellipsoidal (%)	86.5 (45.3)	97.1 (86.8)
Completeness spherical (%)	52.4 (9.3)	96.6 (79.5)
Redundancy	13.5 (13.2)	5.5 (5.1)
$CC_{1/2}$	0.99 (0.87)	0.97 (0.40)
**Refinement**		
Resolution (Å)	20.82 – 1.08 (1.12 – 1.08) *	29.14 – 1.69 (1.75 – 1.69)*
No. reflections	25004 (92)	13156 (1094)
$R_{\text{work}}/R_{\text{free}}$	0.180 / 0.209	0.165 / 0.189
No. atoms		
Protein	902	994
Ligand/ion	1	1
Water	147	98
B-factors		
Protein	25.72	24.01
Ligand/ion	32.26	40.42
Water	41.11	40.75
R.m.s. deviations		
Bond lengths (Å)	0.006	0.005
Bond angles (°)	0.72	0.66
Ramachandran		
Favored (%)	98.08	97.22
Allowed (%)	1.92	2.78
Clashscore	1.68	0.57
PDB ID	**9I2I**	**9T8Z**

* Values in parentheses are for the highest-resolution shell.

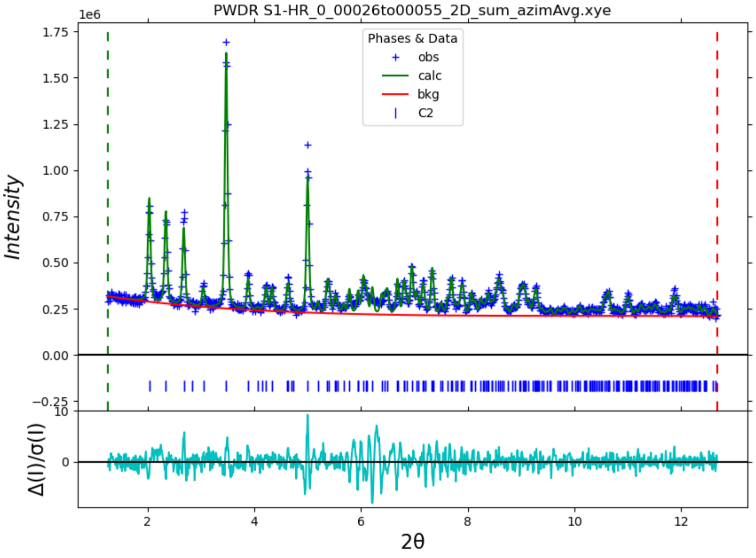

Figure S3: Powder X-ray diffraction of GB1$_{QDD}$ microcrystals. All observable peaks in the microcrystalline diffraction pattern could be indexed with the same unit cell and space group as the single-crystal XRD structure.

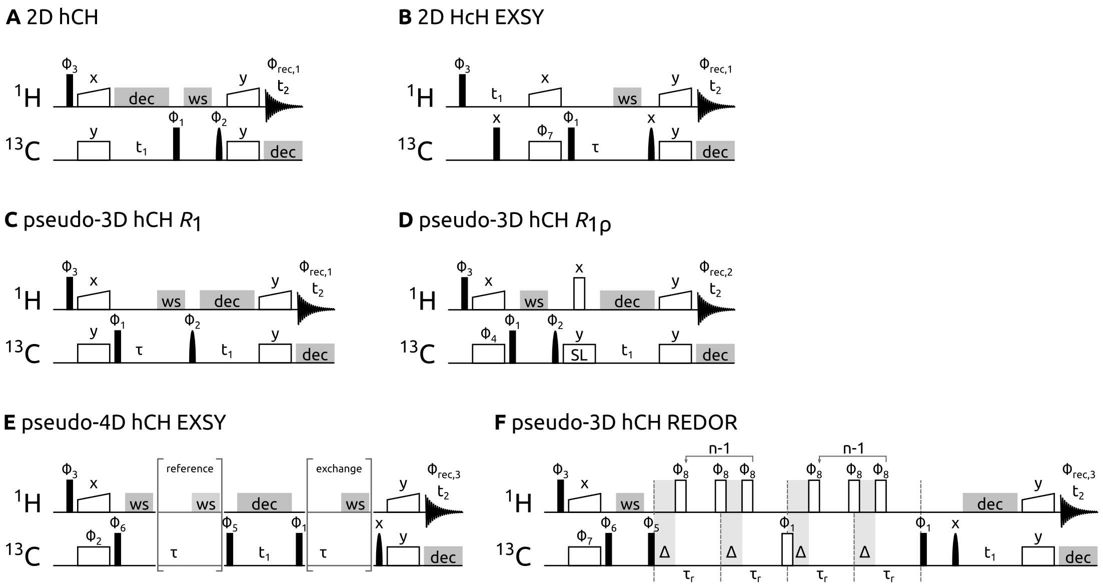

Figure S4: Pulse sequences used in this study for aromatics. Narrow black rectangles denote 90° pulses, open rectangles denote 180° pulses and rounded black symbols denote selective ^{13}C excitation pulses (EBURP2). Grey rectangles indicate decoupling (dec) and water suppression (ws) elements. Wide open symbols denote cross-polarization elements. Indirect and direct acquisition times are indicated with t_1 and t_2 respectively. Phase cycles are: Φ_1 = x,-x; Φ_2 = 2(x),2(-x); Φ_3 = 4(y),4(-y); Φ_4 = 8(y),8(-y); Φ_5 = 8(x),8(-x); Φ_6 = 16(x),16(-x); Φ_7 = 2(y),2(-y); Φ_8 = XY-8; $\Phi_{rec,1}$ = y,-y,-y,y, -y,y,y,-y; $\Phi_{rec,2}$ = y,-y,-y,y, 2(-y,y,y,-y), y,-y,-y,y; $\Phi_{rec,3}$ = y,-y,-y,y, 2(-y,y,y,-y), y,-y,-y,y, -y,y,y,-y, 2(y,-y,-y,y), -y,y,y,-y.

Table S2: Experimental NMR parameters.

Figures	Experiment	Fig. S4	Sample	Probe [mm]	ν_r [kHz]	T_{set} [K]	T_{sample} [K]	t_{exp} [h]	$t_1/t_2/t_{dir}$[a][ms]	w.s.[b][kHz]	dec. $^1H/^{13}C/^{15}N$[b][kHz]	Comment
2A	hNH		(2), crystal	1.9	40	245	305	0.5	32/-/15	16	12.5/-/5	
S5	hCANH		(2), crystal	1.9	40	245	305	9	6/22/15	16	12.5/-/5	NUS: 35 %[d]
S5	hCAcoNH		(2), crystal	1.9	40	245	305	16	6/22/15	16	12.5/-/5	NUS: 60 %[d]
S5	hcaCBcaNH		(2), crystal	1.9	40	245	305	23.5	4.5/22/15	16	12.5/-/5	NUS: 70 %[d]
S5	hcaCBcacoNH		(2), crystal	1.9	40	245	305	47	4.5/22/15	16	12.5/-/5	NUS: 70 %[d]
S5	hCONH		(2), crystal	1.9	40	245	305	12	24/22/15	18	12.5/-/5	NUS: 34 %[d]
S5	hCOcaNH		(2), crystal	1.9	40	245	305	21.5	24/22/15	16	12.5/-/5	NUS: 60 %[d]
2C, S11	hNH R_1		(3), crystal	1.9	39	245	304	60.5	32/-/15	16	100/-/5	
2C, S11	hNH $R_{1\rho}$		(3), crystal	1.9	39	245	304	12.5 - 49	32/-/15	16	100/-/5	ν_{SL} = 15, 25, 27.5, 30, 32.5, 35 kHz
2D	hCH		solution			288	288		28/-/61			
2D	hCH	A	(3), crystal	1.3	39	225	288	0.5	8/-/15	25	12.5/5/-	
2D, S10	hCH	A	(3), IgG:GB1	1.9	30	245		73.5	9/-/15	18.5	12.5/15/-	
S7	HNH RFDR		(3), crystal	1.9	40	250	307	45.5	22/4/15	16	12.5/-/5	
S10	HcH EXSY	B	(3), IgG:GB1	1.9	39	245	304	16.5	4.5/-/30	16	7.5/5/-	
3A, S13B	EXSY	E	(3), crystal	1.9	30	271	304	50	10/-/15	16/18.5	100/5/-	
3A	EXSY	E	(3), crystal	1.9	39	245	304	61.5	10/-/15	16/18.5	100/5/-	
3A, S13A	EXSY	E	(3), crystal	1.3	39	225	288	133	8/-/15	16/18.5	100/5/-	
4, S14	hCH REDOR	F	(3), crystal	1.3	39	256	304	138	8/-/15	16	12.5/5/-	Δ = 3 µs
4, S15	hCH REDOR	F	(3), IgG:GB1	1.9	39	245	304	92.5	4/-/30	16	7.5/5/-	Δ = 2.5 µs
4, S17, S19	hCH $R_{1\rho}$	D	(3), crystal	1.9	39	245	304	13.5 - 26.5	10/-/15	16	100/5/-	ν_{SL} = 15, 25, 27.5, 30, 32.5, 35 kHz
4, S16, S19	hCH R_1	C	(3), crystal	1.3	39	225	288	48.5	8/-/15	16	100/5/-	
4, S16, S19	hCH $R_{1\rho}$	D	(3), crystal	1.3	39	225	288	21.5 - 26.5	8/-/15	16	100/5/-	ν_{SL} = 15, 25, 27.5, 30, 32.5, 35 kHz
4, S18, S19	hCH R_1	C	(3), IgG:GB1	1.9	39	245	304	50.5	30/-/-	16	12.5/5/-	
4, S18, S19	hCH $R_{1\rho}$	D	(3), IgG:GB1	1.9	39	245	304	15.5	30/-/-	16	12.5/5/-	ν_{SL} = 15, 25, 27.5, 30, 32.5, 35 kHz

[a] Acquisition time in indirect (t_1/t_2) and direct (t_{dir}) dimension. [b] Water suppression achieved with a composite pulse decoupling scheme based on a modified MISSISSIPPI sequence (*5*). The second value for longitudinal exchange experiments corresponds to the ws block during the mixing time. [c] Composite pulse decoupling during acquisition with swfTPPM (*6*) for ^1H and WALTZ-16 (*7*) for ^{13}C and ^{15}N.
[d] Amount of non-uniform sampling.

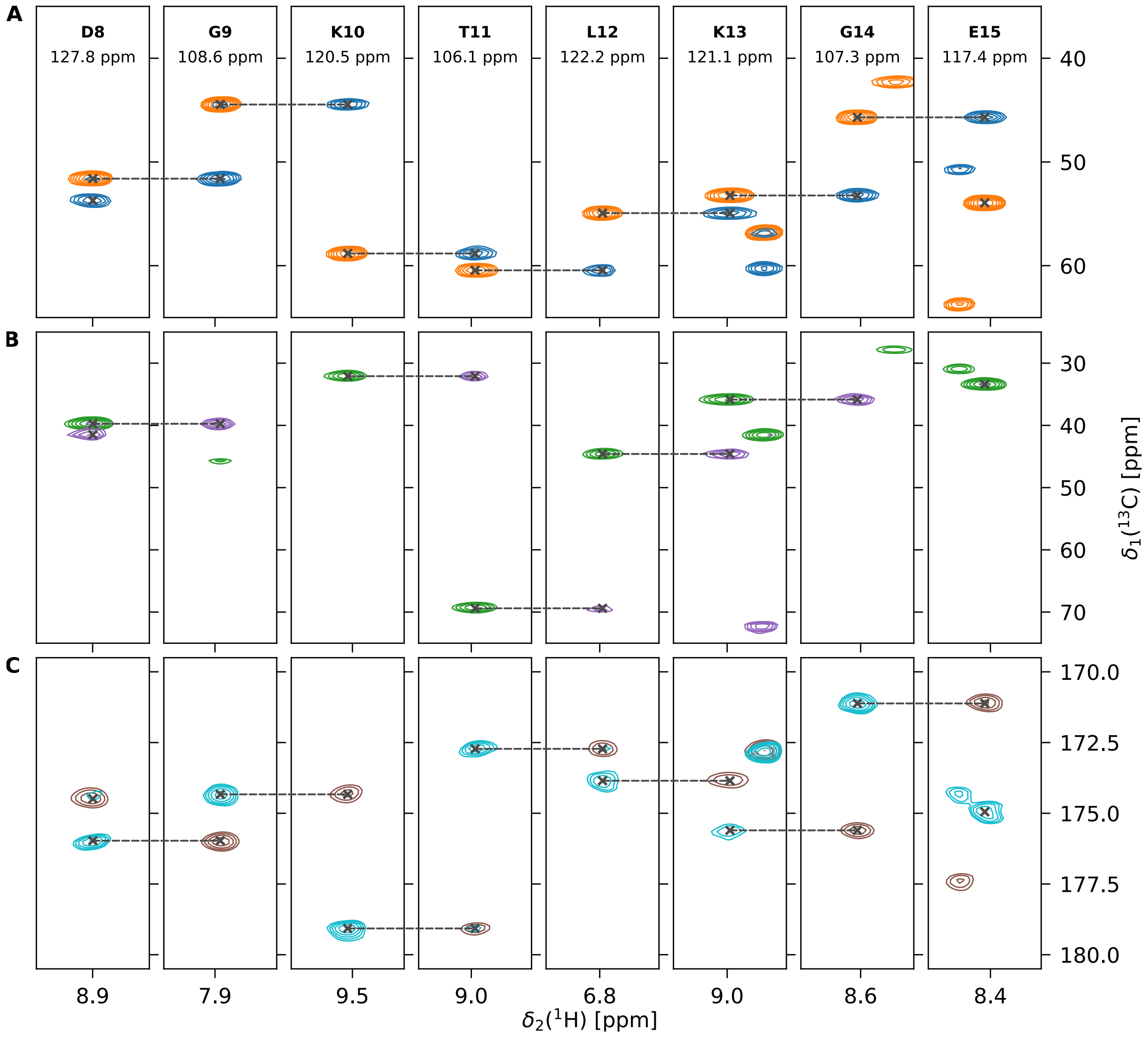

Figure S5: Representative sections of spectra for backbone assignment for residues D8 to E15. Each column shows the plane at the assigned ^{15}N frequency of one residue as indicated at the top. **A** hCANH (orange) and hCAcoNH (blue). **B** hcaCBcaNH (green) and hcaCBcacoNH (purple). **C** hCOcaNH (cyan) and hCONH (brown).

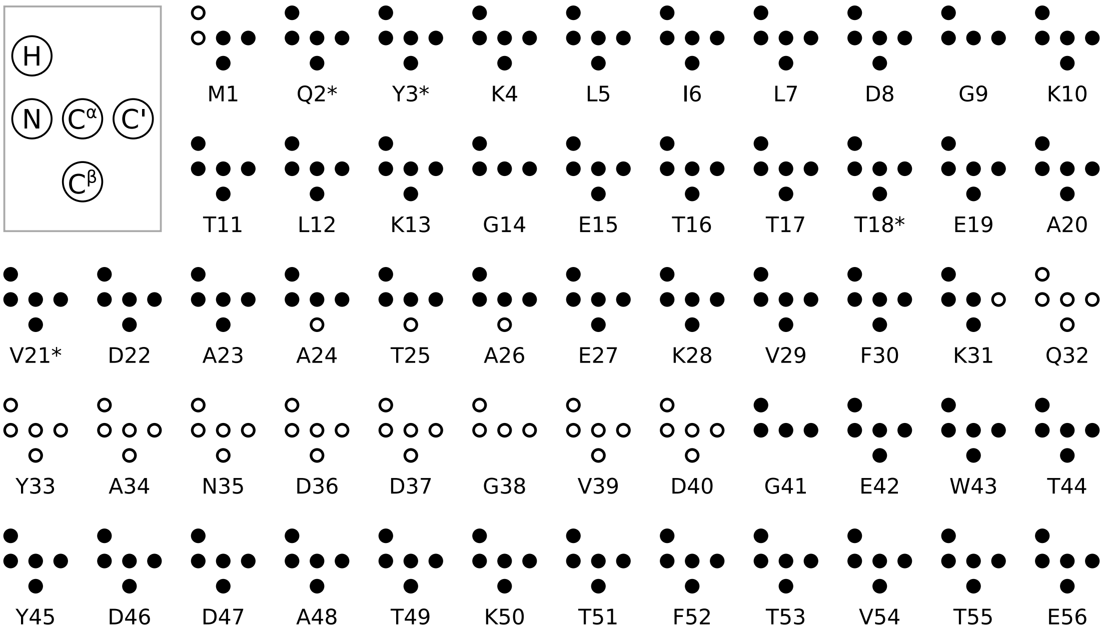

Figure S6: Completeness of the backbone assignment for each residue. Empty and filled circles indicate unassigned and assigned atoms, respectively. Residues that have two sets of resonances are marked with an asterisk.

Table S3: Chemical shift assignments in [ppm]. Residues with two rows have two sets of resonances.

Residue	H^N	N^H	C'	C^α	C^β	Residue	H^N	N^H	C'	C^α	C^β
M1			170.70	54.19	31.51	E27	8.49	116.23	177.28	59.21	28.03
Q2	8.10	123.38	174.22	55.12	29.23	K28	6.88	116.05	179.33	59.08	31.18
	8.31	125.58	174.42	55.69	29.53	V29	7.50	119.54	178.67	65.79	30.79
Y3	8.65	119.83	174.82	56.81	43.31	F30	8.64	118.38	178.55	57.17	37.26
	8.87	123.60	174.94	56.81	42.58	K31	8.85	120.23		59.48	31.18
K4	9.20	122.33	172.85	54.56	34.75	Q32					
L5	8.73	126.84	174.50	52.66	42.11	Y33					
I6	9.28	125.89	174.69	59.49	37.18	A34					
L7	8.95	126.40	174.49	53.72	41.51	N35					
D8	8.90	127.76	175.97	51.61	39.74	D36					
G9	7.86	108.58	174.33	44.46		D37					
K10	9.53	120.55	179.07	58.82	32.09	G38					
T11	8.97	106.12	172.72	60.45	69.39	V39					
L12	6.78	122.23	173.85	54.93	44.59	D40					
K13	8.98	121.10	175.60	53.23	35.85	G41	7.60	104.34	171.93	44.30	
G14	8.62	107.31	171.12	45.70		E42	8.39	119.17	176.26	54.95	30.56
E15	8.44	117.38	174.95	53.94	33.41	W43	8.85	131.80	176.29	57.62	28.72
T16	8.89	115.48	171.86	60.29	69.22	T44	9.56	114.84	172.80	60.27	72.15
T17	8.20	111.82	174.15	60.00	72.80	Y45	8.73	121.29	172.85	56.85	41.53
T18	8.99	111.77	171.50	62.15	69.40	D46	7.78	127.60	174.88	51.51	43.08
	9.09	113.33	171.22	61.89	69.43	D47	8.89	125.22	178.39	56.56	42.59
E19	7.78	124.68	175.41	54.13	31.06	A48	8.20	118.17	179.10	54.48	17.64
A20	9.51	126.15	177.55	50.64	22.50	T49	6.90	101.86	174.90	59.88	69.91
V21	8.61	117.14	174.28	63.43	30.97	K50	7.83	123.25	174.95	55.58	28.68
	9.17	114.73	175.09	62.98	31.09	T51	7.57	110.97	174.87	61.92	71.87
D22	7.24	114.27	175.67	52.53	41.51	F52	10.38	130.16	174.59	56.89	41.65
A23	9.54	122.14	179.17	54.06	16.73	T53	9.08	117.94	172.65	61.50	70.39
A24	7.89	120.30	181.06	54.21		V54	8.33	124.27	173.49	58.45	31.36
T25	8.29	116.73	175.57	67.08		T55	8.11	122.94	174.14	61.18	70.25
A26	7.46	123.36	177.22	54.60		E56	7.97	133.24	180.11	57.15	32.28

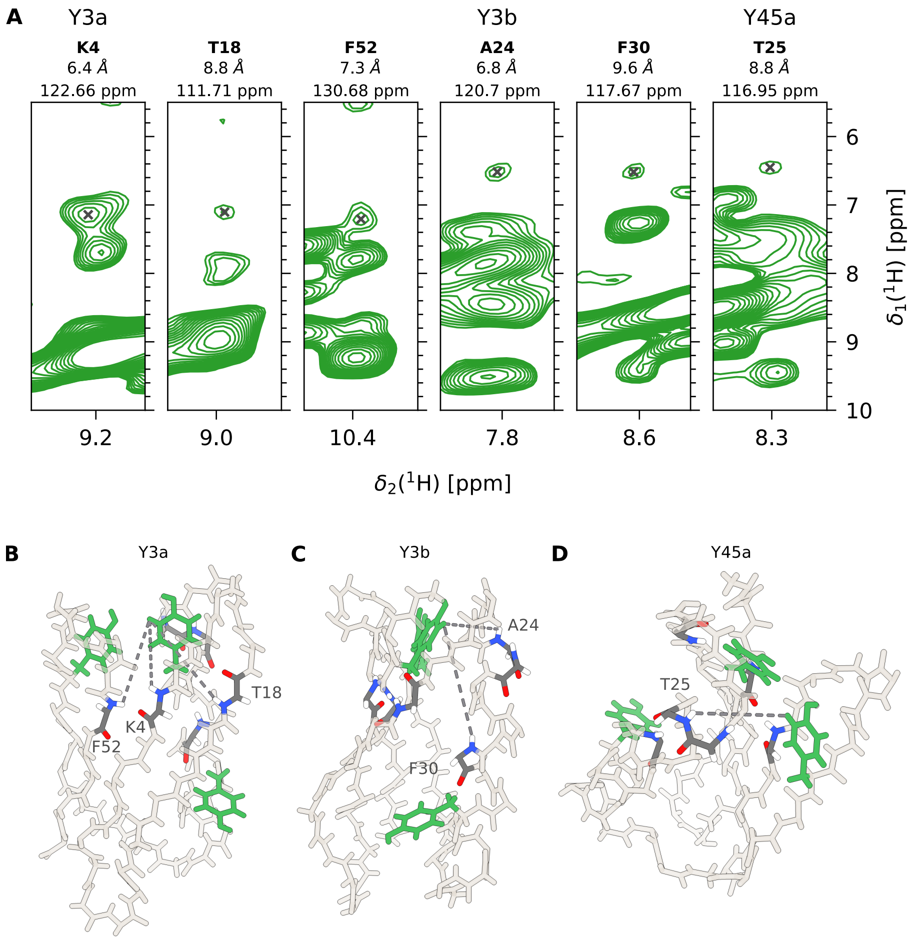

Figure S7: Assignment of $(CH)^{\epsilon}$-labeled tyrosines in $GB1_{QDD}$ crystals by a 3D HNH RFDR spectrum. (A) Sections of a 3D HNH RFDR spectrum showing cross peaks between the ^{1}H frequency of $(CH)^{\epsilon}$-labeled tyrosines and the ^{1}H frequency of backbone amide proton signals. The header of each strip indicates the assigned tyrosine signal, the residue of the backbone signal, the distance between the two atoms, and the ^{15}N frequency of the shown plane. (B-D) 3D representation of $GB1_{QDD}$ indicating the observed cross peaks from (A) as connections between the atoms. The backbone is represented by beige, and the three tyrosines by green sticks. Residues observed in cross peaks are highlighted in dark gray. Each panel shows the connections of one tyrosine as indicated on top.

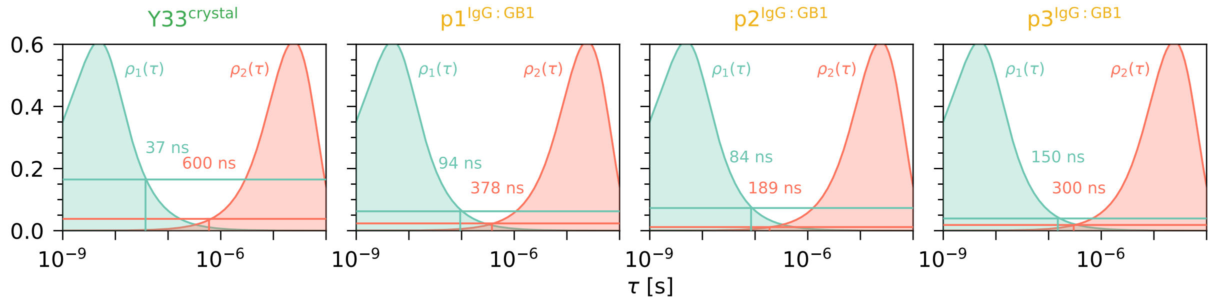

Figure S8: Estimation of timescales for tyrosines Y33^{crystal}, and p1, p2, and p3 of the complex. If a dynamic process has a correlation time τ equal to the maximum of the detector sensitivity, the response to this sensitivity equals $(1 - S^2)$, which is 0.609 for a ring flip. All responses we obtained are much lower than this value, indicating that none of the residues flip at a timescale corresponding to the maximum of one of the sensitivities. We know from the dipolar order parameters that Y33^{crystal}, p1, p2, and p3 flip faster than hundreds of microseconds. As ring flips faster than tens of nanoseconds are unlikely, we assume that these residues flip on a timescale that lies in the 'blind spot' between ρ_1 and ρ_2. This blind spot is due to a lack of experiments that can accurately probe dynamics on this timescale. Here, we show a close-up of ρ_1 (blue) and ρ_2 (red), which are scaled by $(1 - S^2) = 0.609$. The horizontal lines are the detector responses for the two sensitivities, respectively. The intercepts of the response and the sensitivity are the minimum and maximum timescales that define the range in which the ring should flip. Figure 4D of the main text shows this in a compacted way.

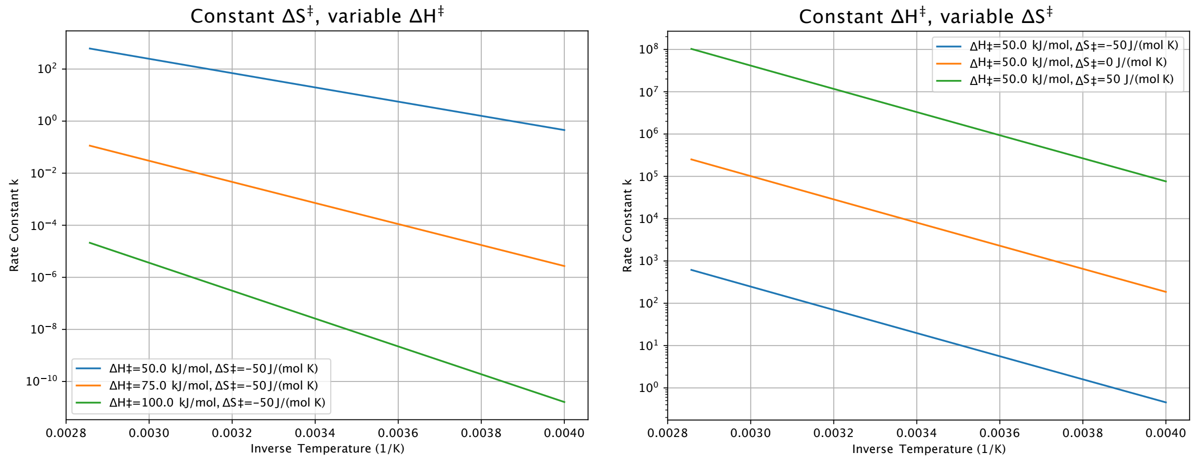

Figure S9: Model calculations of exchange rate constants with the Eyring equation, assuming different values of $\Delta H^{\ddagger}$ and $\Delta S^{\ddagger}$. This data illustrates that a flatter dependency of log k vs $1/T$ is due to smaller $\Delta H^{\ddagger}$. Moreover, these calculations support that the reduced rate constants in crystals is due to reduced $\Delta S^{\ddagger}$. The temperature range shown here is 250 to 350 K.

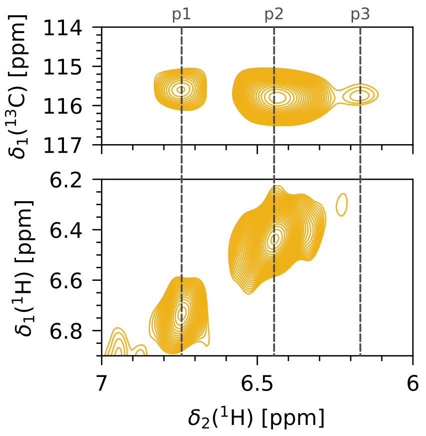

Figure S10: Dipolar-based $^{1}H-^{13}C$ 2D MAS NMR spectrum (upper panel) and $^{13}C-^{13}C$ EXSY spectrum with 256 ms exchange time and 2D $^{1}H-^{1}H$ readout (lower panel) of IgG:GB1$_{QDD}$. The $^{1}H-^{13}C$ spectrum is the same as the yellow spectrum in Figure 2D of the main text. The ^{1}H frequencies of p1, p2, and p3 are marked for better comparison to the EXSY spectrum. No cross-peaks can be observed in the EXSY spectrum, confirming that the three peaks belong to the three different tyrosines. If one of the tyrosines was in slow exchange and would therefore result in two signals, these two signals should show exchange cross-peaks in the EXSY experiment. For experimental details, see Table S2.

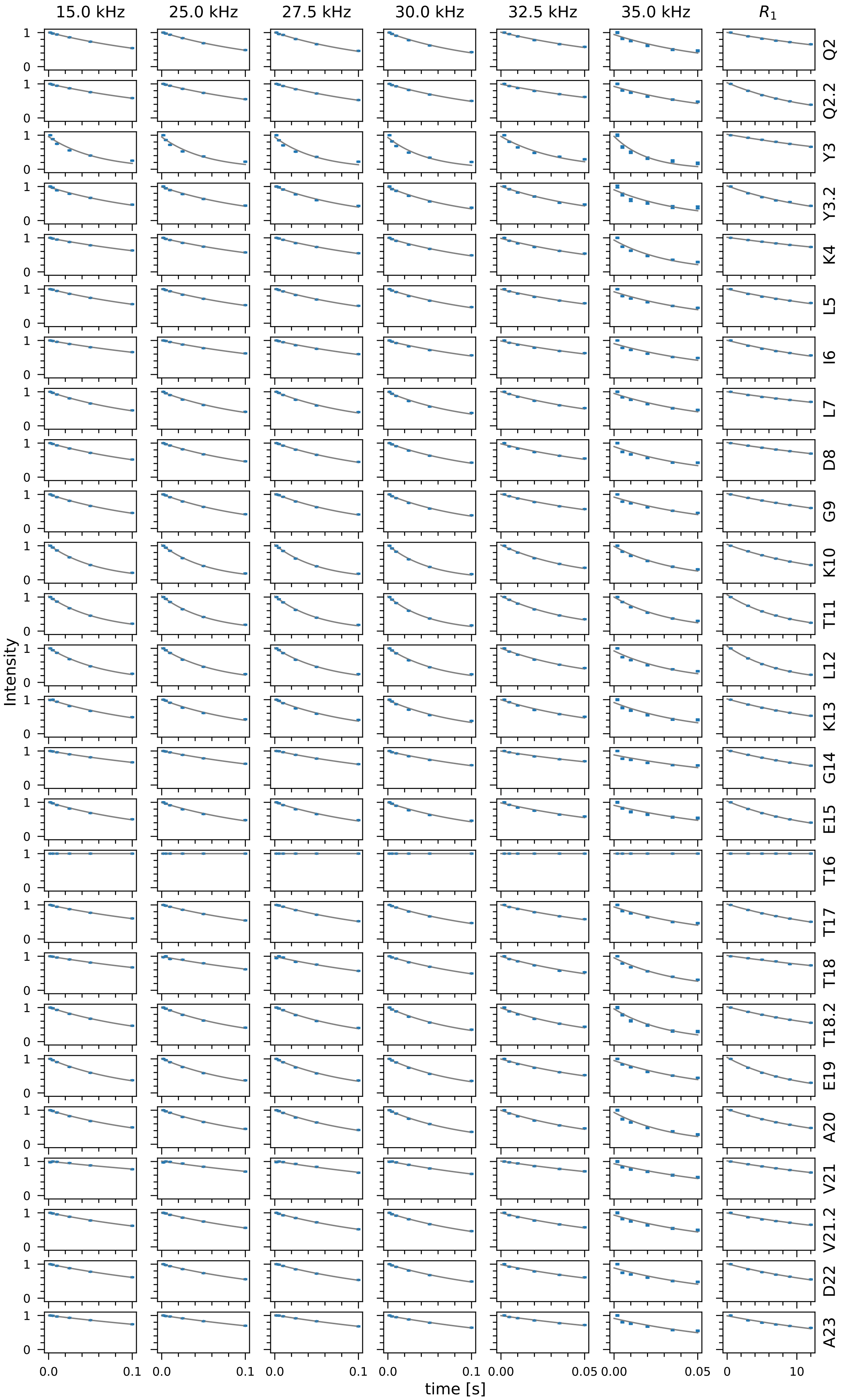

Figure S11: Continued on next page.

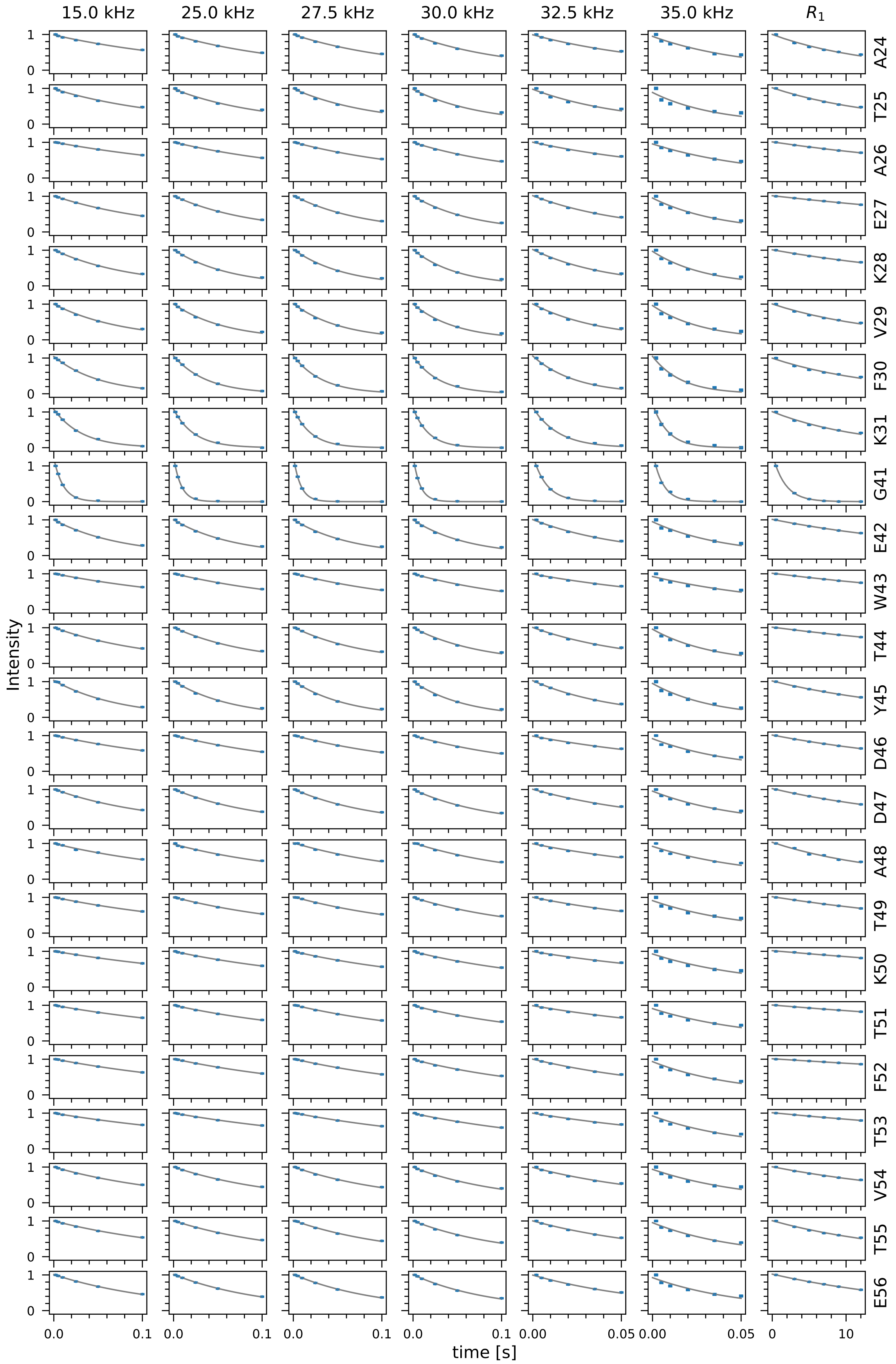

Figure S11: Continued. Exponential fits (gray) of experimental decay curves (blue) for ^{15}N R_1 and $R_{1\rho}$ relaxation rate measurements of backbone amides in GB1$_{\mathrm{QDD}}$ crystals at a MAS frequency of 39 kHz and a temperature of 304 K. $R_{1\rho}$ was measured at ν_{SL} = 15, 25, 27.5, 30, 32.5 and 35 kHz.

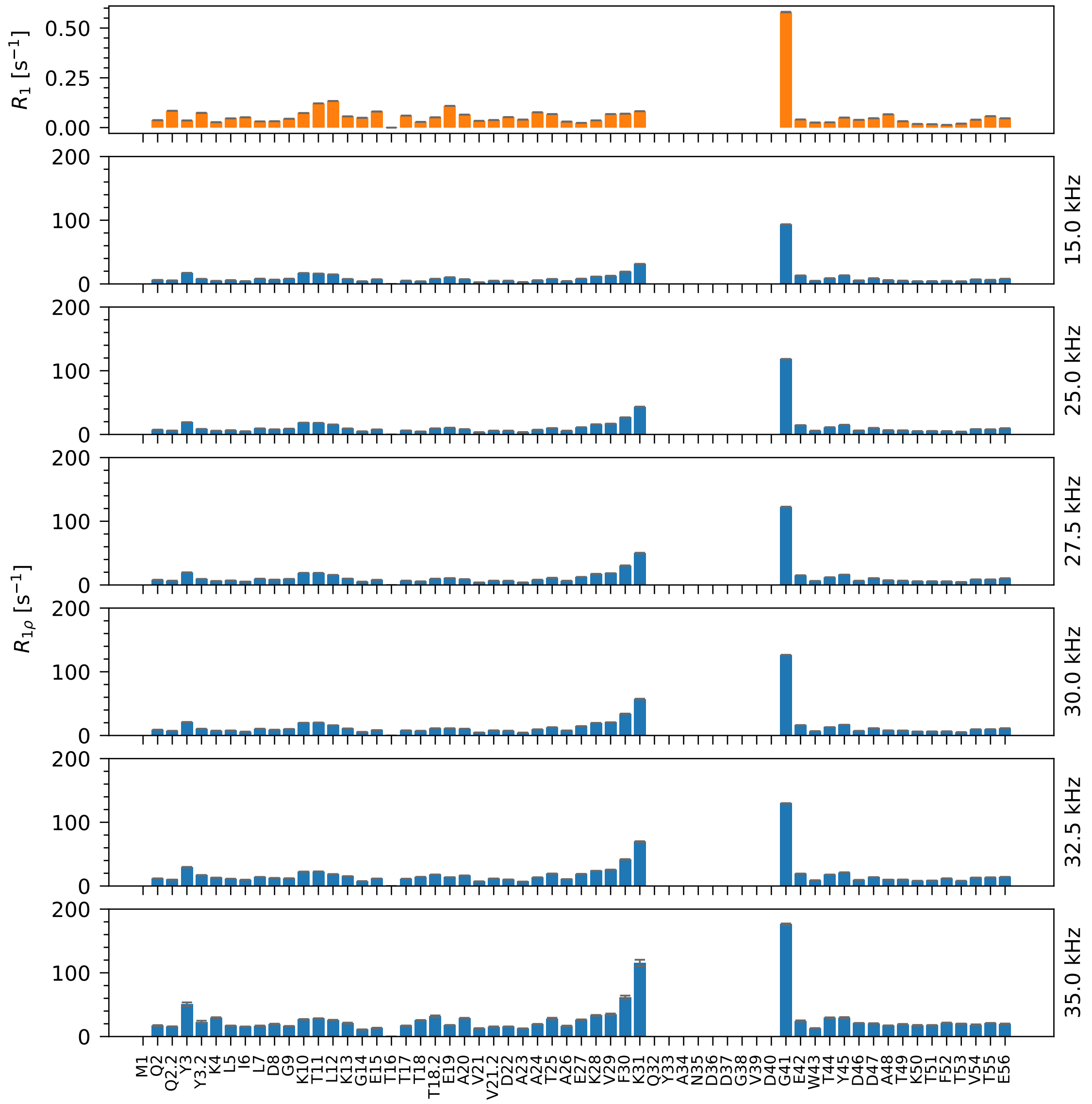

Figure S12: Residue-wise ^{15}N R_1 (top panel) and $R_{1\rho}$ relaxation rate constants at ν_{SL} = 15, 25, 27.5, 30, 32.5 and 35 kHz as specified on the right side for crystalline GB1$_{QDD}$. Two values are reported for the residues that show two signals (Q2, Y3, T18, and V21).

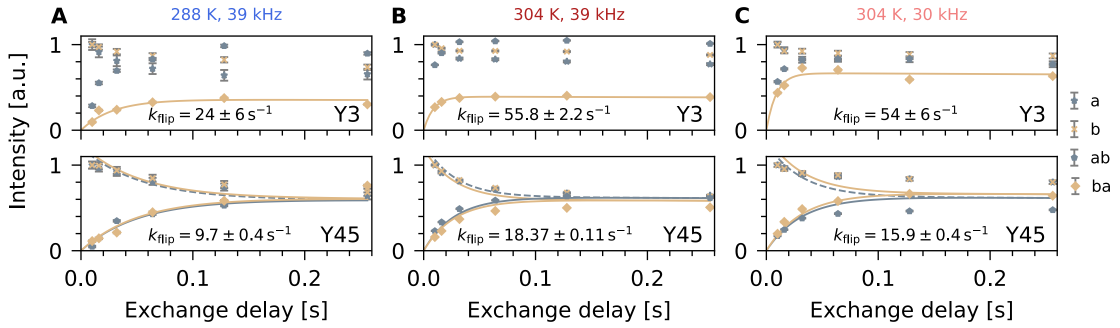

Figure S13: EXSY build-up and decay curves for Y3 and Y45. For Y3, only the build-up of 'ba' was fitted as the intensities of the other signals were strongly impacted by peak overlap with Y33. For Y45, we performed a combined analysis of all four peaks with the populations fixed at 50 % (*8*). (A) Measured at a MAS frequency of 39 kHz and a temperature of 288 K. (B) Measured at a MAS frequency of 39 kHz and a temperature of 304 K. (C) Measured at a MAS frequency of 30 kHz and a temperature of 304 K.

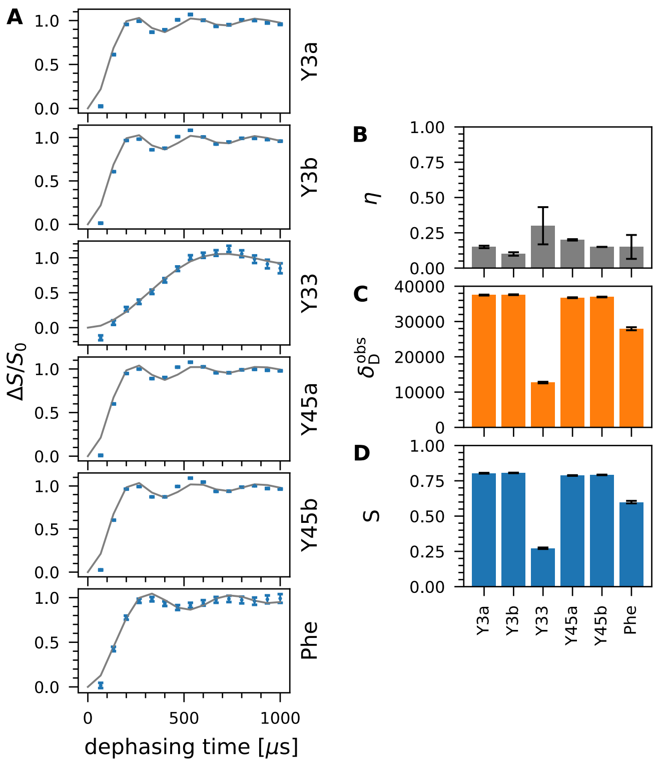

Figure S14: Determination of dipolar order parameters of $(CH)^{\epsilon}$-labeled tyrosines and phenylalanines in $GB1_{QDD}$ crystals. (A) Experimental (blue) and best-fit simulated (gray) REDOR curves. (B) Best-fit η. (C) Best-fit δ_D. (D) Dipolar order parameter.

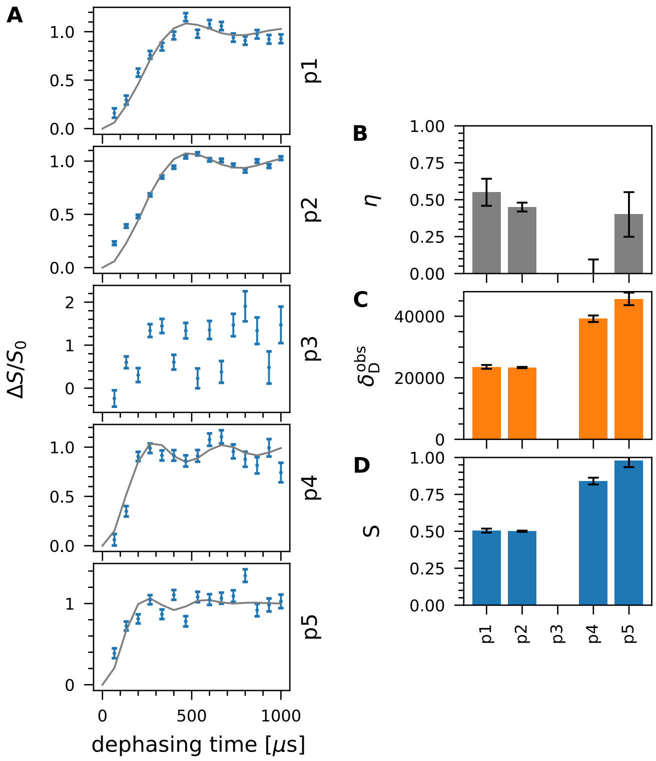

Figure S15: Determination of dipolar order parameters of $(CH)^{\epsilon}$-labeled tyrosines and phenylalanines in the $IgG{:}GB1_{QDD}$ complex. (A) Experimental (blue) and best-fit simulated (gray) REDOR curves. p3 has very low sensitivity which is why we did not fit the data. (B) Best-fit η. (C) Best-fit δ_D. (D) Dipolar order parameter.

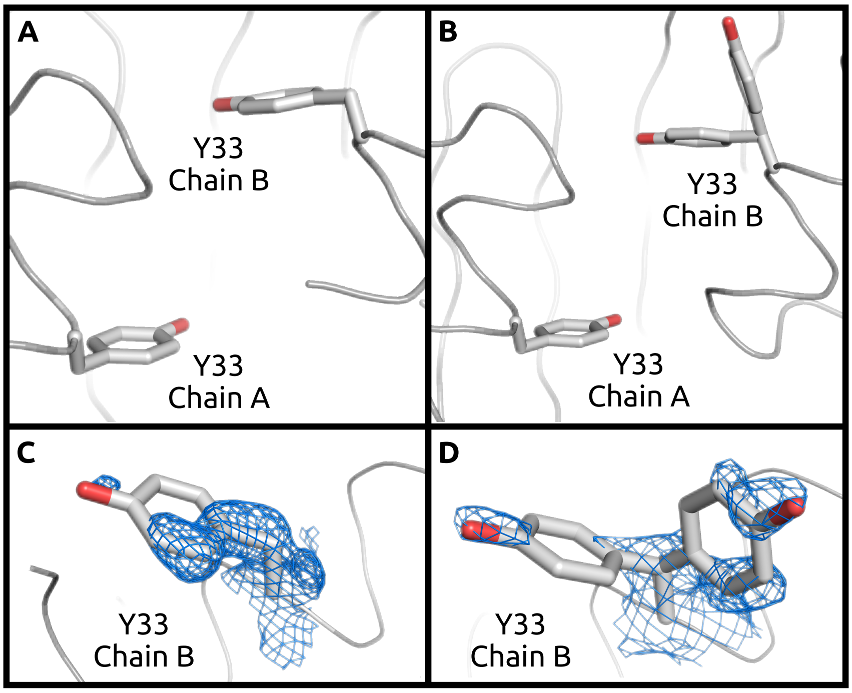

Figure S16: Alternate conformations of Y33 in GB1 at room temperature. (A, B) Same view of the Y33 residue in chains A and B of the GB1 structure determined at 100 K (A) and 295 K (B). (C, D) Close-up of the Y33 side chain from chain B, showing a single conformation in the 100 K structure (C) and an alternate side-chain conformation in the room-temperature structure (D). The 2Fo–Fc electron-density maps are shown as blue mesh contoured at 0.8σ.

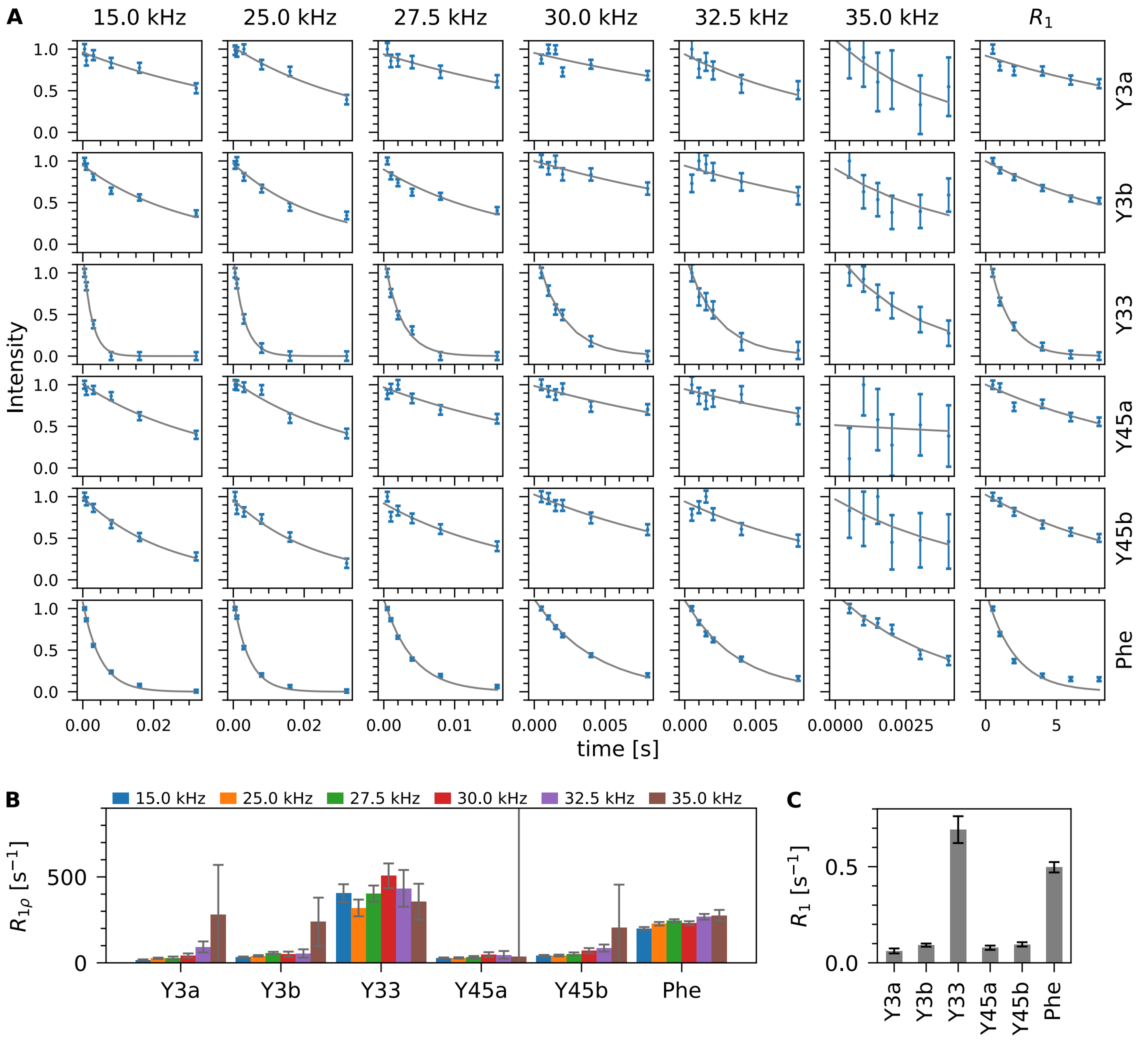

Figure S17: ^{13}C R_1 and $R_{1\rho}$ relaxation rate constants of $(CH)^{\epsilon}$-labeled tyrosines and phenylalanines in GB1$_{QDD}$ crystals at a MAS frequency of 39 kHz and a temperature of 288 K. $R_{1\rho}$ was measured at ν_{SL} = 15, 25, 27.5, 30, 32.5 and 35 kHz. (A) Exponential fits (gray) of experimental decay curves (blue). (B) Fitted $R_{1\rho}$ relaxation rate constants at different spin-lock fields. (C) Fitted R_1 relaxation rate constants.

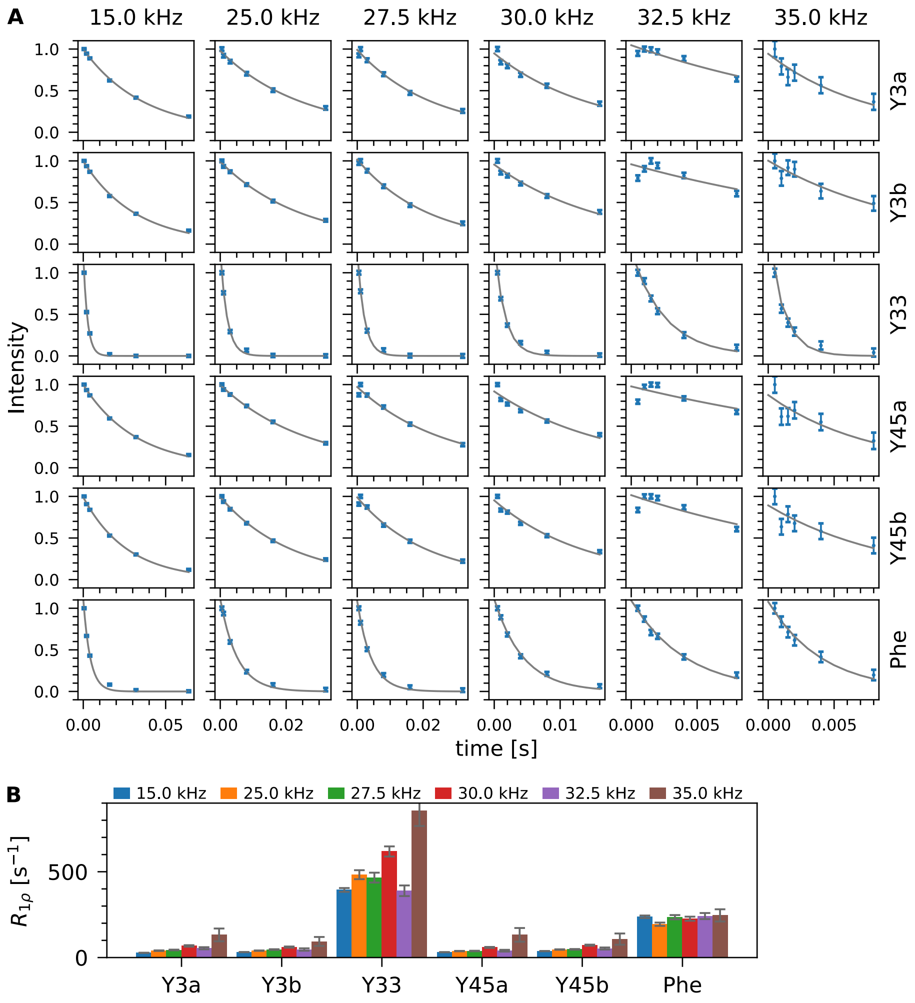

Figure S18: ^{13}C $R_{1\rho}$ relaxation rate constants of (CH)$^{\epsilon}$-labeled tyrosines and phenylalanines in GB1$_{QDD}$ crystals at a MAS frequency of 39 kHz and a temperature of 304 K. $R_{1\rho}$ was measured at ν_{SL} = 15, 25, 27.5, 30, 32.5 and 35 kHz. (A) Exponential fits (gray) of experimental decay curves (blue). (B) Fitted $R_{1\rho}$ relaxation rate constants at different spin-lock fields.

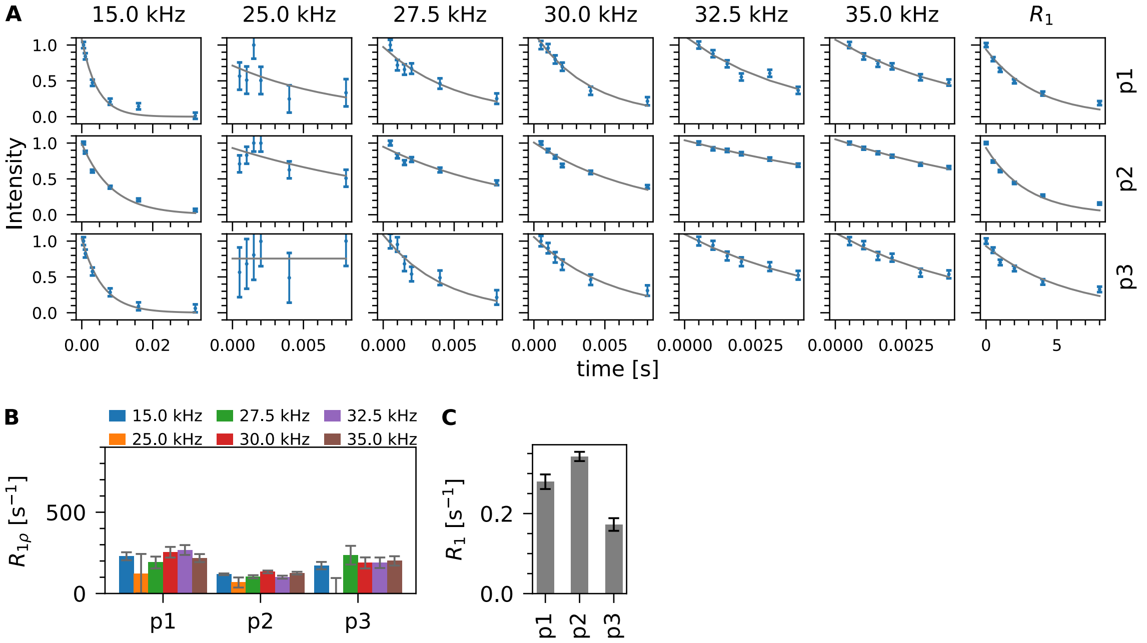

Figure S19: ^{13}C R_1 and $R_{1\rho}$ relaxation rate constants of (CH)$^\epsilon$-labeled tyrosines and phenylalanines in the IgG:GB1$_{QDD}$ complex at a MAS frequency of 39 kHz and a temperature of 304 K. $R_{1\rho}$ was measured at ν_{SL} = 15, 25, 27.5, 30, 32.5 and 35 kHz. (A) Exponential fits (gray) of experimental decay curves (blue). (B) Fitted $R_{1\rho}$ relaxation rate constants at different spin-lock fields. (C) Fitted R_1 relaxation rate constants.

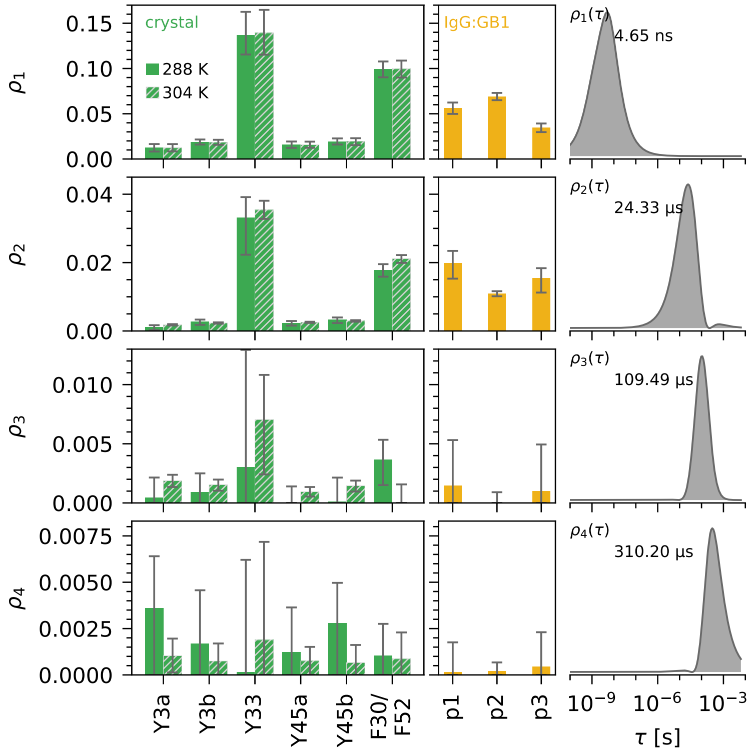

Figure S20: Detectors analysis for $(CH)^{\epsilon}$-labeled tyrosines and phenylalanines in GB1$_{QDD}$ crystals (green) and in the IgG:GB1$_{QDD}$ complex (yellow). The detector sensitivities $\rho_i(\tau)$ are shown on the right. The correlation time corresponding to the maximum of $\rho_i(\tau)$ is specified. The bar plots show the corresponding responses ρ_i for each residue for the two samples. The crystal was measured and analyzed at two temperatures. R_1 was only measured at the lower temperature but included in both analyses as it is not expected to be very temperature-dependent in our available temperature range.

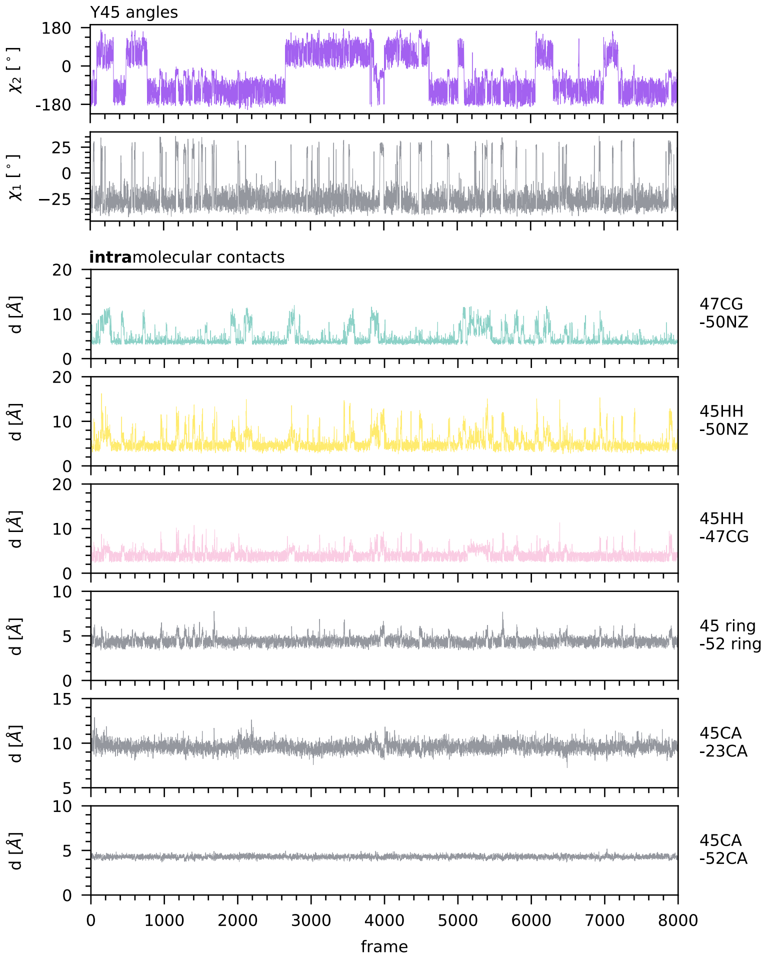

Figure S21: Traces of selected angles and distances from solution MD simulations of GB1 in solution. The parameters represent the interaction network of residue Y45 in solution. The first five parameters are shown in parts for the simulation of the crystal in the main text (Fig. 6). The last three distances were selected as indicators for breathing motions and are visualized in Figure S23.

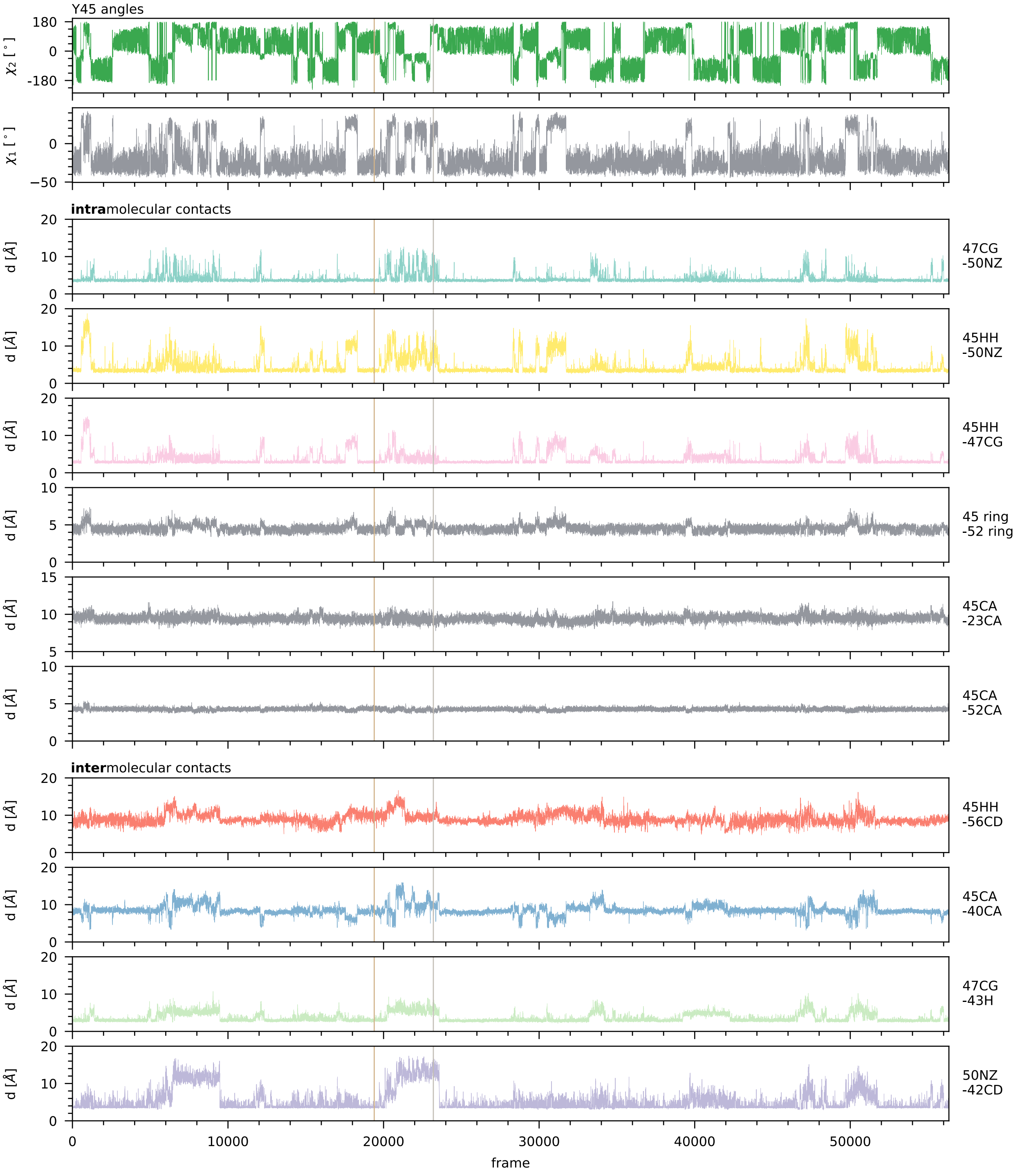

Figure S22: Traces of selected angles and distances from crystal MD simulations of crystalline GB1. The parameters represent the interaction network of residue Y45 in the crystal. Sections of these traces are shown in Figure 6G of the main text. The last three intramolecular distances were selected as indicators for breathing motions and are visualized in Figure S23. The horizontal lines indicate the frames that are shown in the structure visualization in Figures 6A-F and S23.

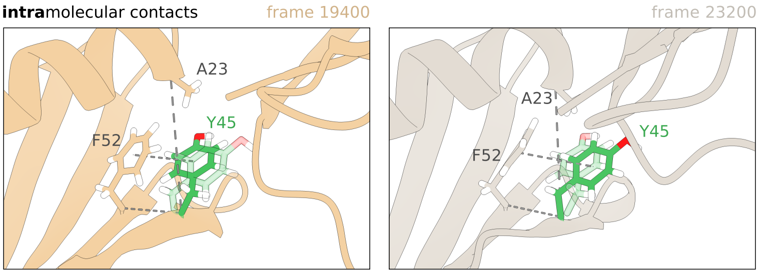

Figure S23: Visualization of distances that were selected as indicators for breathing motions. Their traces are shown in Figures S21 and S22 but not in Figure 6 of the main text. The distances are shown for two different frames of the crystal simulations, representing a state in which Y45 is oriented towards the core of GB1 (frame 19400; brown) or outward rotated (frame 23200; gray). We initially hypothesized that flips of Y45 might be coupled to global "breathing" motions. While this term lacks a precise definition, we analyzed fluctuations in the compactness of the hydrophobic core by monitoring several distances across the core of the protein such as the distance between the C^{α} atoms of residues 45 (located in β-strand 3) and 23 (located in the α-helix). No detectable correlation was observed between fluctuations in the Y45 ring-axis orientation (χ_1) or ring-flip state (χ_2) and fluctuations of these long-range distances. This finding suggests that ring flips are not directly associated with a simple expansion or contraction of the hydrophobic core.

References

(*1*) Aebischer, K. et al. (2024). Evaluating the motional timescales contributing to averaged anisotropic interactions in MAS solid-state NMR. *Magn. Reson. 5*, 69–86.

(*2*) Gauto, D. F. et al. (2019). Aromatic ring dynamics, thermal activation, and transient conformations of a 468 kDa enzyme by specific ^{1}H–^{13}C labeling and fast magic-angle spinning NMR. *Journal of the American Chemical Society 141*, 11183–11195.

(*3*) Smith, A. A., Ernst, M., and Meier, B. H. (2018). Optimized "detectors" for dynamics analysis in solid-state NMR. *J. Chem. Phys. 148*, DOI: 10.1063/1.5013316.

(*4*) Tatman, B. P. The development of methods to study structure and dynamics in biological systems using solid-state NMR, Available at https://wrap.warwick.ac.uk/id/eprint/185489/, PhD thesis, University of Warwick, 2023.

(*5*) Zhou, D. H., and Rienstra, C. M. (2008). High-performance solvent suppression for proton detected solid-state NMR. *Journal of Magnetic Resonance 192*, 167–172.

(*6*) Thakur, R. S., Kurur, N. D., and Madhu, P. (2006). Swept-frequency two-pulse phase modulation for heteronuclear dipolar decoupling in solid-state NMR. *Chemical Physics Letters 426*, 459–463.

(*7*) Shaka, A. et al. (1983). An improved sequence for broadband decoupling: WALTZ-16. *Journal of Magnetic Resonance (1969) 52*, 335–338.

(*8*) Farrow, N. A. et al. (1994). A heteronuclear correlation experiment for simultaneous determination of 15N longitudinal decay and chemical exchange rates of systems in slow equilibrium. *Journal of Biomolecular NMR 4*, 727–734.
