# Supplementary material for: Aromatic ring flips reveal reshaping of protein dynamics in crystals and complexes
Source: Nat Chem. 2026 Jun 10;18(7):1221–30. doi: 10.1038/s41557-026-02155-0 (PMC13323056; doi:10.1038/s41557-026-02155-0)
Supplement: Supplementary file 2 — Peer Review File [file 41557_2026_2155_MOESM2_ESM.pdf]

nature portfolio

Peer Review File

Aromatic Ring Flips Reveal Reshaping of Protein Dynamics in Crystals and Complexes

Corresponding Author: Professor Paul Schanda

Version 0:

Author Rebuttal letter:

Reviewer #1 (Comments for the Author):

1. The GB1 variant is quite interesting. Are there any data on any difference in stability (e.g., melting temperature) compared to T2Q and WT GB1? This could be useful to discuss, especially in context of the proposed ‘distortion’ of the GB1 fold.
The melting temperatures Tm of GB1 T2Q and QDD at pH 5.4 (maximum stability) are 87.5 °C and 74 °C, respectively (Lindman et al., 2006 https://doi.org/10.1529/biophysj.105.071050). We have measured melting curves for these two variants at the conditions used in the manuscript (50 mM NaP, pH 6.5). We find an overall similar conclusion, namely that the T2Q variant is slightly more stable than QDD. At the conditions of this study, QDD is fully folded and stable.

Unfolding (left) and refolding (right) curves for GB1 T2Q (blue) and QDD (orange) measured and analyzed with the nanotemper Prometheus Panta. Curves were measured between 25 and 95 °C in steps of 1 °C/min with protein concentrations of 1.3 and 1.6 mg/mL for T2Q and QDD, respectively.
The first and the second rows show the intensity ratios between 350 nm and 330 nm, and their derivatives, respectively. Vertical lines indicate the inflection points (IP).
The third row shows the individual intensity curves measured at 330 nm (dotted) and 350 nm (solid line). Vertical lines indicate the inflection points.
The last row shows the cumulant radius.
method IP T2Q [°C] IP QDD [°C]

(I350nm/I330nm) 67.70 ± 0.42 64.05 ± 0.13

I330nm 65.82 ± 0.04 54.96 ± 0.08

I350nm 65.76 ± 0.04 54.97 ± 0.09

In our manuscript, we discuss the differences between different forms (solution, crystal, complex) of

GB1 QDD, and not between different variants of GB1. We therefore don't find this data relevant in the context of our manuscript and prefer not to discuss it.
2. Moreover, the manuscript describes the modeling of the GB1-IgG interaction based on the WT mode of binding. It would be useful to discuss briefly the positioning of the introduced D amino acids relative to the binding interface. Is there a chance that it may be affected?

We thank the reviewer for this suggestion. Given that there are no crystal structures of full-length IgG with any GB1 variants, the model we propose is indeed somewhat speculative: it is based on the crystal structures of GC2 or GB3 with IgG fragments. There are no crystal structures (even of IgG fragments) with the T2Q or QDD GB1 variants. We cannot assess whether the introduced mutations change the binding mode.
With these limitations in mind, we propose that the three mutations of the QDD variant have only limited (if any) impact on the binding: two of the three mutation sites are quite distant from the binding interface. The mutation that is closest to the interface (N37D) introduces a rather small change (Asn to Asp); its closest neighbor on the IgG side is a Ser, such that it is unlikely that the interaction gets significantly affected by the N37D mutation.
To make it easier for the reader to locate the mutation sites, we have indicated them on the structure in Figure 1F and made a note in the figure caption.
Note that the message of the paper does not really depend on the structural details of the IgG:GB1 complex, as we use this complex only to highlight the interesting finding that ring flips are accelerated, without performing an actual structural analysis of the origins.

3. A very useful feature of the paper is the attempt to verify the new (single crystal) X-ray structure with the microcrystals used for the NMR analysis. In that context the manuscript notes that "we performed powder XRD-like measurements on wet microcrystalline samples closely resembling those used for NMR,". What does this mean exactly? How was it established that they 'closely resemble"? (if not by X-ray)

The samples for NMR and powder X-ray diffraction were obtained not only in similar but identical conditions. Our original formulation was misleading and we have now corrected these sentences (page 4, right column, top third) to clarify this.

4. On page 7, the manuscript mentions the order parameter expected for a ring flipping scenario. But it does not explain how this parameter is determined and what the expected value is. Please add this information and quantitatively compare all of the measured S values to this expected number for ring flip.

The values of the order parameter and asymmetry were originally obtained by a numerical procedure, i.e. by explicit simulations of REDOR recoupling curves in the presence of explicit 120° jumps, in the publication by Gauto et al. (reference 72). In brief, the REDOR curve was numerically calculated with the jump model, and then it was fitted with REDOR simulations in which the order parameter and asymmetry are fit parameters. The values of S=0.625 and eta=0.6 were obtained with a chi-square fitting procedure there.
We have added a more explicit statement now as follows (page 7):
"Gauto et al. modeled the motional averaging due to a ring flip with simulations, and determined results in a theoretical value of S = 0.625;71 reductions below this value point to additional motions, such as the libration of the ring within each rotamer state, motion of the backbone, or the ring axis (i.e., the χ1 angle).
[...]
In contrast, the order parameters of Y33 and the phenylalanines are lower than the expected value for a ring flip (approximately 0.3 and 0.5, respectively), indicating that these rings flip faster than roughly one hundred μs."

5. At the end of page 7 (and top of next page), the manuscript talks about upper and lower bounds of the rate constants (with reference to Figure 4 and the SI). To me, this explanation was too brief and not entirely clear.

We added more explanation in the main text, as follows:
"The expected response of a dynamic process that occurs on a timescale corresponding to the maximum of a detector sensitivity is (1 − S2). The responses that we obtain are clearly lower than the expected value for a ring flip, which is (1 − S2) = 0.609 (Figure 4B, lower left panel). With the assumption that rings do not flip faster than hundreds of microseconds and the information from the order parameters, we conclude that Y33crystal flips on a timescale corresponding to the blind spot between ρ1(τ) and ρ2(τ) (Figure 4B, blue rectangle). By scaling ρ1(τ) and ρ2(τ) with (1 − S2) = 0.609 and comparing them with the responses ρ1 and ρ2 for Y33crystal (Figure 4B, green crosses), we obtained the upper and lower bounds of the ring-flip rate constants for Y33crystal [...]"

Moreover, we have added a panel to Figure 4B that shows the relevant responses (previously only shown in the SI). We hope that this clarifies how we get to the upper and lower bounds of the time

scale.

6. On page 8 the manuscript says “We speculate that intermolecular contacts favor more extended states of GB1 by loosening the hydrophobic core.” What is the rationale or reason for this speculation?
We do not have clear mechanistic evidence, because there is no crystal structure of GB1 with full-length IgG complex. We have rewritten this part somewhat to avoid the statement of “extended states”, as follows
“This finding implies that the intermolecular contacts in the complex favor states that facilitate ring flips. One explanation could be that these intermolecular contacts lead to less compact states of GB1 in which the hydrophobic core is less restricted and therefore, the energy barrier for ring flips is lower.”
Our rationale is as follows: the protein’s conformational energy landscape is determined by the interactions – within the protein, to water, and to its binding partner (IgG). Accelerated ring flips, compared to solution, necessarily imply that some of the interactions with the binding partner loosen those contacts that contribute to the ring-flip energy barrier in the monomeric protein. Unfortunately, in the absence of a high-resolution structure, we cannot point to molecular mechanisms.

7. Figure 3 on page 8 shows the EXSY data, showing exchange peaks for both Y3 and Y45. In the figure, the broad Y33 peak overlaps somewhat with several peaks, including peak Y3ab, Y45ba, and Y3b. The caption notes that only peak Y3ab was not analyzed due to ‘peak overlap’, but in the figure the overlap with Y45ba seems similarly bad? I don’t immediately see or understand the reason for only skipping one of the affected peaks.
8. Perhaps related: why is the peak intensity of Y3ab so different from Y3ba? It does not look (to me) like that is fully explained by overlap with the Y33 peak?
As points 7 and 8 are directly related, we answer them together:
We have added the extracted intensities of all peaks in Figure S13 for transparency. The choice to omit peaks from the fit for Y3 only comes from the observation of the extracted intensities. For Y3, there is a clear difference in intensity between Y3ab and Y3ba. Possible explanations could be overlap with Y33, or differences in CP efficiencies or relaxation properties for both sides of the ring. This is why we chose to analyze Y3ba only. However, one can see from the built-up curve of Y3ab and Y3ba that the rate is very similar and that this simplification does not have a significant impact on our results. For Y45, we did not observe such a difference in intensity, which is why we chose a combined fit of all peaks.
We have added the following sentences to clarify this in the manuscript:

“Due to the symmetry of the labeling, the expected populations of “a” and “b” are pa = pb = 0.5. Despite this theoretical value, we observed a difference in the intensities of Y3a and Y3b, and Y3ab and Y3ba (Figure S13), which is why we only fitted peak “ba” for residue Y3. Reasons for this could be partial peak overlap with Y33, or differences in 1H relaxation and CP efficiency between the two sites. For Y45, we performed a combined fit of all four signals.”

9. Page 9: discusses changes in entropy. The text currently focuses this on differences in the entropy of the transition state. But I wonder: is it not also possible that there are also differences in the ‘ground’ state in terms of entropy? I.e., could the entropy of the non-flipping state be higher or lower in order to explain the differences in delta-delta S?
We thank the reviewer for pointing out this issue in our argumentation. It is correct that we cannot make any statement about differences in the entropy of the transition states with our data. In fact, both the ground and the transition state are likely lower in entropy in the crystal than in solution, respectively. What we can say is that the favourable entropic gain in the crystal is lower than in solution, leading to an overall higher free-energy barrier in the crystal than in solution. We have rewritten the sentences in the text and added more explanation to the discussion to clarify this:
p.9: “Ring flips in crystals are less entropically favoured”
“In other words, the entropic gain of the ring flip transition of Y3 and Y45 is lower in crystals than in solution.”
p.14 (discussion): “Our thermodynamic analysis reveals that this is due to a change of the entropic barrier between solution and crystal with 0 < ΔS‡,crystal < ΔS‡,solution (Figure 5C). The positive ΔS‡ in both solution and crystal means that the transition from the ring-flip-incompetent ground state to the ring-flip-competent transition state comes with a gain in entropy. This entropic gain is counteracted by an enthalpic penalty, possibly due to interactions that break to create a loosened transition state, leading to the observed free-energy barriers ΔG‡. The overall free-energy barrier for ring flips is higher in the crystal because the entropic gain ΔS‡,crystal for going from the ring-flip-incompetent state to the ring-flip-compentent states is smaller than in solution.”

10. In the discussion of delta S, and delta H it could be useful to explicitly comment also on the apparent sign. (In the discussion of comparing the differences as larger or smaller)
We added the following on p.9: “We obtain positive values for ΔH‡ and ΔS‡ for both residues in solution and the crystal with a large difference in activation entropy, ΔS‡,solution> ΔS‡,crystal. This observation confirms that the flips of Y3 and Y45 are slowed because there is a reduced entropic

gain for the ring flip in the crystal than in solution."
See also the part added to the discussion in the previous point.

11. Figure 4 on page 9: please explain meaning of dashed line(s) and grey areas in Figure 4A in the caption.
We thank the reviewer for pointing out that this is missing. We added the following to the caption: "The dashed line in the upper panel of A indicates the value of the expected order parameter for a ring flip S = 0.625.71 The order parameter of p3 could not be determined due to too low sensitivity (gray box)."

12. Page 11 talks about the good match between energy barriers in MD and experiments. Please explain exactly what is matching? This is the value of delta-delta G? mention and compare the numbers.
What matches particularly well are the values for ΔΔG. We have added the following comparison: "The values for ΔΔG‡,sim are (13 ± 5) kJ mol−1 for Y3 and (17 ± 4) kJ mol−1 for Y45, virtually the same as the ones obtained experimentally (ΔΔG‡,exp = (15±5) kJ mol−1 for Y3 and (17.9 ± 1.5) kJ mol−1 for Y45)."
While adding this, we also noticed that we made a small mistake when adding the numbers for ΔΔG‡,exp in the initial manuscript. We have now corrected this on p.9.

13. Page 12, Figure 6: The meaning of this figure could be better explained. In panels A-F; what is exactly being shown? What do the pale/dark colors indicate?
14. The caption title suggests comparison to solution state, but if i understand correctly the shown data are only in crystals? Also, how were these frames selected?
Panels A-F of Figure 12 show frames 19400 (left panels) and 23200 (right panels). Frame 19400 represents a frame where Y45 is oriented to the core of GB1 while frame 23200 represents a frame where Y45 is oriented outward. A and B show intramolecular contacts and C-F show intermolecular contacts (from two different orientations). The dark and pale colours show the two chains of the crystal. We added more detail to the caption; it now reads:
"Structural insights into the interaction network of Y45 in crystals from MD simulations.
(A-F) Close-up of Y45 and residues interacting with it intermolecularly (A-B) or in a neighboring molecule (C-F). Interactions are shown for two different frames of the simulations, representing a state in which Y45 is oriented towards the core of GB1 (frame 19400; brown; A,C,E) or outward rotated (frame 23200; gray; B,D,F). Pale and dark shades of brown and gray indicate chains A and B of the crystal, respectively. [...]"

15. Page 13, at the top/left: so the basic idea is that crystal contact forces destabilize the monomer core structure? In this context, it could be interesting to discuss any differeneces in stability in this GB1 variant compare to WT/T2Q.
In this paragraph (now p. 14, left), we discuss the potential influence of intermolecular crystal contacts on the ring flip dynamics. The underlying idea is that these intermolecular contacts restrict the dynamics in the crystal compared to solution, leading to a deceleration of the ring flip rate in the crystal. This might be related to the observed decrease in activation entropy in the crystal.
We think that this effect is caused by the crystal lattice only and that the stability of the protein itself has no influence on this. A different crystal form of the same protein variant might even cause a different behaviour. As we do not have data on the ring flip rates in the T2Q variant in either solution, nor this exact crystal form, we cannot make a definite statement about the influence of protein stability on the observed effect. Note, however, that the comparison of ring flips in solution and in crystals is done here for the same protein (GB1-QDD). The fact that this is a mutant of GB1 is not really relevant.

16. One point of the discussion is about the idea of 'breathing' of the overall fold. Would a lack of correspondence also make sense in prinicple, as the timescale of ring flip would be expected to be quite different from 'breathing' – would the latter not have to be much slower? (Perhaps I am missing something?)
The concept of "breathing motion" is widespread in the literature, but we think that it is not a very well-defined term. We are using it due to its ubiquity in the literature, but our data to not confirm such global motions. However, what is often implied with this terms is that aromatic rings can flip only when such correlated "breathing" motions happen. Our study points to more complex mechanisms of local rearrangement.
We have added the following sentence to the discussion: "We argue that what is implied by the term "breathing motion" -- a term that is not very well defined -- is likely a more complex rearrangement of local interactions, somewhat concerted, but not necessarily involving an overall transient expansion."

17. Page 2: it is stated that differences in the epsilon and delta shifts can help identify exchange dynamics. This could be read as a suggestion that there is exchange from delta to epsilon. I assume it is meant that a particular epsilon H displays distinct shifts depending on the ring flip? Either way this could be written more clearly, especially also for non-experts.
We added the following clarification to the text:

“In contrast, they are easily observable by NMR, since the 1H and 13C spins at the δ and ε positions differ in chemical shift. A ring flip consequently leads to an interchange of (CH)ε1 and (CH)ε2 as well as (CH)δ1 and (CH)δ2, resulting in line broadening or exchange cross-peaks depending on the timescale (Figure 1B).”

18. Page 3, top of right column: the space group identifier is not properly formatted
We corrected the formatting of the space group identifiers.

19. On page 3 of the SI, the process of preparing the samples is discussed. Could one add an estimate of the amount (mg) of protein per sample, and the relative amount of water present? Also, what was the centrifugation used to pack the GB1-IgG complex?
We added estimates of the amount of sample filled into the rotors to the Supporting Information, on page 3. We have ca. 3 mg protein in a 1.3 mm and 12 mg in a 1.9 mm rotor. According to the crystal structure, we know that 46.6% of the crystal volume is water. In addition, it is common that there is some additional “supernatant” solvent in the rotor (see e.g. Figure 1 of Böckmann et al, 10.1007/s10858-009-9374-3). We also added a statement about the centrifugation of the complex at the same location in the SI, which was performed at 68000 rcf over night.

20. Page 12 of the SI: I suggest providing a bit more info on the source of the CSA tensor, and explicitly noting the relevant nucleus. (Question: does the orientation of the CSA relative to the bond direction matter?)
We have added the citations for the sources of the tensor parameters in the SI and also clarified the nuclei.
In general, the relative orientation of the CSA and the dipolar tensor matters. However, it is not trivial to model this, and it is assumed in the detectors analysis that the tensors are colinear. This is a valid assumption for the cases that we are studying here.

21. SI: please expand captions of SI figures S20-S22 to explain in more detail what is shown. (Perhaps this may also aid some of the other SI figures, to make sure that they are sufficiently clear on their own)
We added more explanation to these figure captions.
Reviewer #2 (Comments for the Author):

In my below review of the manuscript „Aromatic Ring Flips Reveal Reshaping of Protein Dynamics in Crystals and Complexes”by Becker and coworkers, I tried to closely follow the instructions and recommendations provided on https://www.nature.com/nature-portfolio/editorial-policies/peer-review

• Key results: Please summarise what you consider to be the outstanding features of the work.
The outstanding features of this work are, in my opinion, the very high-quality of the data and methodological approaches taken, the combination of high-fidelity biomolecular NMR experiments with state-of-the-art MD simulations, and the detailed interpretations of the results. The main results are that the crystal environment affects the protein dynamics (compared to, e.g., the protein in solution or also the protein in a binary complex with another protein binding partner) in a distinct way, as probed by the ring flips or aromatic side chains in the core of the small protein GB1, which serves as a model system in this study.

• Validity: Does the manuscript have flaws which should prohibit its publication? If so, please provide details.
There are no such flaws. The work has been carried out at an extremely high technical level, and the results are clearly and carefully presented, interpreted and discussed, with very strong evidence for the conclusions. The authors are to be commended not only for their impressive level of expertise, but also with the modest tone. We have a few questions, comments, and suggestions for further improvements, see below.

• Originality and significance: If the conclusions are not original, please provide relevant references. On a more subjective note, do you feel that the results presented are of immediate interest to many people in your own discipline, and/or to people from several disciplines?
This is of course not the first study that compares the dynamics of proteins in solution vs. in crystals. However, it stands out in terms of its technical quality and especially also in terms of the great level of detail, down to the atomic level.
I think that it is hard to judge whether it provides an “advance in understanding likely to influence thinking in the field”. In general, the impact of the environment on the conformational ensembles and also on the dynamics of the structural transitions in proteins is still underappreciated (and sometimes even overlooked). Thus, I think that the present work is of significant interest.

• Data & methodology: Please comment on the validity of the approach, quality of the data and quality of presentation.
All outstanding, see above.

• Please note that we expect our reviewers to review all data, including any extended data and supplementary information. Is the reporting of data and methodology sufficiently detailed and transparent to enable reproducing the results?
Yes.

• Appropriate use of statistics and treatment of uncertainties: All error bars should be defined in the corresponding figure legends; please comment if that's not the case. Please include in your report a specific comment on the appropriateness of any statistical tests, and the accuracy of the description of any error bars and probability values.
Appropriate statistical uncertainties/error estimates are reported and explained in the SI, but are not always defined in the figure legends. That could be easily added.
We thank the reviewer for pointing out this issue. We have now added statements explaining the error bars in the figure legends.

• Conclusions: Do you find that the conclusions and data interpretation are robust, valid and reliable?
Absolutely yes.

• Suggested improvements: Please list additional experiments or data that could help strengthening the work in a revision.

1. For a meaningful comparison of the dynamics of different systems (e.g., GB1 QDD vs. T2Q crystals, or GB1 crystal vs. IgG:GB1 complex), it is of course essential to consider them at the same temperature. Can the authors explicitly refer to that in the text (for example, but not necessarily only limited to, the data in Figure 2 and the corresponding discussion -- I consulted the two PhD theses from U Warwick (Refs 58 and 60) but could not quickly find the relevant information...)?
We thank the reviewer for pointing out that this information is missing in the manuscript. We added the temperatures for the different datasets to the caption of Figure 2. The data for GB1QDD were obtained at 304 K, while the other two datasets were obtained at 300 K. This temperature difference is negligible for our qualitative comparison of the dynamics in these systems.
See also ref. 59, p.177.

2. In the text, the authors equate entropy with "disorder". Interpreting entropy as "disorder" is a simple and often applied analogy but can in many cases be misleading (also here, in our opinion). A smaller number of accessible microstates in a reduced/compressed configuration space volume might be a better way to think about entropy in this context.
We thank the reviewer for this suggestion. We have rewritten the discussion about the entropic contribution (p.11, top left and p. 15, left column) to clarify these possibly confusing notations. In particular, we have also changed our wording and included the concept that there are fewer microstates accessible, which is certainly a more precise way of expressing this, compared to just equating entropy with "disorder". We thank the reviewer for this suggestion.

3. On p. 9 (right column), the authors conclude that "the transition states of the ring flips of Y3 and Y45 in crystals are less disordered compared to those in solution." However, that is not necessarily so. Couldn't it also be that the entropy of the ring flip transition state ensembles are the same in the Xtal and in solution, but that the initial state (the "educt minimum") is more ordered in the Xtal than in solution?
We agree with the reviewer that our statement about the entropy of the transition state was not accurate. We clarified this in the text and added a more detailed explanation in the discussion, see page 14, right column. Please also refer to our answer to point 9 of reviewer 1.

4. Further down in the same paragraph, they write that "A lower degree of disorder in the crystal is expected as the crystal lattice imposes constraints on the protein." That statement is of course true, but in what way is this related to (or relevant for) the discussion of the larger dS of activation in the crystal? Because the "lower degree of disorder" of the Xtal compared to the solution could, in principle, equally apply to both the equilibrium state (the minimum) and the transition state. In that case, the difference (Delta) would be the same as in solution.
We thank the reviewer for this comment and have removed this sentence on p.9 (now p.11). With the data at hand, we can only make statements about the barrier height in each system and not about the entropy differences between states in different systems.

5. I am puzzled by Figure 5C. Purple and green colors denote the data in the Xtal and in solution (at least that color code was used in the previous figures, but unfortunately also used in Fig. 5B in a different context). Then why isn't the dS of activation in the Xtal plotted in panel C, but only -TdS in solution?? As far as I understand, this information should be available from the fit shown in Figure 5B (which was done by constraining dH of activation in solution to be the same as dH of activation in the Xtal, as is written in the figure caption -- can the authors justify this assumption?).
The color code for solution and crystal is purple and green, respectively. In Figure 5B, two other colors were introduced to distinguish the two residues. We changed this to different symbols and line styles to keep the color code fully consistent throughout the manuscript. In Figure 5C, we are now

plotting TΔS instead of -TΔS, which might have been misleading, and added labels to the columns. Indeed, TΔS is available for solution (purple) and crystal (green), and is plotted for both. The values for the crystal are very small, but we hope that the added labels help to spot them.

Regarding the justification of our assumption of equal ΔH in solution and in crystals, our rationale is the following:

- The slopes in Figure 5C indicate that ΔH(solution) ≥ ΔH(crystal), as justified by the fact that the slopes are similar: an equal slope would imply that ΔH(solution) = ΔH(crystal), while a smaller slope in the crystal would imply ΔH(solution) ≥ ΔH(crystal). See the discussion on page 9 (right column) and around Figure S9.
- We have also tested fits in which ΔH and ΔS are fitted independently, imposing only that ΔH(solution)≥ΔH(crystal) and ΔS(solution)≥ ΔS(crystal); these assumptions naturally follow from the slopes, as discussed above. While such a fit is not ideal, as we fit two parameters from two observables, the resulting fit shows at least qualitatively the same conclusion, namely that ΔS(solution)>ΔS(crystal). Please find the fitted values below:

6. Another puzzling aspect of Figure 5C is that -TdS of activation is negative, that is, the entropic contribution to the activation free energy barrier is favorable. How can that be reconciled with the transition state having "reduced disorder" (= lower entropy)?
We apologize for the somewhat confusing manner in which we presented the data in the previous version. We are now plotting TΔS in Figure 5C and have changed the caption of that paragraph to "Ring flips in crystals are less entropically favoured".

7. Can the authors comment on why the energy profiles in Figure 5D are skewed (the green and purple curves for Y3 in the left panel and the purple curve (solution data) for Y45 in the middle panel)?
There are a number of physical reasons why the free-energy profile does not need to be fully symmetrical. The first lies in the functional form of the dihedral angles in the force field. Most academic classical force fields, in general, and the CHARMM force field, in particular, use a sum of cosine terms with different multiplicities and phases, as opposed to a single simple $U(\varphi)=U_0(1+\cos(n\varphi-\varphi_0))$. This superposition of terms easily produces asymmetric wells and skewed barrier shapes. The second lies in the coupling to other degrees of freedom. The dihedral angle rarely moves alone. Valence angles and neighboring dihedral angles along the side chain, together with backbone adjustments, change with φ, and have different densities of states on either side. This can be viewed as a state-dependent entropy contribution, likely to introduce skewness to the free energy. A third reason is rooted in the nonbonded interactions and the stereochemistry, with possible steric clashes, gauche versus trans interactions, and transient intramolecular hydrogen bonds which can result in one flank of a well steeper or shallower than the other. Fourth, in explicit solvent, orientation of certain polar groups relative to the solvent, as well as solvation shells, and ion-binding sites can promote asymmetric stabilization for certain values of φ. Fifth and last, in connection with the second argument, entropic contributions necessarily vary with φ. Even if the mean potential energy were symmetric, a φ-dependent entropy makes the free energy along φ asymmetric. Narrow, deep wells have lower entropy than broad, shallow wells. Skewness or asymmetry in the free-energy profile reflects a variation in the entropy term across the collective variable, φ. Deep wells tend to be associated to larger entropy gradients.
We have added a statement to the figure legend to address this topic.

8. The authors should compute also dH and dS of activation (for example, from a series of free energy simulations at different T). That would be important to confirm the interpretations of the experimental data in terms of increased activation entropy (but not activation energy) in the Xtal compared to in solution.
While estimation of ΔS and ΔH by means of computer simulations would be desirable, especially in the present case, the resulting estimates are likely to be burdened by very large errors, making them in general meaningless. To compute the entropy using geometric free-energy calculations, as we did for the isomerization of the aromatic side chains, we often turn to the relationship $\Delta S = -\partial\Delta A/\partial T$, at constant N and V, which we handle in terms of finite differences, that is, $\Delta S(T) = -[\Delta A(T + \Delta T) - \Delta A(T - \Delta T)] / 2\Delta T$. The choice of ΔT is, however, far from trivial. If ΔT is too small, then the difference $\Delta A(T + \Delta T) - \Delta A(T - \Delta T)$ will be on the order of the uncertainty in the free-energy estimates. Conversely, if ΔT is too large, the finite-difference approximation is no longer valid.

Even with extremely long simulations, obtaining a reliable estimate of the entropy for the system at hand is essentially infeasible. First, the true uncertainty in our free-energy calculations is almost surely much greater than the statistical error bars reported in the manuscript, since the degree to which the simulations have explored the orthogonal degrees of freedom—specifically, the slow modes that are coupled to χ_2—is largely indeterminate. Even with an ergodic-sampling strategy consisting of multiple walkers exploring the collective variable concomitantly and exchanging information on the gradients measured locally, overcoming hidden barriers in the orthogonal manifold remains computationally challenging. Expecting to resolve subtle temperature-dependent variations in this orthogonal sampling within a multi-µs, albeit finite simulation window, and for such effects to be reflected accurately in the free-energy profile at sub-kcal/mol precision, is, therefore, unrealistic.

To substantiate our reluctance to undertake the suggested additional simulations, we refer the Reviewer to calculations published in Accounts of Chemical Research (Fu et al. Acc. Chem. Res. 2019, 52, 3254–3264) for two objectively simpler systems—ethanol hydration and the binding of a proline-rich peptide (p41) to the SH3 domain of the Abl kinase—where sampling in the orthogonal manifold is only a minor concern,
although the entropy estimates themselves appear to be reasonable, and agree fairly well with the experimental values, the very large associated uncertainties, greater than the actual values of the TΔS terms, remain problematic and inevitably cast doubt on the reliability of the results. While it is possible that our TΔS estimates are adequately converged, the magnitude of the error bars makes it difficult to assert it with confidence.

9. On p. 8 (right column), the authors write that “intermolecular contacts favor more extended states of GB1 by loosening the hydrophobic core”. Do they mean more “expanded states" or "less compact states" maybe? "Extended" implies elongated, which might not be exactly what the authors want to say here?
We have rewritten that sentence for clarification. Please refer to point 6 of reviewer 1 for a more detailed answer.

• References: Does this manuscript reference previous literature appropriately? If not, what references should be included or excluded?
References are appropriate.

• Clarity and context: Is the abstract clear, accessible? Are abstract, introduction and conclusions appropriate?
Yes.
Reviewer #3 (Comments for the Author):
The manuscript by Becker, et al reports an NMR study of aromatic ring flipping kinetics in a crystallized protein (GB1), providing a comparison to the behavior of the protein in solution as a monomer and in complex with a binding partner (IgG). The authors implement a clever metabolic labeling methodology that incorporates 13C into the Cε positions of Phe and Tyr residues, which enables relatively straightforward measurement of ring flipping kinetics over a range of timescales using a battery of different NMR relaxation experiments. The results show that ring flipping kinetics are slowed significantly when the protein is in the crystalline phase relative to wen in solution, and significantly accelerated when the protein is in solution as a heterodimeric complex. A classical Eyring analysis shows that when the protein is crystallized, there is a large decrease in the entropy of activation for ring flips, which results in the observed reduction of the rate constant. By extension, the authors propose that in the GB1:IgG complex, GB1 is trapped in an “extended” conformation that lowers the activation energy for ring flipping. Analysis of MD simulations of the protein in solution and in the crystal provide additional insight into how the aromatic ring flips are coupled to the global dynamic of the protein in both phases, and illustrate how the internal dynamics are perturbed by the crystal lattice.

All the experiments and analyses in the paper appear to have been performed rigorously, and I have no major technical concerns with the manuscript. I also would like to mention that the introduction to this paper is very well written and was interesting to read. Overall, I think this paper is an excellent demonstration of how a protein’s dynamics can be perturbed by its environment. There is a clear cautionary message for experimenters who use crystallographic approaches to study protein motions, but the results also provide additional evidence that molecular interactions, broadly speaking, can influence the internal dynamics of proteins, which is a fundamentally important concept in biophysics. These insights will be of interest to the Nature Chemistry audience, which includes structural biologists and biophysicists, and I recommend publication with minor revisions. In particular, I note that Nature Chemistry has a strong track record of publishing high-impact papers in the field of time-resolved crystallography, and this manuscript is especially relevant to this audience.

I have only one major technical concern. The crystal structure reported by the authors, which is subsequently used for a B-factor analysis and for launching MD simulations was determined at cryogenic temperatures. Looking at the unit cell parameter calculated for the single crystal (cryo) and powder diffraction (room temp) experiments shows a significant contraction of the unit cell due to

cryocooling, and I wonder whether this could affect the interpretation. Given that single crystal experiments are trivial at room temperature, and that the authors have microcrystal in hand that could facilitate serial data collection, I wonder why a room temperature crystal structure was not used for the analysis?

We thank the reviewer for this comment. For this revised version, we determined a single crystal X-ray structure at 295 K (deposited as PDB 9T8Z). However, we believe that the cryo-temperature crystal structure is still a valid, and arguably better, starting point for the MD simulations and for mechanistic considerations, for the following reasons.
The reservoir buffer for the crystallization contains 25% isopropanol, which is volatile, and we found that the crystals were very fragile at room temperature due to isopropanol evaporation and dehydration. While for NMR rotors this is not a problem – the rotors are closed tightly to avoid any evaporation – it can cause problems for crystallography. This was less of a problem for the structure obtained at cryogenic conditions. Consequently, the resolution of the room temperature structure is slightly lower. There might also be some structural alterations, possibly, due to evaporation; we see a weak density that might indicate a low-populated conformation of Y33, for example (at 0.7 σ).
Therefore, it seems safer to use the 100 K structure for the analyses and as outlined below, the relaxed MD structure reflects the physical situation very well, in our opinion.

Besides possible complications with solvent evaporation, the newly determined room-temperature structure is useful in the sense that it shows the expected trends in terms of unit cell size. We find the following values (a, b, c, β), which are compared here also to other values we obtained in this study:

- SC XRD, 100 K: 77.48 Å, 35.18 Å, 49.94 Å, 122.51°
- SC XRD, 295 K: 78.70 Å, 35.41 Å, 51.82 Å, 122.60°
- Powder XRD: 77.9 Å, 35.3 Å, 51.4 Å, 122.3°
- MD after energy minimization: 80.36 Å, 36.49 Å, 53.24 Å, 120.71°

The thermal expansion is in line with the expectation (https://doi.org/10.1021/bi00375a035).

Importantly, in the equilibration simulations for MD simulation, which used the 100 K crystal structure as a starting point, the simulated lattice reached unit cell parameters that are close to those found in the 295 K structure, suggesting that our simulations are a good proxy for the behavior at ambient temperature. For these reasons, having collected now both a 100 K and a 295 K structure, we believe that the MD simulations that use the former are valid.

I also have the following minor suggestions for the authors:

• The manuscript focuses on the kinetics of ring flipping, but I wonder whether the NMR data also contain information about the equilibrium populations of different ring conformations for GB1 in solution and in the crystal? While the differences in kinetics are important, especially with respect to time-resolved crystallography, differences in equilibrium populations are arguably more important, because the occupancies of alternative conformations are commonly refined in static crystallography experiments as well (especially at room temperature). Static crystallography experiments are far more common, and this additional insight could increase the impact of the paper if the authors can provide data and commentary.
A ring flip is a symmetric motion in which the two states cannot be distinguished structurally (see also Figure 1A), and the populations of the two states are always 50 %. The strength of NMR in this case lies in its ability to study the dynamics of this process, even though the two states are structurally indistinguishable. This is because the chemical environment of (CH)ε1 and (CH)ε2 is not identical, resulting in unique chemical shifts for the two sites.
For non-symmetric processes, it would be possible to obtain information about the populations of different states by NMR. However, none were observed, both in solution and in crystals.
• I appreciate that many authors are reluctant to speculate beyond the data reported in their manuscript, but I think it could be useful to discuss how these results might extrapolate to other systems. For example, will the crystal lattice always restrict dynamics? Based on the findings reported for the GB1:IgG complex, could there be situations where a protein crystalizes in a conformation that accelerates internal dynamics? I want to emphasize that I am not suggesting any additional experiments on other systems, but some mention of how widespread the authors expect this phenomenon to be would be useful.
We thank the reviewer for this suggestion. Proteins are incredibly versatile and complex molecules

that are, by design, very sensitive to interactions with other molecules and their surroundings. We imagine that the effects of a crystal lattice on the dynamics of the protein can be just as diverse. The exact characteristics of these effects most likely strongly depends on factors like the density of the packing, the solvent contents and the amount of specific intermolecular crystal contacts. In our opinion, it is conceivable that crystal packing could also lead to an acceleration of internal dynamics, e.g. by stabilizing such states in which ring flips are facilitated. Even though this is of course highly speculative, it would not change the message of our work: one should be careful to make conclusions about protein dynamics measured in such an unnatural environment as a crystal.
We have added the following statement to the discussion (p.15-16):
"It is tempting to extrapolate our findings on GB1 to other crystalline systems. Since crystalline environments are inherently more densely packed than the protein's solution environment, ring flips in many crystals are likely slower than in solution. However, the specifics may depend heavily on context, as demonstrated by our IgG:GB1 results. If intermolecular contacts favor conformations that facilitate ring flips, the crystalline environment might even accelerate them. Even for a single protein in different crystal lattices, ring flip dynamics may vary, just like overall rocking motion, which also depends on packing density.35"

• Figure 2B could be improved by performing a B-factor normalization. Clearly, the overall B-factor is much higher for the GB1QDD than for the GB1T2Q crystal. Normalization could make these data more easily comparable across different crystal forms. See, for example Barthels, et al. (2020) Mol. Inf. and/or Carugo (2022) Acta Cryst. D.
We used the tool by Barthels et al. (2020) to normalize the B-factors in Figure 2B. The raw B-factors can now be found in Figure S2B. We thank the reviewer for this suggestion.

Additional changes
We have further made the following minor changes to the manuscript.

- We have added an author, Daniel M. Balazs, who contributed to the crystallographic work. This change reflects the increased fraction of the crystallographic data in this revised version.
- We have deposited the NMR assignment of GB1QDD at the BMRB under the access code 53330. During the deposition, we discovered a mistake in the referencing of the 13C dimensions in the assignment (shift by 2.66 ppm), which we corrected in the respective parts of the manuscript.
- We have added a Data Availability statement to the manuscript.

Version 1:

Author Rebuttal letter:

Reviewer #1:
Comments for the Author:
I thank the authors for their thoughtful responses to the comments from myself and the other two reviewers. The raised questions and comments have been addressed and/or improved in the revised manuscript. I have no significant open questions left.

Except for one small point that could be made clearer, in my view. In my point #17 we discussed the shift differences of the epsilon and delta sites. The revised text is improved. It does still retain the sentence "In contrast, they are easily observable by NMR, since the 1 H and 13 C spins at the δ and ε positions differ in chemical shift. " I still worry that people may read this incorrectly, as emphasizing that the shift of the epsilon site differs from that of the delta site. Naturally these shifts are indeed different, but the point here is rather that CE1 and CE2 differ from each other, not that they differ from CD1/CD2.... A rephrasing of the retained sentence could try to avoid this possible misreading?

We thank the reviewer for their positive feedback.
We edited the sentence in the following way and hope that this is clearer now:
"In contrast, they are easily observable by NMR, since the 1H and 13C spins on each side of the aromatic ring (e.g. ε1 and ε2) differ in chemical shift."

Reviewer #2:
Comments for the Author:
We are satisfied with the changes made. It is especially nice to see that the authors determined an Xray structure also at room temperature. The authors are to be commended for their careful work and their conclusive and important results.

We thank the reviewer for their positive feedback.

Reviewer #3:
Comments for the Author:
I am satisfied that the revised version of the manuscript and the rebuttal from the authors adequately address my concerns. I appreciate the challenges related to crystallization conditions involving volatile solvents, and I apologize for my oversight regarding the symmetry of the ring flips as it pertains to equilibrium populations.

We thank the reviewer for their positive feedback.

Open Access This Peer Review File is licensed under a Creative Commons Attribution 4.0 International License, which permits use, sharing, adaptation, distribution and reproduction in any medium or format, as long as you give appropriate credit to the original author(s) and the source, provide a link to the Creative Commons license, and indicate if changes were made.
In cases where reviewers are anonymous, credit should be given to 'Anonymous Referee' and the source.
The images or other third party material in this Peer Review File are included in the article's Creative Commons license, unless indicated otherwise in a credit line to the material. If material is not included in the article's Creative Commons license and your intended use is not permitted by statutory regulation or exceeds the permitted use, you will need to obtain permission directly from the copyright holder.
To view a copy of this license, visit https://creativecommons.org/licenses/by/4.0/

Reviewer #1 (Comments for the Author):

1. The GB1 variant is quite interesting. Are there any data on any difference in stability (e.g., melting temperature) compared to T2Q and WT GB1? This could be useful to discuss, especially in context of the proposed 'distortion' of the GB1 fold.

The melting temperatures T_m of GB1 T2Q and QDD at pH 5.4 (maximum stability) are 87.5 °C and 74 °C, respectively (Lindman et al., 2006 https://doi.org/10.1529/biophysj.105.071050). We have measured melting curves for these two variants at the conditions used in the manuscript (50 mM NaP, pH 6.5). We find an overall similar conclusion, namely that the T2Q variant is slightly more stable than QDD. At the conditions of this study, QDD is fully folded and stable.

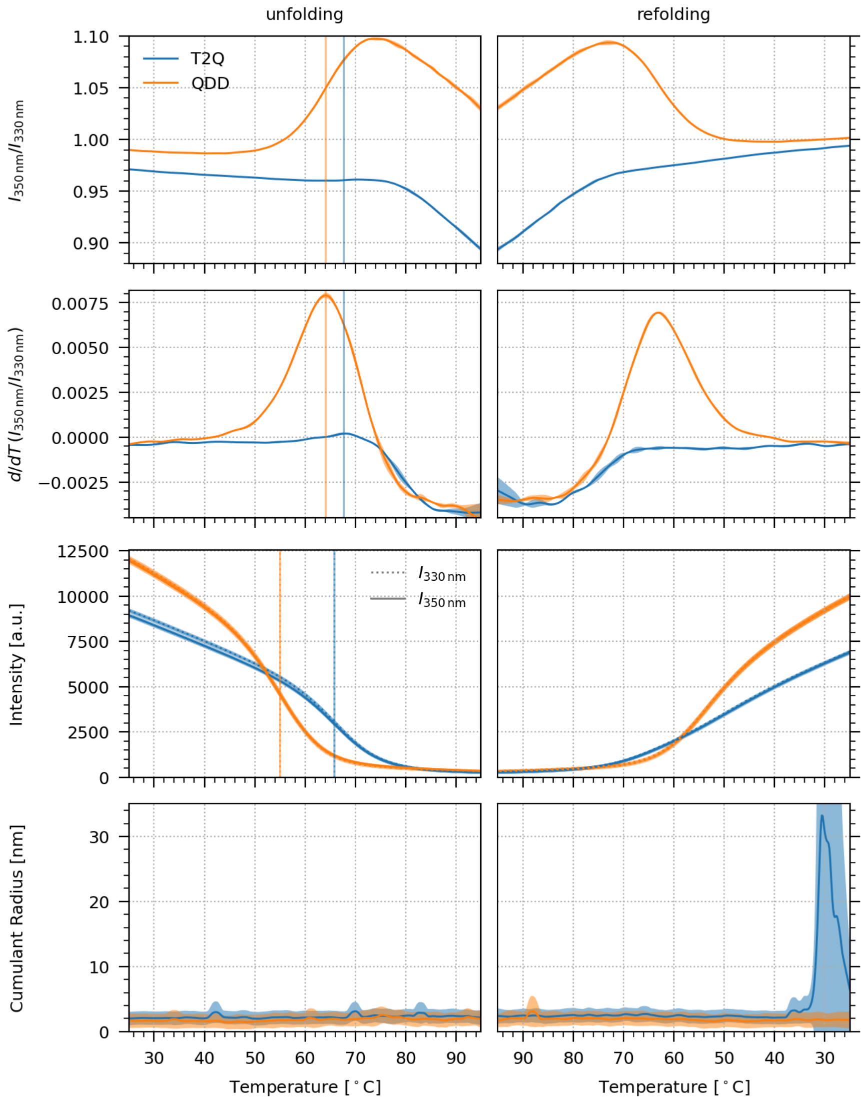

Unfolding (left) and refolding (right) curves for GB1 T2Q (blue) and QDD (orange) measured and analyzed with the nanotemper Prometheus Panta. Curves were measured between 25 and 95 °C in steps of 1 °C/min with protein concentrations of 1.3 and 1.6 mg/mL for T2Q and QDD, respectively.

The first and the second rows show the intensity ratios between 350 nm and 330 nm, and their derivatives, respectively. Vertical lines indicate the inflection points (IP).

The third row shows the individual intensity curves measured at 330 nm (dotted) and 350 nm (solid line). Vertical lines indicate the inflection points.

The last row shows the cumulant radius.

method	IP T2Q [°C]	IP QDD [°C]
(I_{350nm}/I_{330nm})	67.70 ± 0.42	64.05 ± 0.13
I_{330nm}	65.82 ± 0.04	54.96 ± 0.08
I_{350nm}	65.76 ± 0.04	54.97 ± 0.09

In our manuscript, we discuss the differences between different forms (solution, crystal, complex) of GB1 QDD, and not between different variants of GB1. We therefore don't find this data relevant in the context of our manuscript and prefer not to discuss it.

2. Moreover, the manuscript describes the modeling of the GB1-IgG interaction based on the WT mode of binding. It would be useful to discuss briefly the positioning of the introduced D amino acids relative to the binding interface. Is there a chance that it may be affected?

We thank the reviewer for this suggestion. Given that there are no crystal structures of full-length IgG with any GB1 variants, the model we propose is indeed somewhat speculative: it is based on the crystal structures of GC2 or GB3 with IgG fragments. There are no crystal structures (even of IgG fragments) with the T2Q or QDD GB1 variants. We cannot assess whether the introduced mutations change the binding mode.

With these limitations in mind, we propose that the three mutations of the QDD variant have only limited (if any) impact on the binding: two of the three mutation sites are quite distant from the binding interface. The mutation that is closest to the interface (N37D) introduces a rather small change (Asn to Asp); its closest neighbor on the IgG side is a Ser, such that it is unlikely that the interaction gets significantly affected by the N37D mutation.

To make it easier for the reader to locate the mutation sites, we have indicated them on the structure in Figure 1F and made a note in the figure caption.

Note that the message of the paper does not really depend on the structural details of the IgG:GB1 complex, as we use this complex only to highlight the interesting finding that ring flips are accelerated, without performing an actual structural analysis of the origins.

3. A very useful feature of the paper is the attempt to verify the new (single crystal) X-ray structure with the microcrystals used for the NMR analysis. In that context the manuscript notes that "we performed powder XRD-like measurements on wet microcrystalline samples closely resembling those used for NMR,". What does this mean exactly? How was it established that they 'closely resemble"? (if not by X-ray)

The samples for NMR and powder X-ray diffraction were obtained not only in similar but identical conditions. Our original formulation was misleading and we have now corrected these sentences (page 4, right column, top third) to clarify this.

4. On page 7, the manuscript mentions the order parameter expected for a ring flipping scenario. But it does not explain how this parameter is determined and what the expected value is. Please add this information and quantitatively compare all of the measured S values to this expected number for ring flip.

The values of the order parameter and asymmetry were originally obtained by a numerical procedure, i.e. by explicit simulations of REDOR recoupling curves in the presence of explicit 120° jumps, in the publication by Gauto et al. (reference 72). In brief, the REDOR curve was numerically calculated with the jump model, and then it was fitted with REDOR simulations in which the order parameter and asymmetry are fit parameters. The values of S=0.625 and eta=0.6 were obtained with a chi-square fitting procedure there.

We have added a more explicit statement now as follows (page 7):

"Gauto et al. modeled the motional averaging due to a ring flip with simulations, and determined results in a theoretical value of S = 0.625;[71] reductions below this value point to additional motions, such as the libration of the ring within each rotamer state, motion of the backbone, or the ring axis (i.e., the $\chi 1$ angle).

[...]
In contrast, the order parameters of Y33 and the phenylalanines are lower than the expected value for a ring flip (approximately 0.3 and 0.5, respectively), indicating that these rings flip faster than roughly one hundred μs."

5. At the end of page 7 (and top of next page), the manuscript talks about upper and lower bounds of the rate constants (with reference to Figure 4 and the SI). To me, this explanation was too brief and not entirely clear.

We added more explanation in the main text, as follows:
"The expected response of a dynamic process that occurs on a timescale corresponding to the maximum of a detector sensitivity is $(1 - S^2)$. The responses that we obtain are clearly lower than the expected value for a ring flip, which is $(1 - S^2) = 0.609$ (Figure 4B, lower left panel). With the assumption that rings do not flip faster than hundreds of microseconds and the information from the order parameters, we conclude that Y33crystal flips on a timescale corresponding to the blind spot between $\rho_1(\tau)$ and $\rho_2(\tau)$ (Figure 4B, blue rectangle). By scaling $\rho_1(\tau)$ and $\rho_2(\tau)$ with $(1 - S^2) = 0.609$ and comparing them with the responses ρ_1 and ρ_2 for $Y33^{crystal}$ (Figure 4B, green crosses), we obtained the upper and lower bounds of the ring-flip rate constants for $Y33^{crystal}$ [...]"

Moreover, we have added a panel to Figure 4B that shows the relevant responses (previously only shown in the SI). We hope that this clarifies how we get to the upper and lower bounds of the time scale.

6. On page 8 the manuscript says "We speculate that intermolecular contacts favor more extended states of GB1 by loosening the hydrophobic core." What is the rationale or reason for this speculation?
We do not have clear mechanistic evidence, because there is no crystal structure of GB1 with full-length IgG complex. We have rewritten this part somewhat to avoid the statement of "extended states", as follows
"This finding implies that the intermolecular contacts in the complex favor states that facilitate ring flips. One explanation could be that these intermolecular contacts lead to less compact states of GB1 in which the hydrophobic core is less restricted and therefore, the energy barrier for ring flips is lower."
Our rationale is as follows: the protein's conformational energy landscape is determined by the interactions – within the protein, to water, and to its binding partner (IgG). Accelerated ring flips, compared to solution, necessarily imply that some of the interactions with the binding partner loosen those contacts that contribute to the ring-flip energy barrier in the monomeric protein. Unfortunately, in the absence of a high-resolution structure, we cannot point to molecular mechanisms.

7. Figure 3 on page 8 shows the EXSY data, showing exchange peaks for both Y3 and Y45. In the figure, the broad Y33 peak overlaps somewhat with several peaks, including peak Y3ab, Y45ba, and Y3b. The caption notes that only peak Y3ab was not analyzed due to 'peak overlap', but in the figure the overlap with Y45ba seems similarly bad? I don't immediately see or understand the reason for only skipping one of the affected peaks.
8. Perhaps related: why is the peak intensity of Y3ab so different from Y3ba? It does not look (to me) like that is fully explained by overlap with the Y33 peak?

As points 7 and 8 are directly related, we answer them together:
We have added the extracted intensities of all peaks in Figure S13 for transparency. The choice to omit peaks from the fit for Y3 only comes from the observation of the extracted intensities. For Y3, there is a clear difference in intensity between Y3ab and Y3ba. Possible explanations could be overlap with Y33, or differences in CP efficiencies or relaxation properties for both sides of the ring. This is why we chose to analyze Y3ba only. However, one can see from the built-up curve of Y3ab and Y3ba that the rate is very similar and that this simplification does not have a significant impact on our results. For Y45, we did not observe such a difference in intensity, which is why we chose a combined fit of all peaks.
We have added the following sentences to clarify this in the manuscript:

"Due to the symmetry of the labeling, the expected populations of "a" and "b" are $p_a = p_b = 0.5$. Despite this theoretical value, we observed a difference in the intensities of Y3a and Y3b, and Y3ab and Y3ba (Figure S13), which is why we only fitted peak "ba" for residue Y3. Reasons for this could be partial peak overlap with Y33, or differences in 1H relaxation and CP efficiency between the two sites. For Y45, we performed a combined fit of all four signals."

9. Page 9: discusses changes in entropy. The text currently focuses this on differences in the entropy of the transition state. But I wonder: is it not also possible that there are also differences in the 'ground' state in terms of entropy? I.e., could the entropy of the non-flipping state be higher or lower in order to explain the differences in delta-delta S?
We thank the reviewer for pointing out this issue in our argumentation. It is correct that we cannot make any statement about differences in the entropy of the transition states with our data. In fact, both the ground and the transition state are likely lower in entropy in the crystal than in solution, respectively. What we can say is that the favourable entropic gain in the crystal is lower than in solution, leading to an overall higher free-energy barrier in the crystal than in solution. We have rewritten the sentences in the text and added more explanation to the discussion to clarify this:
p.9: "Ring flips in crystals are less entropically favoured"
"In other words, the entropic gain of the ring flip transition of Y3 and Y45 is lower in crystals than in solution."
p.14 (discussion): "Our thermodynamic analysis reveals that this is due to a change of the entropic barrier between solution and crystal with $0 < \Delta S^{\ddagger,crystal} < \Delta S^{\ddagger,solution}$ (Figure 5C). The positive $\Delta S^{\ddagger}$ in both solution and crystal means that the transition from the ring-flip-incompetent ground state to the ring-flip-competent transition state comes with a gain in entropy. This entropic gain is counteracted by an enthalpic penalty, possibly due to interactions that break to create a loosened transition state, leading to the observed free-energy barriers $\Delta G^{\ddagger}$. The overall free-energy barrier for ring flips is higher in the crystal because the entropic gain $\Delta S^{\ddagger,crystal}$ for going from the ring-flip-incompetent state to the ring-flip-compentent states is smaller than in solution."

10. In the discussion of delta S, and delta H it could be useful to explicitly comment also on the apparent sign. (In the discussion of comparing the differences as larger or smaller)
We added the following on p.9: "We obtain positive values for $\Delta H^{\ddagger}$ and $\Delta S^{\ddagger}$ for both residues in solution and the crystal with a large difference in activation entropy, $\Delta S^{\ddagger,solution} > \Delta S^{\ddagger,crystal}$. This observation confirms that the flips of Y3 and Y45 are slowed because there is a reduced entropic gain for the ring flip in the crystal than in solution."

See also the part added to the discussion in the previous point.

11. Figure 4 on page 9: please explain meaning of dashed line(s) and grey areas in Figure 4A in the caption.

We thank the reviewer for pointing out that this is missing. We added the following to the caption: "The dashed line in the upper panel of A indicates the value of the expected order parameter for a ring flip S = 0.625.[71] The order parameter of p3 could not be determined due to too low sensitivity (gray box)."

12. Page 11 talks about the good match between energy barriers in MD and experiments. Please explain exactly what is matching? This is the value of delta-delta G? mention and compare the numbers.

What matches particularly well are the values for $\Delta\Delta G$. We have added the following comparison: "The values for $\Delta\Delta G^{‡,sim}$ are (13 ± 5) kJ mol^{-1} for Y3 and (17 ± 4) kJ mol^{-1} for Y45, virtually the same as the ones obtained experimentally ($\Delta\Delta G^{‡,exp}$ = (15±5) kJ mol^{-1} for Y3 and (17.9 ± 1.5) kJ mol^{-1} for Y45)."

While adding this, we also noticed that we made a small mistake when adding the numbers for $\Delta\Delta G^{‡,exp}$ in the initial manuscript. We have now corrected this on p.9.

13. Page 12, Figure 6: The meaning of this figure could be better explained. In panels A-F; what is exactly being shown? What do the pale/dark colors indicate?

14. The caption title suggests comparison to solution state, but if i understand correctly the shown data are only in crystals? Also, how were these frames selected?

Panels A-F of Figure 12 show frames 19400 (left panels) and 23200 (right panels). Frame 19400 represents a frame where Y45 is oriented to the core of GB1 while frame 23200 represents a frame where Y45 is oriented outward. A and B show intramolecular contacts and C-F show intermolecular contacts (from two different orientations). The dark and pale colours show the two chains of the crystal. We added more detail to the caption; it now reads:

"Structural insights into the interaction network of Y45 in crystals from MD simulations.

(A-F) Close-up of Y45 and residues interacting with it intermolecularly (A-B) or in a neighboring molecule (C-F). Interactions are shown for two different frames of the simulations, representing a state in which Y45 is oriented towards the core of GB1 (frame 19400; brown; A,C,E) or outward rotated (frame 23200; gray; B,D,F). Pale and dark shades of brown and gray indicate chains A and B of the crystal, respectively. [...]"

15. Page 13, at the top/left: so the basic idea is that crystal contact forces destabilize the monomer core structure? In this context, it could be interesting to discuss any differeneces in stability in this GB1 variant compare to WT/T2Q.

In this paragraph (now p. 14, left), we discuss the potential influence of intermolecular crystal contacts on the ring flip dynamics. The underlying idea is that these intermolecular contacts restrict the dynamics in the crystal compared to solution, leading to a deceleration of the ring flip rate in the crystal. This might be related to the observed decrease in activation entropy in the crystal.

We think that this effect is caused by the crystal lattice only and that the stability of the protein itself has no influence on this. A different crystal form of the same protein variant might even cause a different behaviour. As we do not have data on the ring flip rates in the T2Q variant in either solution, nor this exact crystal form, we cannot make a definite statement about the influence of protein

stability on the observed effect. Note, however, that the comparison of ring flips in solution and in crystals is done here for the same protein (GB1-QDD). The fact that this is a mutant of GB1 is not really relevant.

16. One point of the discussion is about the idea of 'breathing' of the overall fold. Would a lack of correspondence also make sense in prinicple, as the timescale of ring flip would be expected to be quite different from 'breathing' – would the latter not have to be much slower? (Perhaps I am missing something?)

The concept of "breathing motion" is widespread in the literature, but we think that it is not a very well-defined term. We are using it due to its ubiquity in the literature, but our data to not confirm such global motions. However, what is often implied with this terms is that aromatic rings can flip only when such correlated "breathing" motions happen. Our study points to more complex mechanisms of local rearrangement.

We have added the following sentence to the discussion: "We argue that what is implied by the term "breathing motion" -- a term that is not very well defined -- is likely a more complex rearrangement of local interactions, somewhat concerted, but not necessarily involving an overall transient expansion."

17. Page 2: it is stated that differences in the epsilon and delta shifts can help identify exchange dynamics. This could be read as a suggestion that there is exchange from delta to epsilon. I assume it is meant that a particular epsilon H displays distinct shifts depending on the ring flip? Either way this could be written more clearly, especially also for non-experts.

We added the following clarification to the text:

"In contrast, they are easily observable by NMR, since the 1H and 13C spins at the δ and ε positions differ in chemical shift. A ring flip consequently leads to an interchange of $(CH)^{\varepsilon1}$ and $(CH)^{\varepsilon2}$ as well as $(CH)^{\delta1}$ and $(CH)^{\delta2}$, resulting in line broadening or exchange cross-peaks depending on the timescale (Figure 1B)."

18. Page 3, top of right column: the space group identifier is not properly formatted

We corrected the formatting of the space group identifiers.

19. On page 3 of the SI, the process of preparing the samples is discussed. Could one add an estimate of the amount (mg) of protein per sample, and the relative amount of water present? Also, what was the centrifugation used to pack the GB1-IgG complex?

We added estimates of the amount of sample filled into the rotors to the Supporting Information, on page 3. We have ca. 3 mg protein in a 1.3 mm and 12 mg in a 1.9 mm rotor. According to the crystal structure, we know that 46.6% of the crystal volume is water. In addition, it is common that there is some additional "supernatant" solvent in the rotor (see e.g. Figure 1 of Böckmann et al, 10.1007/s10858-009-9374-3). We also added a statement about the centrifugation of the complex at the same location in the SI, which was performed at 68000 rcf over night.

20. Page 12 of the SI: I suggest providing a bit more info on the source of the CSA tensor, and explicitly noting the relevant nucleus. (Question: does the orientation of the CSA relative to the bond direction matter?)

We have added the citations for the sources of the tensor parameters in the SI and also clarified the nuclei.

In general, the relative orientation of the CSA and the dipolar tensor matters. However, it is not trivial to model this, and it is assumed in the detectors analysis that the tensors are colinear. This is a valid assumption for the cases that we are studying here.

21. SI: please expand captions of SI figures S20-S22 to explain in more detail what is shown. (Perhaps this may also aid some of the other SI figures, to make sure that they are sufficiently clear on their own)

We added more explanation to these figure captions.

Reviewer #2 (Comments for the Author):

In my below review of the manuscript „Aromatic Ring Flips Reveal Reshaping of Protein Dynamics in Crystals and Complexes"by Becker and coworkers, I tried to closely follow the instructions and recommendations provided on https://www.nature.com/nature-portfolio/editorial-policies/peer-review

• Key results: Please summarise what you consider to be the outstanding features of the work.
The outstanding features of this work are, in my opinion, the very high-quality of the data and methodological approaches taken, the combination of high-fidelity biomolecular NMR experiments with state-of-the-art MD simulations, and the detailed interpretations of the results. The main results are that the crystal environment affects the protein dynamics (compared to, e.g., the protein in solution or also the protein in a binary complex with another protein binding partner) in a distinct way, as probed by the ring flips or aromatic side chains in the core of the small protein GB1, which serves as a model system in this study.

• Validity: Does the manuscript have flaws which should prohibit its publication? If so, please provide details.
There are no such flaws. The work has been carried out at an extremely high technical level, and the results are clearly and carefully presented, interpreted and discussed, with very strong evidence for the conclusions. The authors are to be commended not only for their impressive level of expertise, but also with the modest tone. We have a few questions, comments, and suggestions for further improvements, see below.

• Originality and significance: If the conclusions are not original, please provide relevant references. On a more subjective note, do you feel that the results presented are of immediate interest to many people in your own discipline, and/or to people from several disciplines?
This is of course not the first study that compares the dynamics of proteins in solution vs. in crystals. However, it stands out in terms of its technical quality and especially also in terms of the great level of detail, down to the atomic level.
I think that it is hard to judge whether it provides an "advance in understanding likely to influence thinking in the field". In general, the impact of the environment on the conformational ensembles and also on the dynamics of the structural transitions in proteins is still underappreciated (and sometimes even overlooked). Thus, I think that the present work is of significant interest.

• Data & methodology: Please comment on the validity of the approach, quality of the data and quality of presentation.
All outstanding, see above.

• Please note that we expect our reviewers to review all data, including any extended data and supplementary information. Is the reporting of data and methodology sufficiently detailed and transparent to enable reproducing the results?
Yes.

• Appropriate use of statistics and treatment of uncertainties: All error bars should be defined in the corresponding figure legends; please comment if that's not the case. Please include in your report a

specific comment on the appropriateness of any statistical tests, and the accuracy of the description of any error bars and probability values.
Appropriate statistical uncertainties/error estimates are reported and explained in the SI, but are not always defined in the figure legends. That could be easily added.
We thank the reviewer for pointing out this issue. We have now added statements explaining the error bars in the figure legends.

• Conclusions: Do you find that the conclusions and data interpretation are robust, valid and reliable?
Absolutely yes.

• Suggested improvements: Please list additional experiments or data that could help strengthening the work in a revision.

1. For a meaningful comparison of the dynamics of different systems (e.g., GB1 QDD vs. T2Q crystals, or GB1 crystal vs. IgG:GB1 complex), it is of course essential to consider them at the same temperature. Can the authors explicitly refer to that in the text (for example, but not necessarily only limited to, the data in Figure 2 and the corresponding discussion -- I consulted the two PhD theses from U Warwick (Refs 58 and 60) but could not quickly find the relevant information...)?
We thank the reviewer for pointing out that this information is missing in the manuscript. We added the temperatures for the different datasets to the caption of Figure 2. The data for $GB1^{QDD}$ were obtained at 304 K, while the other two datasets were obtained at 300 K. This temperature difference is negligible for our qualitative comparison of the dynamics in these systems.
See also ref. 59, p.177.

2. In the text, the authors equate entropy with “disorder”. Interpreting entropy as "disorder" is a simple and often applied analogy but can in many cases be misleading (also here, in our opinion). A smaller number of accessible microstates in a reduced/compressed configuration space volume might be a better way to think about entropy in this context.
We thank the reviewer for this suggestion. We have rewritten the discussion about the entropic contribution (p.11, top left and p. 15, left column) to clarify these possibly confusing notations. In particular, we have also changed our wording and included the concept that there are fewer microstates accessible, which is certainly a more precise way of expressing this, compared to just equating entropy with “disorder”. We thank the reviewer for this suggestion.

3. On p. 9 (right column), the authors conclude that “the transition states of the ring flips of Y3 and Y45 in crystals are less disordered compared to those in solution.” However, that is not necessarily so. Couldn’t it also be that the entropy of the ring flip transition state ensembles are the same in the Xtal and in solution, but that the initial state (the "educt minimum") is more ordered in the Xtal than in solution?
We agree with the reviewer that our statement about the entropy of the transition state was not accurate. We clarified this in the text and added a more detailed explanation in the discussion, see page 14, right column. Please also refer to our answer to point 9 of reviewer 1.

4. Further down in the same paragraph, they write that “A lower degree of disorder in the crystal is expected as the crystal lattice imposes constraints on the protein.” That statement is of course true, but in what way is this related to (or relevant for) the discussion of the larger dS of activation in the

crystal? Because the "lower degree of disorder" of the Xtal compared to the solution could, in principle, equally apply to both the equilibrium state (the minimum) and the transition state. In that case, the difference (Delta) would be the same as in solution.

We thank the reviewer for this comment and have removed this sentence on p.9 (now p.11). With the data at hand, we can only make statements about the barrier height in each system and not about the entropy differences between states in different systems.

5. I am puzzled by Figure 5C. Purple and green colors denote the data in the Xtal and in solution (at least that color code was used in the previous figures, but unfortunately also used in Fig. 5B in a different context). Then why isn't the dS of activation in the Xtal plotted in panel C, but only -TdS in solution?? As far as I understand, this information should be available from the fit shown in Figure 5B (which was done by constraining dH of activation in solution to be the same as dH of activation in the Xtal, as is written in the figure caption -- can the authors justify this assumption?).

The color code for solution and crystal is purple and green, respectively. In Figure 5B, two other colors were introduced to distinguish the two residues. We changed this to different symbols and line styles to keep the color code fully consistent throughout the manuscript. In Figure 5C, we are now plotting T∆S instead of -T∆S, which might have been misleading, and added labels to the columns. Indeed, T∆S is available for solution (purple) and crystal (green), and is plotted for both. The values for the crystal are very small, but we hope that the added labels help to spot them.

Regarding the justification of our assumption of equal ∆H in solution and in crystals, our rationale is the following:

- The slopes in Figure 5C indicate that ∆H(solution) ≥ ∆H(crystal), as justified by the fact that the slopes are similar: an equal slope would imply that ∆H(solution) = ∆H(crystal), while a smaller slope in the crystal would imply ∆H(solution) ≥ ∆H(crystal). See the discussion on page 9 (right column) and around Figure S9.
- We have also tested fits in which ∆H and ∆S are fitted independently, imposing only that ∆H(solution)≥∆H(crystal) and ∆S(solution)≥ ∆S(crystal); these assumptions naturally follow from the slopes, as discussed above. While such a fit is not ideal, as we fit two parameters from two observables, the resulting fit shows at least qualitatively the same conclusion, namely that ∆S(solution)>∆S(crystal). Please find the fitted values below:

fit constraint

$\Delta H_s = \Delta H_c$

Y3	$\Delta^{\ddagger}H$	$\Delta^{\ddagger}S$
solution	65 ± 3	54± 11
crystal		2 ± 11

Y45	$\Delta^{\ddagger}H$	$\Delta^{\ddagger}S$
solution	68 ± 1	63 ± 4
crystal		3 ± 4

$\Delta H_s \geq \Delta H_c$ $\Delta S_s \geq \Delta S_c$

Y3	$\Delta^{\ddagger}H$	$\Delta^{\ddagger}S$
solution	65 ± 3	52 ± 10
crystal	65 ± 2	4 ± 5

Y45	$\Delta^{\ddagger}H$	$\Delta^{\ddagger}S$
solution	69 ± 8	66 ± 13
crystal	69 ± 4	11 ± 12

6. Another puzzling aspect of Figure 5C is that -TdS of activation is negative, that is, the entropic contribution to the activation free energy barrier is favorable. How can that be reconciled with the transition state having "reduced disorder" (= lower entropy)?

We apologize for the somewhat confusing manner in which we presented the data in the previous version. We are now plotting T∆S in Figure 5C and have changed the caption of that paragraph to “Ring flips in crystals are less entropically favoured”.

7. Can the authors comment on why the energy profiles in Figure 5D are skewed (the green and purple curves for Y3 in the left panel and the purple curve (solution data) for Y45 in the middle panel)?

There are a number of physical reasons why the free-energy profile does not need to be fully symmetrical. The first lies in the functional form of the dihedral angles in the force field. Most academic classical force fields, in general, and the CHARMM force field, in particular, use a sum of cosine terms with different multiplicities and phases, as opposed to a single simple $U(\varphi)=U_0(1+\cos(n\varphi-\varphi_0))$. This superposition of terms easily produces asymmetric wells and skewed barrier shapes. The second lies in the coupling to other degrees of freedom. The dihedral angle rarely moves alone. Valence angles and neighboring dihedral angles along the side chain, together with backbone adjustments, change with φ, and have different densities of states on either side. This can be viewed as a state-dependent entropy contribution, likely to introduce skewness to the free energy. A third reason is rooted in the nonbonded interactions and the stereochemistry, with possible steric clashes, gauche versus trans interactions, and transient intramolecular hydrogen bonds which can result in one flank of a well steeper or shallower than the other. Fourth, in explicit solvent, orientation of certain polar groups relative to the solvent, as well as solvation shells, and ion-binding sites can promote asymmetric stabilization for certain values of φ. Fifth and last, in connection with the second argument, entropic contributions necessarily vary with φ. Even if the mean potential energy were symmetric, a φ-dependent entropy makes the free energy along φ asymmetric. Narrow, deep wells have lower entropy than broad, shallow wells. Skewness or asymmetry in the free-energy profile reflects a variation in the entropy term across the collective variable, φ. Deep wells tend to be associated to larger entropy gradients.

We have added a statement to the figure legend to address this topic.

8. The authors should compute also dH and dS of activation (for example, from a series of free energy simulations at different T). That would be important to confirm the interpretations of the experimental data in terms of increased activation entropy (but not activation energy) in the Xtal compared to in solution.

While estimation of ∆S and ∆H by means of computer simulations would be desirable, especially in the present case, the resulting estimates are likely to be burdened by very large errors, making them in general meaningless. To compute the entropy using geometric free-energy calculations, as we did for the isomerization of the aromatic side chains, we often turn to the relationship $\Delta S = -\partial\Delta A/\partial T$, at constant N and V, which we handle in terms of finite differences, that is, $\Delta S(T) = -[\Delta A(T + \Delta T) - \Delta A(T - \Delta T)] / 2\Delta T$. The choice of ∆T is, however, far from trivial. If ∆T is too small, then the difference $\Delta A(T + \Delta T) - \Delta A(T - \Delta T)$ will be on the order of the uncertainty in the free-energy estimates. Conversely, if ∆T is too large, the finite-difference approximation is no longer valid.

Even with extremely long simulations, obtaining a reliable estimate of the entropy for the system at hand is essentially infeasible. First, the true uncertainty in our free-energy calculations is almost

surely much greater than the statistical error bars reported in the manuscript, since the degree to which the simulations have explored the orthogonal degrees of freedom—specifically, the slow modes that are coupled to χ_2—is largely indeterminate. Even with an ergodic-sampling strategy consisting of multiple walkers exploring the collective variable concomitantly and exchanging information on the gradients measured locally, overcoming hidden barriers in the orthogonal manifold remains computationally challenging. Expecting to resolve subtle temperature-dependent variations in this orthogonal sampling within a multi-µs, albeit finite simulation window, and for such effects to be reflected accurately in the free-energy profile at sub-kcal/mol precision, is, therefore, unrealistic.

Table 2. Free Energy and Entropy Estimation for Protein−Ligand Binding and Alcohol Hydration

molecular system	quantity (kcal/mol)	T (K)	value (kcal/mol)	method
p41:Abl-SH3	ΔG°_{bind}(calc)	295	−8.3 ± 0.3	WTM-eABF (N_{runs} = 12, 1.3 µs)[b]
	ΔG°_{bind}(calc)	300	−8.0 ± 0.2	WTM-eABF (N_{runs} = 4, 440 ns)[b]
	ΔG°_{bind}(calc)	305	−7.9 ± 0.1	WTM-eABF (N_{runs} = 5, 550 ns)[b]
	$T\Delta S^\circ_{bind}$(calc)	300	−12.6 ± 11	eq 3
	ΔG°_{bind}(exp)[a]	300	−7.99 ± 0.04	
	$T\Delta S^\circ_{bind}$(exp)[c]	300	−14.20	
ethanol hydration	$T\Delta S_{solv}$(calc)	298	−7.0 ± 11.9	MW-ABF (2.4 µs)
	$T\Delta S_{solv}$(calc)	298	−7.7 ± 12.8	WTM-eABF (3 µs)
	$T\Delta S_{solv}$(calc)	298	−7.4 ± 0.5	REX-WTM-eABF (750 ns)
	$T\Delta S_{solv}$(exp)[d]	298	−7.0	

[a]From ref 61. [b]N_{runs} is the number of parallel runs. The time for each simulation was 100−150 ns. [c]From ref 62. [d]From ref 63.

To substantiate our reluctance to undertake the suggested additional simulations, we refer the Reviewer to calculations published in Accounts of Chemical Research (Fu et al. Acc. Chem. Res. 2019, 52, 3254−3264) for two objectively simpler systems—ethanol hydration and the binding of a proline-rich peptide (p41) to the SH3 domain of the Abl kinase—where sampling in the orthogonal manifold is only a minor concern,
although the entropy estimates themselves appear to be reasonable, and agree fairly well with the experimental values, the very large associated uncertainties, greater than the actual values of the TΔS terms, remain problematic and inevitably cast doubt on the reliability of the results. While it is possible that our TΔS estimates are adequately converged, the magnitude of the error bars makes it difficult to assert it with confidence.

9. On p. 8 (right column), the authors write that "intermolecular contacts favor more extended states of GB1 by loosening the hydrophobic core". Do they mean more "expanded states" or "less compact states" maybe? "Extended" implies elongated, which might not be exactly what the authors want to say here?

We have rewritten that sentence for clarification. Please refer to point 6 of reviewer 1 for a more detailed answer.

• References: Does this manuscript reference previous literature appropriately? If not, what references should be included or excluded?

References are appropriate.

• Clarity and context: Is the abstract clear, accessible? Are abstract, introduction and conclusions appropriate?

Yes.

Reviewer #3 (Comments for the Author):

The manuscript by Becker, et al reports an NMR study of aromatic ring flipping kinetics in a crystallized protein (GB1), providing a comparison to the behavior of the protein in solution as a monomer and in complex with a binding partner (IgG). The authors implement a clever metabolic labeling methodology that incorporates 13C into the Cε positions of Phe and Tyr residues, which enables relatively straightforward measurement of ring flipping kinetics over a range of timescales using a battery of different NMR relaxation experiments. The results show that ring flipping kinetics are slowed significantly when the protein is in the crystalline phase relative to wen in solution, and significantly accelerated when the protein is in solution as a heterodimeric complex. A classical Eyring analysis shows that when the protein is crystallized, there is a large decrease in the entropy of activation for ring flips, which results in the observed reduction of the rate constant. By extension, the authors propose that in the GB1:IgG complex, GB1 is trapped in an "extended" conformation that lowers the activation energy for ring flipping. Analysis of MD simulations of the protein in solution and in the crystal provide additional insight into how the aromatic ring flips are coupled to the global dynamic of the protein in both phases, and illustrate how the internal dynamics are perturbed by the crystal lattice.

All the experiments and analyses in the paper appear to have been performed rigorously, and I have no major technical concerns with the manuscript. I also would like to mention that the introduction to this paper is very well written and was interesting to read. Overall, I think this paper is an excellent demonstration of how a protein's dynamics can be perturbed by its environment. There is a clear cautionary message for experimenters who use crystallographic approaches to study protein motions, but the results also provide additional evidence that molecular interactions, broadly speaking, can influence the internal dynamics of proteins, which is a fundamentally important concept in biophysics. These insights will be of interest to the Nature Chemistry audience, which includes structural biologists and biophysicists, and I recommend publication with minor revisions. In particular, I note that Nature Chemistry has a strong track record of publishing high-impact papers in the field of time-resolved crystallography, and this manuscript is especially relevant to this audience.

I have only one major technical concern. The crystal structure reported by the authors, which is subsequently used for a B-factor analysis and for launching MD simulations was determined at cryogenic temperatures. Looking at the unit cell parameter calculated for the single crystal (cryo) and powder diffraction (room temp) experiments shows a significant contraction of the unit cell due to cryocooling, and I wonder whether this could affect the interpretation. Given that single crystal experiments are trivial at room temperature, and that the authors have microcrystal in hand that could facilitate serial data collection, I wonder why a room temperature crystal structure was not used for the analysis?

We thank the reviewer for this comment. For this revised version, we determined a single crystal X-ray structure at 295 K (deposited as PDB 9T8Z). However, we believe that the cryo-temperature crystal structure is still a valid, and arguably better, starting point for the MD simulations and for mechanistic considerations, for the following reasons.
The reservoir buffer for the crystallization contains 25% isopropanol, which is volatile, and we found that the crystals were very fragile at room temperature due to isopropanol evaporation and dehydration. While for NMR rotors this is not a problem – the rotors are closed tightly to avoid any evaporation – it can cause problems for crystallography. This was less of a problem for the structure obtained at cryogenic conditions. Consequently, the resolution of the room temperature structure is

slightly lower. There might also be some structural alterations, possibly, due to evaporation; we see a weak density that might indicate a low-populated conformation of Y33, for example (at 0.7 σ).

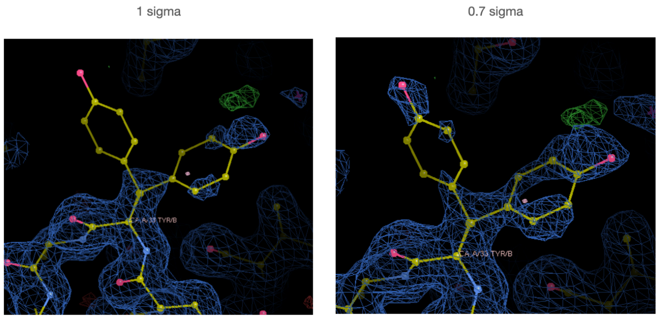

Therefore, it seems safer to use the 100 K structure for the analyses and as outlined below, the relaxed MD structure reflects the physical situation very well, in our opinion.

Besides possible complications with solvent evaporation, the newly determined room-temperature structure is useful in the sense that it shows the expected trends in terms of unit cell size. We find the following values (a, b, c, β), which are compared here also to other values we obtained in this study:

- SC XRD, 100 K: 77.48 Å, 35.18 Å, 49.94 Å, 122.51°
- SC XRD, 295 K: 78.70 Å, 35.41 Å, 51.82 Å, 122.60°
- Powder XRD: 77.9 Å, 35.3 Å, 51.4 Å, 122.3°
- MD after energy minimization: 80.36 Å, 36.49 Å, 53.24 Å, 120.71°

The thermal expansion is in line with the expectation (https://doi.org/10.1021/bi00375a035).

Importantly, in the equilibration simulations for MD simulation, which used the 100 K crystal structure as a starting point, the simulated lattice reached unit cell parameters that are close to those found in the 295 K structure, suggesting that our simulations are a good proxy for the behavior at ambient temperature. For these reasons, having collected now both a 100 K and a 295 K structure, we believe that the MD simulations that use the former are valid.

I also have the following minor suggestions for the authors:

• The manuscript focuses on the kinetics of ring flipping, but I wonder whether the NMR data also contain information about the equilibrium populations of different ring conformations for GB1 in solution and in the crystal? While the differences in kinetics are important, especially with respect to time-resolved crystallography, differences in equilibrium populations are arguably more important, because the occupancies of alternative conformations are commonly refined in static crystallography experiments as well (especially at room temperature). Static crystallography experiments are far more common, and this additional insight could increase the impact of the paper if the authors can provide data and commentary.

A ring flip is a symmetric motion in which the two states cannot be distinguished structurally (see also Figure 1A), and the populations of the two states are always 50 %. The strength of NMR in this case lies in its ability to study the dynamics of this process, even though the two states are structurally indistinguishable. This is because the chemical environment of $(CH)^{\varepsilon 1}$ and $(CH)^{\varepsilon 2}$ is not identical, resulting in unique chemical shifts for the two sites.

For non-symmetric processes, it would be possible to obtain information about the populations of different states by NMR. However, none were observed, both in solution and in crystals.

• I appreciate that many authors are reluctant to speculate beyond the data reported in their manuscript, but I think it could be useful to discuss how these results might extrapolate to other systems. For example, will the crystal lattice always restrict dynamics? Based on the findings reported for the GB1:IgG complex, could there be situations where a protein crystalizes in a conformation that accelerates internal dynamics? I want to emphasize that I am not suggesting any additional experiments on other systems, but some mention of how widespread the authors expect this phenomenon to be would be useful.

We thank the reviewer for this suggestion. Proteins are incredibly versatile and complex molecules that are, by design, very sensitive to interactions with other molecules and their surroundings. We imagine that the effects of a crystal lattice on the dynamics of the protein can be just as diverse. The exact characteristics of these effects most likely strongly depends on factors like the density of the packing, the solvent contents and the amount of specific intermolecular crystal contacts. In our opinion, it is conceivable that crystal packing could also lead to an acceleration of internal dynamics, e.g. by stabilizing such states in which ring flips are facilitated. Even though this is of course highly speculative, it would not change the message of our work: one should be careful to make conclusions about protein dynamics measured in such an unnatural environment as a crystal.

We have added the following statement to the discussion (p.15-16):

"It is tempting to extrapolate our findings on GB1 to other crystalline systems. Since crystalline environments are inherently more densely packed than the protein's solution environment, ring flips in many crystals are likely slower than in solution. However, the specifics may depend heavily on context, as demonstrated by our IgG:GB1 results. If intermolecular contacts favor conformations that facilitate ring flips, the crystalline environment might even accelerate them. Even for a single protein in different crystal lattices, ring flip dynamics may vary, just like overall rocking motion, which also depends on packing density.[35]"

• Figure 2B could be improved by performing a B-factor normalization. Clearly, the overall B-factor is much higher for the GB1QDD than for the GB1T2Q crystal. Normalization could make these data more easily comparable across different crystal forms. See, for example Barthels, et al. (2020) Mol. Inf. and/or Carugo (2022) Acta Cryst. D.

We used the tool by Barthels et al. (2020) to normalize the B-factors in Figure 2B. The raw B-factors can now be found in Figure S2B. We thank the reviewer for this suggestion.

Additional changes

We have further made the following minor changes to the manuscript.

- We have added an author, Daniel M. Balazs, who contributed to the crystallographic work. This change reflects the increased fraction of the crystallographic data in this revised version.
- We have deposited the NMR assignment of $GB1^{QDD}$ at the BMRB under the access code 53330. During the deposition, we discovered a mistake in the referencing of the 13C dimensions in the assignment (shift by 2.66 ppm), which we corrected in the respective parts of the manuscript.
- We have added a Data Availability statement to the manuscript.

Reviewer #1:
Comments for the Author:
I thank the authors for their thoughtful responses to the comments from myself and the other two reviewers. The raised questions and comments have been addressed and/or improved in the revised manuscript. I have no significant open questions left.

Except for one small point that could be made clearer, in my view. In my point #17 we discussed the shift differences of the epsilon and delta sites. The revised text is improved. It does still retain the sentence "In contrast, they are easily observable by NMR, since the 1 H and 13 C spins at the δ and ε positions differ in chemical shift. " I still worry that people may read this incorrectly, as emphasizing that the shift of the epsilon site differs from that of the delta site. Naturally these shifts are indeed different, but the point here is rather that CE1 and CE2 differ from each other, not that they differ from CD1/CD2.... A rephrasing of the retained sentence could try to avoid this possible misreading?

We thank the reviewer for their positive feedback.
We edited the sentence in the following way and hope that this is clearer now:
"In contrast, they are easily observable by NMR, since the 1H and 13C spins on each side of the aromatic ring (e.g. ε1 and ε2) differ in chemical shift."

Reviewer #2:
Comments for the Author:
We are satisfied with the changes made. It is especially nice to see that the authors determined an Xray structure also at room temperature. The authors are to be commended for their careful work and their conclusive and important results.

We thank the reviewer for their positive feedback.

Reviewer #3:
Comments for the Author:
I am satisfied that the revised version of the manuscript and the rebuttal from the authors adequately address my concerns. I appreciate the challenges related to crystallization conditions involving volatile solvents, and I apologize for my oversight regarding the symmetry of the ring flips as it pertains to equilibrium populations.

We thank the reviewer for their positive feedback.
